# Supplementary material for: The Blood Plasma Lipidomic Profile in Atherosclerosis of the Brachiocephalic Arteries
Source: Biomedicines. 2024 Jun 9;12(6):1279. doi: 10.3390/biomedicines12061279 (PMC11201046; doi:10.3390/biomedicines12061279)
Supplement: Supplementary file 1 [file biomedicines-12-01279-s001.zip › biomedicines-3048851-supplementary.pdf]

| Subject | 0 | 1 | Significance, p |
|---|---|---|---|
| **Index** | | | 0.143 |
| Mean ± Standard deviation | 29.5 ± 16.1 | 37.1 ± 22.3 | |
| Median and [25%; 75%] | 29.5 [17.3; 42.8] | 44.0 [12.8; 56.3] | |
| **Group** | | | <0.001 |
| Control | 2 (4.3%) | 8 (50.0%) | |
| Main | 44 (95.7%) | 8 (50.0%) | |
| **Sex** | | | 0.319 |
| Female | 10 (21.7%) | 6 (37.5%) | |
| Male | 36 (78.3%) | 10 (62.5%) | |
| **Age, years** | | | 0.001 |
| Mean ± Standard deviation | 66.1 ± 11.3 | 52.1 ± 14.4 | |
| Median and [25%; 75%] | 68.0 [63.0; 72.8] | 49.5 [41.0; 64.5] | |
| **Smoking** | | | 0.111 |
| 0 | 24 (52.2%) | 12 (75.0%) | |
| 1 | 22 (47.8%) | 4 (25.0%) | |
| **Diabetes melitus** | | | 0.026 |
| 0 | 34 (73.9%) | 16 (100.0%) | |
| 1 | 12 (26.1%) | 0 (0.0%) | |
| **Arterial Hypertension** | | | <0.001 |
| 0 | 3 (6.5%) | 8 (50.0%) | |
| 1 | 43 (93.5%) | 8 (50.0%) | |
| **Coronary heart disease** | | | 0.355 |
| 0 | 31 (67.4%) | 13 (81.3%) | |
| 1 | 15 (32.6%) | 3 (18.8%) | |
| **Ischemic Stroke** | | | 0.200 |
| 0 | 32 (69.6%) | 14 (87.5%) | |
| 1 | 14 (30.4%) | 2 (12.5%) | |
| Sinus rhythm | | | |
| 1 | 46 (100.0%) | 16 (100.0%) | |
| **Heart failure** | | | 0.565 |
| 0 | 42 (91.3%) | 16 (100.0%) | |
| 1 | 4 (8.7%) | 0 (0.0%) | |
| **Chronic Kidney Disease** | | | 0.422 |
| 0 | 7 (15.2%) | 3 (18.8%) | |
| 1 | 2 (4.3%) | 3 (18.8%) | |

| | | | |
|---|---|---|---|
| 2 | 17 (37.0%) | 5 (31.3%) | |
| 3a | 18 (39.1%) | 5 (31.3%) | |
| 3б | 2 (4.3%) | 0 (0.0%) | |
| Chronic Kidney Failure | | | |
| 0 | 46 (100.0%) | 16 (100.0%) | |
| **Hemoglobine g/l** | | | 0.541 |
| Mean ± Standard deviation | 134.1 ± 14.0 | 137.2 ± 18.3 | |
| Median and [25%; 75%] | 135.0 [124.3; 143.8] | 137.5 [130.0; 145.5] | |
| **RBC 10*12/l** | | | 0.916 |
| Mean ± Standard deviation | 4.6 ± 0.4 | 4.6 ± 0.5 | |
| Median and [25%; 75%] | 4.6 [4.3; 5.0] | 4.6 [4.3; 4.8] | |
| **WBC 10*9/l** | | | 0.334 |
| Mean ± Standard deviation | 7.4 ± 3.0 | 6.8 ± 2.2 | |
| Median and [25%; 75%] | 7.0 [5.7; 8.1] | 6.3 [5.1; 7.6] | |
| **PLT 10*9/l** | | | 0.954 |
| Mean ± Standard deviation | 254.9 ± 56.1 | 254.0 ± 53.3 | |
| Median and [25%; 75%] | 246.0 [213.4; 292.0] | 242.5 [211.8; 299.1] | |
| **ESR mm/h** | | | 0.077 |
| Mean ± Standard deviation | 24.0 ± 17.7 | 18.9 ± 22.5 | |
| Median and [25%; 75%] | 19.0 [11.0; 30.0] | 9.0 [6.0; 20.5] | |
| **Specific gravity** | | | 0.839 |
| Mean ± Standard deviation | 998.0 ± 135.3 | 1,017.4 ± 5.1 | |
| Median and [25%; 75%] | 1,017.0 [1,015.0; 1,020.0] | 1,016.5 [1,015.0; 1,020.3] | |
| **Erythrocyturia per field** | | | 0.315 |
| 0 | 41 (89.1%) | 16 (100.0%) | |
| 1 | 5 (10.9%) | 0 (0.0%) | |
| **Leukocyturia  fer field** | | | 0.743 |
| 0 | 36 (78.3%) | 12 (75.0%) | |
| 1 | 10 (21.7%) | 4 (25.0%) | |
| **Bacteriuria** | | | 0.725 |
| 0 | 37 (80.4%) | 12 (75.0%) | |
| 1 | 9 (19.6%) | 4 (25.0%) | |
| **Glucosuria** | | | >0.999 |
| 0 | 43 (97.7%) | 15 (100.0%) | |
| 1 | 1 (2.3%) | 0 (0.0%) | |
| **Proteinuria** | | | 0.453 |

| | | | |
|---|---|---|---|
| 0 | 45 (97.8%) | 15 (93.8%) | |
| 1 | 1 (2.2%) | 1 (6.3%) | |
| **Salts in urine** | | | 0.315 |
| 0 | 41 (89.1%) | 16 (100.0%) | |
| 1 | 5 (10.9%) | 0 (0.0%) | |
| **Total protein g/l** | | | 0.924 |
| Mean ± Standard deviation | 72.0 ± 4.5 | 72.1 ± 5.4 | |
| Median and [25%; 75%] | 72.0 [69.2; 74.6] | 70.5 [68.8; 74.2] | |
| **Creatinine µmol/l** | | | 0.558 |
| Mean ± Standard deviation | 101.3 ± 21.1 | 97.4 ± 23.0 | |
| Median and [25%; 75%] | 101.5 [87.0; 111.0] | 92.0 [79.8; 116.5] | |
| **CKD MDRD ml/min/1,73m2** | | | 0.454 |
| Mean ± Standard deviation | 65.7 ± 15.6 | 71.2 ± 19.6 | |
| Median and [25%; 75%] | 63.8 [54.3; 74.2] | 63.9 [57.9; 85.7] | |
| **Urea µmol/l** | | | 0.215 |
| Mean ± Standard deviation | 7.0 ± 2.2 | 6.2 ± 1.4 | |
| Median and [25%; 75%] | 6.6 [5.5; 7.9] | 5.7 [5.5; 6.9] | |
| **Uric acid µmol/l** | | | 0.484 |
| Mean ± Standard deviation | 343.1 ± 105.9 | 309.7 ± 86.8 | |
| Median and [25%; 75%] | 335.9 [288.8; 389.5] | 334.4 [208.8; 361.6] | |
| Mean ± Standard deviation | | | 0.816 |
| Mean ± Standard deviation | 8.2 ± 15.4 | 6.2 ± 7.6 | |
| Median and [25%; 75%] | 2.9 [1.2; 6.4] | 2.6 [0.8; 9.8] | |
| **Glucose  µmol/l** | | | 0.949 |
| Mean ± Standard deviation | 5.7 ± 2.0 | 5.3 ± 0.5 | |
| Median and [25%; 75%] | 5.3 [4.9; 5.8] | 5.2 [5.0; 5.5] | |
| **AST points/l** | | | 0.897 |
| Mean ± Standard deviation | 22.4 ± 6.4 | 23.4 ± 9.7 | |
| Median and [25%; 75%] | 21.5 [18.3; 26.8] | 19.5 [16.0; 30.0] | |
| **ALT points/l** | | | 0.484 |
| Mean ± Standard deviation | 21.3 ± 8.4 | 26.9 ± 15.6 | |
| Median and [25%; 75%] | 19.5 [15.3; 26.0] | 21.0 [15.0; 41.3] | |
| **Total bilirubine  µmol/l** | | | 0.974 |
| Mean ± Standard deviation | 11.8 ± 4.2 | 11.9 ± 4.9 | |
| Median and [25%; 75%] | 10.9 [9.7; 12.4] | 10.9 [9.3; 14.0] | |
| **Iron µmol/l** | | | 0.250 |

| | | | |
|---|---|---|---|
| Mean ± Standard deviation | 14.7 ± 7.9 | 17.5 ± 8.0 | |
| Median and [25%; 75%] | 12.7 [8.1; 21.7] | 15.7 [11.7; 24.1] | |
| **Fibrinogen g/l** | | | 0.068 |
| Mean ± Standard deviation | 3.9 ± 1.1 | 3.4 ± 1.5 | |
| Median and [25%; 75%] | 3.7 [3.3; 4.3] | 3.1 [2.5; 4.0] | |
| **APTT** | | | 0.477 |
| Mean ± Standard deviation | 1.1 ± 0.2 | 1.1 ± 0.1 | |
| Median and [25%; 75%] | 1.0 [1.0; 1.1] | 1.0 [1.0; 1.1] | |
| **INR** | | | 0.100 |
| Mean ± Standard deviation | 1.1 ± 0.1 | 1.0 ± 0.0 | |
| Median and [25%; 75%] | 1.1 [1.0; 1.1] | 1.0 [1.0; 1.0] | |
| **Quick's value** | | | 0.325 |
| Mean ± Standard deviation | 94.0 ± 10.7 | 96.6 ± 8.1 | |
| Median and [25%; 75%] | 93.0 [87.0; 101.0] | 94.0 [91.0; 99.5] | |
| **Total cholesterol µmol/l** | | | 0.257 |
| Mean ± Standard deviation | 4.6 ± 1.2 | 5.0 ± 1.2 | |
| Median and [25%; 75%] | 4.4 [4.0; 5.5] | 4.7 [4.3; 5.4] | |
| **Triglycerides µmol/l** | | | 0.607 |
| Mean ± Standard deviation | 1.6 ± 0.8 | 1.7 ± 0.9 | |
| Median and [25%; 75%] | 1.3 [1.1; 1.9] | 1.6 [1.1; 2.0] | |
| **LDL µmol/l** | | | 0.365 |
| Mean ± Standard deviation | 2.8 ± 0.9 | 3.1 ± 1.0 | |
| Median and [25%; 75%] | 2.8 [2.2; 3.5] | 3.0 [2.4; 3.6] | |
| **VLDL µmol/l** | | | 0.904 |
| Mean ± Standard deviation | 0.8 ± 0.5 | 0.7 ± 0.3 | |
| Median and [25%; 75%] | 0.7 [0.5; 0.9] | 0.7 [0.5; 0.8] | |
| **HDL µmol/l** | | | 0.174 |
| Mean ± Standard deviation | 1.2 ± 0.4 | 1.2 ± 0.3 | |
| Median and [25%; 75%] | 1.1 [0.9; 1.3] | 1.2 [1.1; 1.4] | |
| **Atherogenic coefficient** | | | 0.726 |
| Mean ± Standard deviation | 3.2 ± 1.3 | 3.1 ± 1.2 | |
| Median and [25%; 75%] | 3.2 [2.4; 4.2] | 3.0 [2.1; 3.7] | |
| **TSH µIU/l** | | | 0.693 |
| Mean ± Standard deviation | 1.9 ± 1.4 | 1.9 ± 1.2 | |
| Median and [25%; 75%] | 1.4 [1.1; 2.0] | 1.5 [1.2; 2.7] | |
| **C12.0_1_percmol** | | | <0.001 |

| | | | |
|---|---|---|---|
| Mean ± Standard deviation | 0.0 ± 0.0 | 0.0 ± 0.0 | |
| Median and [25%; 75%] | 0.0 [0.0; 0.0] | 0.0 [0.0; 0.0] | |
| **C14.0_1_percmol** | | | <0.001 |
| Mean ± Standard deviation | 0.0 ± 0.0 | 0.0 ± 0.0 | |
| Median and [25%; 75%] | 0.0 [0.0; 0.0] | 0.0 [0.0; 0.0] | |
| **C16.0_1_percmol** | | | 0.598 |
| Mean ± Standard deviation | 0.2 ± 0.0 | 0.3 ± 0.0 | |
| Median and [25%; 75%] | 0.3 [0.2; 0.3] | 0.3 [0.2; 0.3] | |
| **C18.0_1_percmol** | | | 0.200 |
| Mean ± Standard deviation | 0.1 ± 0.0 | 0.1 ± 0.0 | |
| Median and [25%; 75%] | 0.1 [0.1; 0.1] | 0.1 [0.1; 0.1] | |
| **C20.0_1_percmol** | | | 0.384 |
| Mean ± Standard deviation | 0.0 ± 0.0 | 0.0 ± 0.0 | |
| Median and [25%; 75%] | 0.0 [0.0; 0.0] | 0.0 [0.0; 0.0] | |
| **C22.0_1_percmol** | | | 0.654 |
| Mean ± Standard deviation | 0.0 ± 0.0 | 0.0 ± 0.0 | |
| Median and [25%; 75%] | 0.0 [0.0; 0.0] | 0.0 [0.0; 0.0] | |
| **C24.0_1_percmol** | | | 0.319 |
| Mean ± Standard deviation | 0.0 ± 0.0 | 0.0 ± 0.0 | |
| Median and [25%; 75%] | 0.0 [0.0; 0.0] | 0.0 [0.0; 0.0] | |
| **C16.1_cis_1_percmol** | | | 0.496 |
| Mean ± Standard deviation | 0.0 ± 0.0 | 0.0 ± 0.0 | |
| Median and [25%; 75%] | 0.0 [0.0; 0.0] | 0.0 [0.0; 0.0] | |
| **C18.1_cis_1_percmol** | | | 0.916 |
| Mean ± Standard deviation | 0.3 ± 0.0 | 0.3 ± 0.0 | |
| Median and [25%; 75%] | 0.3 [0.2; 0.3] | 0.3 [0.2; 0.3] | |
| **C20.1_cis_1_percmol** | | | 0.703 |
| Mean ± Standard deviation | 0.0 ± 0.0 | 0.0 ± 0.0 | |
| Median and [25%; 75%] | 0.0 [0.0; 0.0] | 0.0 [0.0; 0.0] | |
| **C24.1_cis_1_percmol** | | | 0.001 |
| Mean ± Standard deviation | 0.0 ± 0.0 | 0.0 ± 0.0 | |
| Median and [25%; 75%] | 0.0 [0.0; 0.0] | 0.0 [0.0; 0.0] | |
| **C16.1_trans_1_percmol** | | | 0.250 |
| Mean ± Standard deviation | 0.0 ± 0.0 | 0.0 ± 0.0 | |
| Median and [25%; 75%] | 0.0 [0.0; 0.0] | 0.0 [0.0; 0.0] | |
| **C18.1_trans_1_percmol** | | | 0.883 |

| | | | |
|---|---|---|---|
| Mean ± Standard deviation | 0.0 ± 0.0 | 0.0 ± 0.0 | |
| Median and [25%; 75%] | 0.0 [0.0; 0.0] | 0.0 [0.0; 0.0] | |
| **C18.2_trans_1_percmol** | | | 0.883 |
| Mean ± Standard deviation | 0.0 ± 0.0 | 0.0 ± 0.0 | |
| Median and [25%; 75%] | 0.0 [0.0; 0.0] | 0.0 [0.0; 0.0] | |
| **C18.2_n_6_1_percmol** | | | 0.672 |
| Mean ± Standard deviation | 0.2 ± 0.0 | 0.2 ± 0.0 | |
| Median and [25%; 75%] | 0.2 [0.2; 0.3] | 0.2 [0.2; 0.3] | |
| **C18.3_n_6_1_percmol** | | | 0.366 |
| Mean ± Standard deviation | 0.0 ± 0.0 | 0.0 ± 0.0 | |
| Median and [25%; 75%] | 0.0 [0.0; 0.0] | 0.0 [0.0; 0.0] | |
| **C20.2_n_6_1_percmol** | | | 0.148 |
| Mean ± Standard deviation | 0.0 ± 0.0 | 0.0 ± 0.0 | |
| Median and [25%; 75%] | 0.0 [0.0; 0.0] | 0.0 [0.0; 0.0] | |
| **C20.3_n_6_1_percmol** | | | 0.635 |
| Mean ± Standard deviation | 0.0 ± 0.0 | 0.0 ± 0.0 | |
| Median and [25%; 75%] | 0.0 [0.0; 0.0] | 0.0 [0.0; 0.0] | |
| **C20.4_n_6_1_percmol** | | | 0.084 |
| Mean ± Standard deviation | 0.1 ± 0.0 | 0.1 ± 0.0 | |
| Median and [25%; 75%] | 0.1 [0.1; 0.1] | 0.1 [0.0; 0.1] | |
| **C22.4_n_6_1_percmol** | | | 0.411 |
| Mean ± Standard deviation | 0.0 ± 0.0 | 0.0 ± 0.0 | |
| Median and [25%; 75%] | 0.0 [0.0; 0.0] | 0.0 [0.0; 0.0] | |
| **C22.5_n_6_1_percmol** | | | 0.564 |
| Mean ± Standard deviation | 0.0 ± 0.0 | 0.0 ± 0.0 | |
| Median and [25%; 75%] | 0.0 [0.0; 0.0] | 0.0 [0.0; 0.0] | |
| **EPA_C20.5_n_3_1_percmol** | | | >0.999 |
| Mean ± Standard deviation | 0.0 ± 0.0 | 0.0 ± 0.0 | |
| Median and [25%; 75%] | 0.0 [0.0; 0.0] | 0.0 [0.0; 0.0] | |
| **DHA_C22.6n3_2_percmol** | | | 0.077 |
| Mean ± Standard deviation | 0.0 ± 0.0 | 0.0 ± 0.0 | |
| Median and [25%; 75%] | 0.0 [0.0; 0.0] | 0.0 [0.0; 0.0] | |
| **SFA_relat** | | | 0.060 |
| Mean ± Standard deviation | 0.3 ± 0.0 | 0.4 ± 0.0 | |
| Median and [25%; 75%] | 0.3 [0.3; 0.3] | 0.4 [0.3; 0.4] | |
| **UFA_relat** | | | 0.060 |

| | | | |
|---|---|---|---|
| Mean ± Standard deviation | 0.7 ± 0.0 | 0.6 ± 0.0 | |
| Median and [25%; 75%] | 0.7 [0.7; 0.7] | 0.6 [0.6; 0.7] | |
| **MUFA_relat** | | | 0.970 |
| Mean ± Standard deviation | 0.3 ± 0.0 | 0.3 ± 0.0 | |
| Median and [25%; 75%] | 0.3 [0.3; 0.3] | 0.3 [0.3; 0.3] | |
| **PUFA_relat** | | | 0.159 |
| Mean ± Standard deviation | 0.4 ± 0.0 | 0.3 ± 0.0 | |
| Median and [25%; 75%] | 0.4 [0.3; 0.4] | 0.3 [0.3; 0.4] | |
| **HUFA_relat** | | | 0.069 |
| Mean ± Standard deviation | 0.1 ± 0.0 | 0.1 ± 0.0 | |
| Median and [25%; 75%] | 0.1 [0.1; 0.1] | 0.1 [0.1; 0.1] | |
| **omega_6_relat** | | | 0.280 |
| Mean ± Standard deviation | 0.3 ± 0.0 | 0.3 ± 0.0 | |
| Median and [25%; 75%] | 0.3 [0.3; 0.4] | 0.3 [0.3; 0.3] | |
| **omega_3_index_relat** | | | 0.212 |
| Mean ± Standard deviation | 0.0 ± 0.0 | 0.0 ± 0.0 | |
| Median and [25%; 75%] | 0.0 [0.0; 0.0] | 0.0 [0.0; 0.0] | |
| **AAEPA_relat** | | | 0.163 |
| Mean ± Standard deviation | 18.6 ± 12.4 | 13.9 ± 7.0 | |
| Median and [25%; 75%] | 16.6 [12.3; 20.4] | 13.9 [8.2; 17.9] | |
| **Omega_63_relat** | | | 0.691 |
| Mean ± Standard deviation | 15.7 ± 4.8 | 17.6 ± 7.0 | |
| Median and [25%; 75%] | 15.4 [12.6; 18.1] | 15.2 [12.4; 22.5] | |
| **PUFASFA_relat** | | | 0.038 |
| Mean ± Standard deviation | 1.0 ± 0.1 | 1.0 ± 0.1 | |
| Median and [25%; 75%] | 1.0 [1.0; 1.1] | 1.0 [0.9; 1.0] | |
| **TFA_index_relat** | | | 0.862 |
| Mean ± Standard deviation | 0.0 ± 0.0 | 0.0 ± 0.0 | |
| Median and [25%; 75%] | 0.0 [0.0; 0.0] | 0.0 [0.0; 0.0] | |
| **C18.2n6C20.3n6_relat** | | | 0.955 |
| Mean ± Standard deviation | 21.8 ± 7.9 | 22.0 ± 9.1 | |
| Median and [25%; 75%] | 20.2 [15.7; 26.5] | 20.5 [16.4; 24.0] | |
| **AHexCer 54:10;O3\|AHexCer (O-22:3)32:7;O3__[M+H]__1.99** | | | 0.057 |
| Mean ± Standard deviation | 35,740.4 ± 37,551.4 | 53,031.3 ± 54,722.7 | |
| Median and [25%; 75%] | 18,872.5 [10,014.0; 47,180.8] | 35,939.5 [25,071.8; 52,698.0] | |
| **CAR 18:2__[M+H]__1.8** | | | 0.029 |

| | | | |
|---|---|---|---|
| Mean ± Standard deviation | 115,374.3 ± 241,614.2 | 53,799.9 ± 104,175.0 | |
| Median and [25%; 75%] | 36,133.5 [15,615.0; 77,330.5] | 10,286.4 [1,807.8; 71,960.6] | |
| **CE 18:2__[M+NH4]__11.25** | | | 0.270 |
| Mean ± Standard deviation | 211,747,964.2 ± 47,670,589.2 | 195,870,839.4 ± 48,275,291.0 | |
| Median and [25%; 75%] | 212,342,912.0 [177,134,490.0; 254,574, | 202,797,026.0 [177,257,634.0; 224,531,878.0] | |
| **CE 18:3__[M+NH4]__10.64** | | | 0.260 |
| Mean ± Standard deviation | 10,053,828.8 ± 6,456,818.5 | 11,660,529.7 ± 7,266,980.1 | |
| Median and [25%; 75%] | 8,321,357.0 [5,472,546.8; 13,101,340.1] | 10,651,840.1 [7,993,123.3; 14,368,370.5] | |
| **CE 20:3__[M+NH4]__10.81** | | | 0.262 |
| Mean ± Standard deviation | 7,201,344.1 ± 1,990,876.0 | 6,303,276.4 ± 2,867,314.8 | |
| Median and [25%; 75%] | 7,125,559.5 [5,984,816.1; 8,145,912.0] | 6,452,061.9 [4,302,428.0; 8,762,202.1] | |
| **CE 20:4__[M+NH4]__10.67** | | | 0.406 |
| Mean ± Standard deviation | 126,010,282.9 ± 38,946,581.3 | 113,356,764.5 ± 54,774,278.0 | |
| Median and [25%; 75%] | 124,507,796.0 [102,728,046.1; 146,404, | 104,113,796.0 [89,607,185.0; 161,601,324.0] | |
| **CE 20:5__[M+NH4]__9.96** | | | 0.442 |
| Mean ± Standard deviation | 7,867,237.0 ± 17,939,909.8 | 2,873,869.9 ± 7,124,150.2 | |
| Median and [25%; 75%] | 13,666.5 [2,805.5; 10,237,981.5] | 7,790.1 [3,277.6; 413,372.5] | |
| **CE 22:6__[M+NH4]__10.2** | | | 0.374 |
| Mean ± Standard deviation | 9,903,455.6 ± 11,518,138.7 | 7,090,440.6 ± 10,121,820.6 | |
| Median and [25%; 75%] | 7,696,127.0 [368.5; 16,159,955.5] | 847,224.8 [240.1; 13,359,005.6] | |
| **CL 75:4__[M+NH4]__5.26** | | | 0.132 |
| Mean ± Standard deviation | 1,535,051.9 ± 2,073,911.7 | 2,513,270.7 ± 2,841,084.5 | |
| Median and [25%; 75%] | 723,740.0 [68,375.5; 2,324,810.0] | 1,593,721.1 [512,267.8; 3,617,124.3] | |
| Mean ± Standard deviation | | | 0.131 |
| Mean ± Standard deviation | 25,694.7 ± 60,664.0 | 50,599.1 ± 70,323.8 | |
| Median and [25%; 75%] | 4,155.5 [1,212.6; 22,351.8] | 21,173.1 [3,753.5; 59,035.5] | |
| **Cer 40:1;O2\|Cer 18:1;O2/22:0__[M+H]__7.82** | | | <0.001 |
| Mean ± Standard deviation | 106,332.3 ± 63,435.4 | 269,366.8 ± 232,670.7 | |
| Median and [25%; 75%] | 85,061.5 [58,334.9; 155,465.5] | 190,534.5 [144,841.9; 356,025.1] | |
| **Cer 40:1;O2\|Cer 19:0;O2/21:1__[M+Na]__7.83** | | | 0.050 |
| Mean ± Standard deviation | 143,147.8 ± 87,628.4 | 225,600.1 ± 173,625.4 | |
| Median and [25%; 75%] | 118,914.5 [71,066.4; 184,488.8] | 203,285.3 [119,546.3; 260,504.6] | |
| **Cer 41:8;O2\|Cer 17:2;O2/24:6__[M+H-H2O]__6.86** | | | 0.006 |
| Mean ± Standard deviation | 17,056.5 ± 23,208.8 | 53,009.3 ± 55,569.3 | |
| Median and [25%; 75%] | 5,131.0 [2,673.0; 19,319.6] | 32,543.4 [11,226.6; 90,400.9] | |
| **Cer 42:1;O2\|Cer 18:1;O2/24:0__[M+H]__8.6** | | | <0.001 |

| | | | |
|---|---|---|---|
| Mean ± Standard deviation | 2,645,086.3 ± 914,818.0 | 4,166,126.6 ± 1,841,379.7 | |
| Median and [25%; 75%] | 2,566,870.0 [2,134,683.0; 3,044,938.3] | 3,337,161.3 [3,025,400.8; 5,654,769.6] | |
| **Cer 42:2;O2\|Cer 17:1;O2/25:1__[M+Na]__7.98** | | | 0.913 |
| Mean ± Standard deviation | 377,041.6 ± 176,640.4 | 409,108.3 ± 315,757.9 | |
| Median and [25%; 75%] | 358,802.0 [262,764.5; 471,262.3] | 325,666.9 [242,897.6; 504,363.3] | |
| **Cer 42:2;O2\|Cer 18:2;O2/24:0__[M+H]__7.97** | | | 0.027 |
| Mean ± Standard deviation | 303,819.8 ± 160,852.7 | 450,526.1 ± 282,864.8 | |
| Median and [25%; 75%] | 280,075.5 [212,116.5; 363,184.3] | 365,771.0 [279,698.6; 534,695.6] | |
| **Cer 44:7;O2\|Cer 18:1;O2/26:6__[M+Na]__8.6** | | | 0.166 |
| Mean ± Standard deviation | 64,399.3 ± 37,819.0 | 83,704.5 ± 52,038.1 | |
| Median and [25%; 75%] | 54,900.5 [37,445.9; 87,919.5] | 72,898.6 [48,423.6; 100,994.4] | |
| **DG 13:0__[M+Na]__2.95** | | | 0.014 |
| Mean ± Standard deviation | 110,621.7 ± 57,911.2 | 65,386.6 ± 79,050.5 | |
| Median and [25%; 75%] | 105,860.0 [77,794.0; 141,120.3] | 26,372.9 [726.1; 123,262.5] | |
| **DG 18:1__[M+Na]__2.88** | | | 0.402 |
| Mean ± Standard deviation | 15,073.5 ± 27,371.7 | 6,200.6 ± 7,009.0 | |
| Median and [25%; 75%] | 3,417.0 [778.0; 11,264.9] | 3,698.5 [98.3; 10,507.1] | |
| **DG 26:5__[M+Na]__1.99** | | | 0.037 |
| Mean ± Standard deviation | 7,052,242.9 ± 2,443,788.6 | 8,990,108.0 ± 3,243,499.5 | |
| Median and [25%; 75%] | 6,473,784.0 [5,235,220.5; 9,187,238.3] | 8,602,367.0 [7,432,060.3; 9,523,287.8] | |
| **DG 28:3 OA1__[M+Na]__0.96** | | | 0.034 |
| Mean ± Standard deviation | 2,824,465.6 ± 3,567,058.8 | 2,136,645.0 ± 3,991,369.1 | |
| Median and [25%; 75%] | 1,781,832.5 [477,325.0; 3,897,307.1] | 45,426.0 [0.0; 2,920,730.9] | |
| **DG 28:3 OA2__[M+Na]__0.88** | | | 0.068 |
| Mean ± Standard deviation | 2,596,615.2 ± 3,377,011.7 | 2,186,837.4 ± 3,835,260.7 | |
| Median and [25%; 75%] | 1,726,196.3 [478,541.8; 3,472,654.2] | 22,766.8 [0.0; 2,639,798.2] | |
| **DG 28:3__[M+Na]__2.18** | | | 0.016 |
| Mean ± Standard deviation | 176,759.3 ± 82,716.5 | 250,986.7 ± 101,967.1 | |
| Median and [25%; 75%] | 171,912.3 [119,302.5; 236,872.1] | 265,569.8 [173,935.3; 309,193.0] | |
| **DG 28:6__[M+Na]__2.21** | | | 0.004 |
| Mean ± Standard deviation | 1,673,235.3 ± 604,840.6 | 2,513,199.4 ± 1,316,265.3 | |
| Median and [25%; 75%] | 1,620,267.5 [1,159,868.3; 1,906,205.3] | 2,291,168.4 [1,745,742.8; 2,668,588.9] | |
| **DG 31:0__[M+Na]__6.8** | | | <0.001 |
| Mean ± Standard deviation | 27,044.2 ± 41,399.1 | 82,057.4 ± 92,458.1 | |
| Median and [25%; 75%] | 12,357.0 [3,725.8; 27,993.0] | 45,535.9 [28,187.9; 84,609.5] | |
| **DG 31:1__[M+Na]__6.24** | | | 0.006 |

| | | | |
|---|---|---|---|
| Mean ± Standard deviation | 8,670.7 ± 13,989.6 | 22,189.9 ± 21,748.8 | |
| Median and [25%; 75%] | 2,759.0 [1,218.6; 8,339.8] | 9,812.5 [2,673.4; 40,211.0] | |
| **DG 32:0__[M+Na]__6.68** | | | 0.018 |
| Mean ± Standard deviation | 244,959.5 ± 361,121.6 | 422,058.8 ± 460,374.5 | |
| Median and [25%; 75%] | 67,842.5 [26,528.8; 310,141.0] | 192,848.6 [108,328.9; 652,997.3] | |
| **DG 32:0\|DG 16:0_16:0__[M+NH4]__6.68** | | | <0.001 |
| Mean ± Standard deviation | 353,388.7 ± 496,289.8 | 1,133,830.5 ± 1,089,925.9 | |
| Median and [25%; 75%] | 117,117.3 [46,036.4; 414,687.5] | 700,426.3 [420,321.3; 1,670,262.5] | |
| **DG 32:1\|DG 16:0_16:1__[M+NH4]__6.16** | | | <0.001 |
| Mean ± Standard deviation | 136,959.2 ± 264,058.3 | 992,368.3 ± 1,333,088.1 | |
| Median and [25%; 75%] | 24,200.0 [6,805.9; 97,887.3] | 482,218.8 [130,373.9; 1,320,206.8] | |
| **DG 33:0__[M+Na]__7.48** | | | <0.001 |
| Mean ± Standard deviation | 1,623.3 ± 2,426.4 | 10,714.8 ± 14,233.4 | |
| Median and [25%; 75%] | 605.0 [400.5; 1,703.8] | 5,255.3 [1,439.6; 13,293.5] | |
| **DG 33:1__[M+Na]__4.98** | | | 0.001 |
| Mean ± Standard deviation | 4,914.2 ± 3,576.2 | 10,517.5 ± 7,455.7 | |
| Median and [25%; 75%] | 3,897.0 [2,150.6; 6,890.3] | 8,811.5 [5,575.0; 13,068.8] | |
| **DG 33:3__[M+Na]__5.88** | | | 0.577 |
| Mean ± Standard deviation | 5,994.7 ± 10,413.9 | 11,002.8 ± 26,388.9 | |
| Median and [25%; 75%] | 937.5 [131.0; 4,587.0] | 844.0 [0.0; 3,934.5] | |
| **DG 34:0 OA4__[M+Na]__6.36** | | | 0.005 |
| Mean ± Standard deviation | 32,935.9 ± 60,109.6 | 115,081.2 ± 223,044.8 | |
| Median and [25%; 75%] | 7,436.0 [3,550.6; 38,717.0] | 29,805.0 [13,113.9; 84,162.3] | |
| **DG 34:1 OA1__[M+Na]__6.77** | | | 0.066 |
| Mean ± Standard deviation | 2,521,376.3 ± 2,513,557.6 | 3,593,761.4 ± 2,430,643.2 | |
| Median and [25%; 75%] | 1,345,436.8 [756,344.8; 3,739,911.0] | 2,575,630.1 [2,140,250.5; 5,897,521.7] | |
| **DG 34:1 OA2__[M+Na]__6.79** | | | 0.066 |
| Mean ± Standard deviation | 2,529,169.9 ± 2,507,106.6 | 3,593,741.3 ± 2,430,653.0 | |
| Median and [25%; 75%] | 1,345,157.0 [757,880.3; 3,739,757.3] | 2,575,469.0 [2,140,250.5; 5,897,521.7] | |
| **DG 34:1 OA3__[M+Na]__5.89** | | | 0.926 |
| Mean ± Standard deviation | 498,199.3 ± 904,883.2 | 1,020,133.4 ± 2,886,737.5 | |
| Median and [25%; 75%] | 68,657.0 [9,579.9; 521,899.1] | 59,253.3 [12,416.8; 210,558.6] | |
| **DG 34:1\|DG 16:0_18:1__[M+NH4]__6.79** | | | <0.001 |
| Mean ± Standard deviation | 7,073,736.8 ± 7,921,623.1 | 16,954,286.8 ± 12,662,816.1 | |
| Median and [25%; 75%] | 3,914,849.0 [1,595,636.5; 8,513,462.5] | 13,322,309.8 [7,560,773.4; 21,994,317.5] | |
| **DG 34:2__[M+Na]__6.29** | | | 0.899 |

| | | | |
|---|---|---|---|
| Mean ± Standard deviation | 393,901.7 ± 677,997.9 | 232,469.1 ± 320,368.1 | |
| Median and [25%; 75%] | 51,027.5 [9,861.5; 493,231.4] | 64,558.0 [15,749.0; 317,756.0] | |
| **DG 34:2\|DG 16:0_18:2__[M+NH4]__6.28** | | | 0.016 |
| Mean ± Standard deviation | 4,123,876.2 ± 5,904,829.0 | 9,052,018.4 ± 9,339,872.4 | |
| Median and [25%; 75%] | 1,835,372.8 [468,258.5; 5,553,046.3] | 6,332,455.0 [2,132,011.1; 13,433,427.4] | |
| **DG 36:1__[M+Na]__7.48** | | | <0.001 |
| Mean ± Standard deviation | 265,741.0 ± 388,900.2 | 906,305.1 ± 1,012,889.8 | |
| Median and [25%; 75%] | 87,120.0 [30,124.0; 260,826.5] | 552,595.3 [151,819.1; 1,219,500.4] | |
| **DG 36:1\|DG 18:0_18:1__[M+NH4]__7.49** | | | <0.001 |
| Mean ± Standard deviation | 250,736.5 ± 376,602.5 | 1,447,462.5 ± 1,585,094.1 | |
| Median and [25%; 75%] | 80,538.0 [23,183.0; 288,125.1] | 807,143.3 [185,732.5; 2,161,835.6] | |
| **DG 36:2__[M+Na]__6.85** | | | 0.422 |
| Mean ± Standard deviation | 673,094.7 ± 765,264.4 | 706,976.8 ± 635,011.5 | |
| Median and [25%; 75%] | 319,495.5 [173,356.8; 930,806.4] | 452,122.8 [256,676.6; 997,428.3] | |
| **DG 36:2\|DG 18:1_18:1__[M+NH4]__6.86** | | | 0.002 |
| Mean ± Standard deviation | 3,476,318.3 ± 4,139,442.9 | 8,968,041.0 ± 8,122,973.2 | |
| Median and [25%; 75%] | 1,779,800.5 [731,178.1; 4,456,517.6] | 6,178,164.5 [3,051,019.6; 12,932,142.6] | |
| **DG 36:3__[M+Na]__6.35** | | | 0.755 |
| Mean ± Standard deviation | 183,528.7 ± 295,827.2 | 113,213.8 ± 178,691.0 | |
| Median and [25%; 75%] | 29,548.0 [9,781.0; 271,676.3] | 37,992.8 [19,493.5; 90,262.1] | |
| **DG 36:3\|DG 18:1_18:2__[M+NH4]__6.36** | | | 0.025 |
| Mean ± Standard deviation | 5,731,712.9 ± 6,861,969.0 | 12,685,855.5 ± 16,196,278.4 | |
| Median and [25%; 75%] | 3,333,777.8 [884,852.8; 7,209,351.6] | 7,366,340.9 [3,355,724.4; 15,883,050.3] | |
| **DG 36:4__[M+Na]__5.9** | | | 0.899 |
| Mean ± Standard deviation | 62,550.6 ± 137,789.3 | 81,586.8 ± 191,166.5 | |
| Median and [25%; 75%] | 8,659.0 [2,683.4; 29,935.6] | 12,496.1 [1,800.6; 34,026.1] | |
| **DG 38:3__[M+Na]__10.65** | | | 0.188 |
| Mean ± Standard deviation | 434,159.7 ± 487,007.8 | 264,970.8 ± 365,720.8 | |
| Median and [25%; 75%] | 162,814.8 [72,657.5; 783,701.3] | 120,880.4 [54,412.8; 324,713.8] | |
| **DG 38:5\|DG 18:1_20:4__[M+NH4]__6.24** | | | 0.980 |
| Mean ± Standard deviation | 177,146.2 ± 290,708.4 | 123,916.6 ± 118,920.3 | |
| Median and [25%; 75%] | 57,070.0 [29,650.3; 146,117.3] | 116,567.5 [24,071.9; 200,280.3] | |
| **DG 38:7__[M+Na]__7.48** | | | <0.001 |
| Mean ± Standard deviation | 8,231.8 ± 12,392.5 | 32,862.2 ± 40,558.5 | |
| Median and [25%; 75%] | 3,486.0 [1,513.8; 8,111.3] | 15,450.3 [5,494.5; 45,600.0] | |
| **DG 39:1__[M+Na]__11.4** | | | 0.100 |

| | | | |
|---|---|---|---|
| Mean ± Standard deviation | 12,197.9 ± 9,100.0 | 8,109.3 ± 5,053.6 | |
| Median and [25%; 75%] | 9,489.5 [6,381.9; 12,406.0] | 7,062.3 [4,751.7; 10,155.3] | |
| **DG 40:4__[M+Na]__10.6** | | | 0.232 |
| Mean ± Standard deviation | 2,787,231.7 ± 1,465,981.9 | 2,342,799.9 ± 1,155,358.7 | |
| Median and [25%; 75%] | 2,793,084.5 [1,700,269.1; 3,926,140.6] | 2,227,951.8 [1,599,347.3; 3,248,011.6] | |
| **DG 40:5__[M+Na]__10.02** | | | 0.172 |
| Mean ± Standard deviation | 1,395,407.4 ± 901,691.7 | 1,068,770.2 ± 950,176.6 | |
| Median and [25%; 75%] | 1,360,442.0 [723,265.5; 2,038,230.0] | 927,060.1 [407,766.6; 1,486,648.1] | |
| **DG 41:3__[M+Na]__10.88** | | | 0.072 |
| Mean ± Standard deviation | 30,329.2 ± 22,707.2 | 18,566.6 ± 9,551.9 | |
| Median and [25%; 75%] | 22,972.8 [16,302.6; 35,162.8] | 18,444.0 [10,048.6; 25,450.6] | |
| **DG 41:4__[M+Na]__10.14** | | | 0.011 |
| Mean ± Standard deviation | 145,841.1 ± 126,271.9 | 61,975.8 ± 39,213.6 | |
| Median and [25%; 75%] | 106,763.5 [59,832.0; 189,037.5] | 70,964.0 [31,210.1; 82,623.4] | |
| **DG 42:1__[M+Na]__5.17** | | | 0.212 |
| Mean ± Standard deviation | 825,923.6 ± 717,669.1 | 913,361.7 ± 493,939.5 | |
| Median and [25%; 75%] | 639,120.5 [324,983.0; 954,171.0] | 829,124.0 [579,732.1; 1,161,146.3] | |
| **DG 42:6__[M+Na]__10.19** | | | <0.001 |
| Mean ± Standard deviation | 1,298,982.7 ± 742,919.7 | 625,960.0 ± 364,781.6 | |
| Median and [25%; 75%] | 1,131,653.8 [914,640.0; 1,734,584.9] | 760,778.3 [283,892.3; 866,841.1] | |
| **DG 43:0__[M+Na]__6.01** | | | 0.003 |
| Mean ± Standard deviation | 21,794.7 ± 16,815.4 | 36,503.2 ± 20,647.2 | |
| Median and [25%; 75%] | 15,146.0 [9,310.5; 25,676.5] | 34,292.0 [19,282.4; 44,024.1] | |
| **DG 43:5__[M+Na]__10.33** | | | <0.001 |
| Mean ± Standard deviation | 141,612.4 ± 138,202.9 | 38,258.5 ± 46,710.7 | |
| Median and [25%; 75%] | 102,040.0 [43,896.1; 191,837.8] | 18,880.3 [9,772.4; 45,008.7] | |
| **DG 43:7__[M+Na]__5.63** | | | 0.156 |
| Mean ± Standard deviation | 333,556.0 ± 204,927.4 | 417,904.7 ± 219,138.0 | |
| Median and [25%; 75%] | 287,078.0 [197,059.9; 453,700.3] | 369,999.0 [277,623.1; 572,429.5] | |
| **DG 44:3__[M+Na]__5.39** | | | <0.001 |
| Mean ± Standard deviation | 2,364,393.4 ± 945,650.3 | 1,446,474.8 ± 328,203.5 | |
| Median and [25%; 75%] | 2,120,271.5 [1,720,384.4; 2,819,778.3] | 1,516,540.6 [1,175,015.6; 1,701,303.3] | |
| **DG 44:7__[M+Na]__6.02** | | | 0.002 |
| Mean ± Standard deviation | 813,934.2 ± 771,353.5 | 1,842,150.5 ± 1,642,656.3 | |
| Median and [25%; 75%] | 600,289.0 [409,069.5; 993,768.1] | 1,225,658.3 [779,720.4; 2,174,484.6] | |
| **DG 44:8__[M+Na]__5.52** | | | 0.003 |

| | | | |
|---|---|---|---|
| Mean ± Standard deviation | 1,644,815.7 ± 966,471.7 | 3,855,216.2 ± 3,556,040.6 | |
| Median and [25%; 75%] | 1,452,661.3 [909,143.1; 2,054,272.6] | 2,210,168.3 [1,684,908.9; 4,817,279.0] | |
| **DG 45:8__[M+Na]__8.74** | | | <0.001 |
| Mean ± Standard deviation | 4,929,913.7 ± 2,555,199.6 | 10,208,644.8 ± 5,131,488.5 | |
| Median and [25%; 75%] | 4,822,925.8 [3,040,540.1; 6,184,767.8] | 8,928,062.5 [7,728,493.9; 12,352,298.4] | |
| **DG 46:0__[M+Na]__6.96** | | | 0.001 |
| Mean ± Standard deviation | 7,610,408.0 ± 5,177,980.2 | 11,778,405.6 ± 6,417,000.1 | |
| Median and [25%; 75%] | 6,110,469.5 [4,711,094.3; 8,029,842.5] | 9,900,176.0 [7,726,101.3; 14,979,912.5] | |
| **DG 46:6__[M+Na]__5.93** | | | <0.001 |
| Mean ± Standard deviation | 848,310.6 ± 415,710.1 | 1,743,878.0 ± 915,749.4 | |
| Median and [25%; 75%] | 866,409.0 [609,823.3; 1,064,354.0] | 1,720,575.3 [1,027,166.0; 1,944,697.1] | |
| **DG 46:9__[M+Na]__4.98** | | | >0.999 |
| Mean ± Standard deviation | 495,920.4 ± 477,189.2 | 489,547.8 ± 399,005.2 | |
| Median and [25%; 75%] | 314,518.0 [170,947.8; 667,639.6] | 308,974.8 [151,137.4; 791,343.2] | |
| **DG 47:0__[M+Na]__7.34** | | | <0.001 |
| Mean ± Standard deviation | 3,460,097.3 ± 2,220,854.3 | 5,729,745.7 ± 2,442,226.0 | |
| Median and [25%; 75%] | 2,982,871.0 [2,021,202.0; 3,917,939.5] | 5,592,478.8 [3,778,164.3; 7,584,378.5] | |
| **DG 48:0__[M+Na]__7.72** | | | 0.003 |
| Mean ± Standard deviation | 7,047,858.1 ± 4,110,090.2 | 10,234,474.1 ± 4,903,197.7 | |
| Median and [25%; 75%] | 5,891,367.5 [4,338,599.3; 8,997,501.5] | 7,783,057.3 [7,270,983.5; 12,408,960.1] | |
| **DG 48:1 OA1__[M+Na]__6.91** | | | 0.025 |
| Mean ± Standard deviation | 31,533,381.1 ± 24,130,330.4 | 36,883,692.1 ± 11,128,628.2 | |
| Median and [25%; 75%] | 23,925,985.0 [16,614,539.0; 38,009,578 | 39,527,859.0 [30,983,378.8; 42,415,624.1] | |
| **DG 48:1__[M+Na]__7.14** | | | <0.001 |
| Mean ± Standard deviation | 8,035,157.7 ± 5,201,167.6 | 11,496,018.1 ± 4,162,723.1 | |
| Median and [25%; 75%] | 6,102,565.0 [4,850,730.3; 9,179,904.5] | 11,721,004.3 [8,196,746.0; 14,133,895.1] | |
| **DG 48:2__[M+Na]__6.38** | | | 0.560 |
| Mean ± Standard deviation | 18,065,266.2 ± 10,191,517.5 | 17,085,845.8 ± 4,679,960.7 | |
| Median and [25%; 75%] | 15,720,322.0 [11,645,270.0; 20,573,925 | 16,661,072.3 [14,384,642.3; 21,041,896.4] | |
| **DG 49:0__[M+Na]__11.52** | | | <0.001 |
| Mean ± Standard deviation | 211,975.9 ± 95,699.1 | 116,189.9 ± 85,676.8 | |
| Median and [25%; 75%] | 210,394.0 [148,842.5; 277,949.8] | 70,159.3 [63,459.8; 156,588.9] | |
| **DG 49:12__[M+Na]__4.72** | | | 0.009 |
| Mean ± Standard deviation | 215,868.2 ± 296,795.4 | 419,384.6 ± 363,406.0 | |
| Median and [25%; 75%] | 143,460.8 [64,578.1; 229,412.8] | 276,904.3 [121,220.5; 609,699.1] | |
| **DG 49:7__[M+Na]__5.94** | | | 0.002 |

| | | | |
|---|---|---|---|
| Mean ± Standard deviation | 128,277.4 ± 104,187.5 | 200,073.1 ± 108,369.4 | |
| Median and [25%; 75%] | 103,276.5 [60,367.4; 142,621.3] | 169,478.1 [115,938.1; 237,159.3] | |
| **DG 49:8__[M+Na]__7.11** | | | <0.001 |
| Mean ± Standard deviation | 400,058.9 ± 330,831.7 | 879,052.0 ± 385,035.0 | |
| Median and [25%; 75%] | 355,622.0 [94,917.5; 622,749.5] | 822,216.3 [571,872.4; 1,080,307.6] | |
| **DG 49:9__[M+Na]__5.37** | | | 0.886 |
| Mean ± Standard deviation | 160,885,241.0 ± 62,669,081.6 | 162,409,542.4 ± 78,219,876.6 | |
| Median and [25%; 75%] | 144,569,848.0 [109,364,624.0; 193,826, | 147,250,146.0 [110,806,135.0; 239,152,374.0] | |
| **DG 50:5__[M+Na]__6.36** | | | 0.020 |
| Mean ± Standard deviation | 88,267.1 ± 65,870.4 | 46,025.2 ± 22,585.2 | |
| Median and [25%; 75%] | 70,091.0 [39,466.5; 119,550.6] | 42,944.3 [29,506.3; 67,828.7] | |
| **DG 51:11__[M+Na]__5.93** | | | 0.128 |
| Mean ± Standard deviation | 50,247,106.1 ± 23,085,917.5 | 65,824,255.1 ± 33,404,415.7 | |
| Median and [25%; 75%] | 42,487,614.0 [30,879,719.5; 65,423,978 | 65,531,223.5 [50,294,632.0; 79,346,761.5] | |
| **DG 51:3__[M+Na]__10.9** | | | 0.064 |
| Mean ± Standard deviation | 154,423.4 ± 140,251.7 | 212,515.5 ± 115,284.1 | |
| Median and [25%; 75%] | 152,810.8 [1,231.3; 230,732.5] | 203,892.3 [155,623.3; 252,403.1] | |
| **DG 51:5__[M+Na]__11.04** | | | <0.001 |
| Mean ± Standard deviation | 1,125,643.4 ± 1,058,271.5 | 3,688,329.0 ± 1,886,192.6 | |
| Median and [25%; 75%] | 935,002.8 [344,373.5; 1,524,869.8] | 3,791,007.3 [2,619,467.1; 5,123,521.1] | |
| **DG 51:6__[M+Na]__10.4** | | | <0.001 |
| Mean ± Standard deviation | 217,375.7 ± 254,762.6 | 798,338.1 ± 381,436.1 | |
| Median and [25%; 75%] | 170,667.0 [24,312.8; 316,196.5] | 920,963.8 [479,615.1; 1,023,756.5] | |
| **DG 52:10__[M+Na]__6.83** | | | 0.023 |
| Mean ± Standard deviation | 909,394.9 ± 368,375.3 | 1,232,470.2 ± 573,532.3 | |
| Median and [25%; 75%] | 870,474.5 [651,437.9; 1,124,261.5] | 1,163,066.8 [928,572.9; 1,428,120.1] | |
| **DG O-35:1\|DG O-17:0_18:1__[M+NH4]__6.8** | | | 0.004 |
| Mean ± Standard deviation | 935,884.1 ± 1,426,717.4 | 1,869,885.8 ± 1,563,221.1 | |
| Median and [25%; 75%] | 304,029.0 [96,840.6; 1,376,435.8] | 1,272,163.3 [614,561.4; 2,979,479.0] | |
| **DG O-37:1\|DG O-17:1_20:0__[M+NH4]__11.22** | | | 0.545 |
| Mean ± Standard deviation | 160,744.0 ± 318,643.4 | 74,829.6 ± 93,677.2 | |
| Median and [25%; 75%] | 31,438.5 [1,945.8; 138,914.4] | 37,812.6 [25,555.4; 88,879.5] | |
| **DG O-37:2\|DG O-19:1_18:1__[M+NH4]__6.86** | | | 0.021 |
| Mean ± Standard deviation | 362,778.8 ± 613,909.3 | 757,403.7 ± 833,632.6 | |
| Median and [25%; 75%] | 116,628.8 [38,503.9; 377,315.5] | 469,720.3 [182,605.1; 1,074,267.1] | |
| **DG O-37:4\|DG O-19:2_18:2__[M+NH4]__5.9** | | | 0.072 |

| | | | |
|---|---|---|---|
| Mean ± Standard deviation | 101,296.6 ± 252,494.9 | 1,191,665.7 ± 4,197,965.6 | |
| Median and [25%; 75%] | 16,242.5 [5,301.5; 84,013.5] | 54,113.6 [14,125.3; 137,789.5] | |
| **DG O-39:3\|DG O-19:3_20:0__[M+NH4]__10.63** | | | 0.642 |
| Mean ± Standard deviation | 5,378,371.3 ± 4,079,835.0 | 5,756,086.0 ± 4,938,571.0 | |
| Median and [25%; 75%] | 4,457,293.3 [2,098,031.5; 7,024,504.1] | 5,318,557.5 [3,887,564.0; 6,687,507.1] | |
| **DG O-41:3\|DG O-21:3_20:0__[M+NH4]__11.47** | | | 0.090 |
| Mean ± Standard deviation | 2,736,621.7 ± 1,654,604.5 | 1,918,699.5 ± 909,068.7 | |
| Median and [25%; 75%] | 2,538,805.0 [1,430,978.4; 3,298,605.8] | 1,865,277.3 [1,380,691.3; 2,602,686.3] | |
| **LPC 14:0/0:0__[M+H]__1.46** | | | <0.001 |
| Mean ± Standard deviation | 1,081,585.0 ± 612,692.6 | 2,062,493.8 ± 697,855.4 | |
| Median and [25%; 75%] | 875,362.5 [637,478.9; 1,386,582.5] | 2,026,085.8 [1,644,410.0; 2,443,340.4] | |
| **LPC 15:0/0:0__[M+H]__1.7** | | | 0.010 |
| Mean ± Standard deviation | 755,192.2 ± 453,123.6 | 1,059,643.5 ± 407,312.0 | |
| Median and [25%; 75%] | 706,843.0 [397,644.8; 961,553.3] | 1,082,213.8 [668,191.6; 1,422,922.8] | |
| **LPC 16:0__[M+H]__2.0** | | | 0.059 |
| Mean ± Standard deviation | 168,848,371.5 ± 48,364,196.2 | 193,579,205.0 ± 44,472,647.5 | |
| Median and [25%; 75%] | 170,663,232.0 [131,375,274.0; 197,243, | 183,946,644.0 [174,631,612.0; 209,432,362.0] | |
| **LPC 16:0__[M+Na]__2.0** | | | 0.339 |
| Mean ± Standard deviation | 5,971,073.8 ± 1,145,347.2 | 6,240,247.0 ± 864,580.6 | |
| Median and [25%; 75%] | 5,880,358.5 [5,062,908.5; 6,781,772.6] | 6,229,864.3 [5,849,048.1; 6,564,592.5] | |
| **LPC 16:1/0:0__[M+H]__1.61** | | | 0.078 |
| Mean ± Standard deviation | 4,049,385.7 ± 1,844,160.9 | 5,092,375.6 ± 2,662,068.2 | |
| Median and [25%; 75%] | 3,363,072.0 [2,570,778.1; 4,609,750.8] | 4,348,778.6 [3,719,557.4; 5,897,038.6] | |
| **LPC 17:0/0:0__[M+H]__2.33** | | | 0.013 |
| Mean ± Standard deviation | 3,372,429.2 ± 1,321,377.5 | 4,332,653.4 ± 1,211,682.0 | |
| Median and [25%; 75%] | 3,152,134.3 [2,450,612.8; 4,282,060.8] | 4,352,474.8 [3,340,465.5; 5,057,298.5] | |
| **LPC 18:0__[M+H]__2.56** | | | 0.001 |
| Mean ± Standard deviation | 69,949,503.6 ± 21,128,295.5 | 92,124,697.6 ± 25,059,138.6 | |
| Median and [25%; 75%] | 71,625,120.0 [54,377,323.0; 85,600,176 | 87,431,126.0 [74,775,834.0; 103,602,068.0] | |
| **LPC 18:0__[M+Na]__2.56** | | | 0.239 |
| Mean ± Standard deviation | 5,857,406.0 ± 1,715,465.7 | 5,481,742.8 ± 1,954,553.8 | |
| Median and [25%; 75%] | 5,243,924.0 [4,712,976.0; 7,049,481.5] | 4,901,568.3 [4,000,660.0; 5,862,464.0] | |
| **LPC 18:1/0:0__[M+H]__2.18** | | | 0.298 |
| Mean ± Standard deviation | 36,858,028.7 ± 11,000,354.3 | 45,927,970.8 ± 24,321,809.8 | |
| Median and [25%; 75%] | 35,206,581.0 [29,730,256.5; 42,949,786 | 37,652,521.5 [31,148,625.0; 57,245,720.0] | |
| **LPC 18:1__[M+Na]__2.2** | | | <0.001 |

| | | | |
|---|---|---|---|
| Mean ± Standard deviation | 2,922,737.1 ± 1,194,825.5 | 3,943,104.3 ± 691,881.1 | |
| Median and [25%; 75%] | 2,814,707.3 [2,329,174.3; 3,602,079.5] | 3,800,937.8 [3,480,429.3; 4,379,731.8] | |
| **LPC 18:2/0:0__[M+H]__1.78** | | | 0.010 |
| Mean ± Standard deviation | 53,626,938.0 ± 24,106,153.9 | 76,570,400.5 ± 38,678,207.2 | |
| Median and [25%; 75%] | 46,316,780.0 [38,645,070.5; 60,601,226 | 69,354,863.0 [47,639,766.0; 93,556,380.0] | |
| **LPC 18:2__[M+H]__1.77** | | | 0.009 |
| Mean ± Standard deviation | 52,428,626.2 ± 22,683,841.9 | 75,412,656.3 ± 36,526,018.4 | |
| Median and [25%; 75%] | 45,376,790.0 [37,273,648.5; 60,223,101 | 69,603,866.0 [48,636,608.8; 92,721,132.0] | |
| **LPC 18:2__[M+Na]__1.77** | | | 0.044 |
| Mean ± Standard deviation | 2,831,662.0 ± 781,422.4 | 3,529,625.6 ± 1,131,135.6 | |
| Median and [25%; 75%] | 2,696,551.5 [2,321,498.3; 3,169,268.8] | 3,134,354.8 [2,485,974.8; 4,563,887.1] | |
| **LPC 18:3/0:0__[M+H]__1.51** | | | 0.006 |
| Mean ± Standard deviation | 187,472.6 ± 161,800.8 | 384,616.8 ± 321,186.2 | |
| Median and [25%; 75%] | 137,079.0 [83,777.9; 265,167.5] | 285,384.3 [155,297.9; 438,724.4] | |
| **LPC 20:1/0:0__[M+H]__2.92** | | | 0.833 |
| Mean ± Standard deviation | 493,510.9 ± 292,635.6 | 508,176.4 ± 307,102.8 | |
| Median and [25%; 75%] | 367,350.5 [284,724.1; 646,309.3] | 378,147.5 [293,379.4; 623,768.1] | |
| **LPC 20:2/0:0__[M+H]__2.38** | | | 0.820 |
| Mean ± Standard deviation | 637,554.3 ± 432,146.0 | 606,989.1 ± 377,264.4 | |
| Median and [25%; 75%] | 513,620.8 [357,166.8; 714,558.1] | 503,525.3 [369,757.3; 712,678.4] | |
| **LPC 20:4/0:0__[M+H]__1.81** | | | 0.704 |
| Mean ± Standard deviation | 18,054,607.1 ± 13,046,819.4 | 14,177,439.8 ± 5,761,277.7 | |
| Median and [25%; 75%] | 14,156,673.3 [9,902,012.5; 19,889,408.5 | 14,001,472.0 [11,603,009.6; 18,869,676.3] | |
| **LPC 20:4__[M+H]__1.71** | | | 0.583 |
| Mean ± Standard deviation | 13,363,462.7 ± 10,844,261.4 | 10,867,054.3 ± 6,792,887.9 | |
| Median and [25%; 75%] | 10,641,900.0 [6,385,481.6; 15,708,569.9 | 10,169,912.3 [4,919,587.0; 18,452,913.3] | |
| **LPC 20:4__[M+Na]__1.71** | | | 0.132 |
| Mean ± Standard deviation | 488,575.6 ± 470,956.4 | 317,801.9 ± 271,619.7 | |
| Median and [25%; 75%] | 296,992.5 [212,524.6; 661,887.3] | 178,516.0 [124,437.4; 543,179.3] | |
| **LPC 20:5/0:0__[M+H]__1.51** | | | 0.282 |
| Mean ± Standard deviation | 1,130,746.6 ± 1,215,851.1 | 1,499,946.9 ± 1,670,674.9 | |
| Median and [25%; 75%] | 758,229.5 [365,899.8; 1,485,578.3] | 798,745.3 [541,605.9; 1,791,969.4] | |
| **LPC 20:5__[M+Na]__1.51** | | | 0.820 |
| Mean ± Standard deviation | 108,803.6 ± 141,885.5 | 128,204.3 ± 180,638.2 | |
| Median and [25%; 75%] | 53,191.5 [21,490.3; 131,766.5] | 52,604.6 [28,165.5; 183,761.0] | |
| **LPC 22:4__[M+H]__2.3** | | | 0.926 |

| | | | |
|---|---|---|---|
| Mean ± Standard deviation | 132,928.6 ± 138,537.2 | 119,161.4 ± 98,296.1 | |
| Median and [25%; 75%] | 89,718.3 [36,979.6; 193,881.4] | 80,889.8 [51,390.9; 175,035.0] | |
| **LPC 22:5__[M+H]__1.91** | | | 0.704 |
| Mean ± Standard deviation | 238,390.6 ± 284,655.2 | 137,050.6 ± 139,986.9 | |
| Median and [25%; 75%] | 86,366.5 [40,983.1; 364,568.0] | 83,958.3 [43,468.9; 176,931.1] | |
| **LPC 22:6/0:0__[M+H]__1.73** | | | 0.052 |
| Mean ± Standard deviation | 5,735,115.4 ± 3,595,643.1 | 3,774,726.4 ± 2,174,730.6 | |
| Median and [25%; 75%] | 5,027,549.0 [3,443,991.3; 7,235,275.8] | 3,137,453.4 [2,194,979.1; 4,861,692.3] | |
| **LPC 22:6__[M+H]__1.64** | | | 0.537 |
| Mean ± Standard deviation | 4,647,966.7 ± 3,346,630.9 | 3,728,110.9 ± 2,229,868.4 | |
| Median and [25%; 75%] | 3,894,482.5 [2,289,051.6; 6,325,546.3] | 3,137,453.4 [2,192,514.8; 4,861,692.3] | |
| **LPC 22:6__[M+Na]__1.64** | | | 0.183 |
| Mean ± Standard deviation | 285,784.3 ± 217,671.6 | 199,620.2 ± 148,476.9 | |
| Median and [25%; 75%] | 251,453.0 [132,164.5; 405,079.3] | 146,177.8 [104,115.4; 270,793.3] | |
| **LPC O-16:0__[M+H]__2.25** | | | 0.055 |
| Mean ± Standard deviation | 346,303.9 ± 305,459.6 | 460,868.8 ± 263,614.1 | |
| Median and [25%; 75%] | 221,140.3 [132,985.9; 478,040.8] | 392,334.4 [284,035.5; 594,031.1] | |
| **LPC O-16:1__[M+H]__2.25** | | | 0.055 |
| Mean ± Standard deviation | 523,674.8 ± 373,499.2 | 761,405.4 ± 472,623.5 | |
| Median and [25%; 75%] | 397,960.0 [241,989.3; 719,432.3] | 666,989.4 [444,930.5; 975,327.1] | |
| **LPC O-18:0__[M+H]__3.04** | | | 0.225 |
| Mean ± Standard deviation | 378,305.9 ± 214,655.4 | 458,253.0 ± 259,506.9 | |
| Median and [25%; 75%] | 306,244.0 [243,715.0; 460,272.3] | 374,190.0 [261,604.8; 569,452.4] | |
| **LPC O-18:1__[M+H]__2.44** | | | 0.183 |
| Mean ± Standard deviation | 1,088,139.1 ± 627,888.5 | 839,187.5 ± 460,287.5 | |
| Median and [25%; 75%] | 876,645.3 [610,897.0; 1,422,840.3] | 682,022.1 [570,761.9; 902,325.3] | |
| **LPC O-19:0 OA1__[M+H]__2.57** | | | 0.151 |
| Mean ± Standard deviation | 471,282.8 ± 295,997.1 | 504,156.4 ± 170,258.1 | |
| Median and [25%; 75%] | 403,629.0 [292,725.0; 556,219.3] | 475,501.5 [358,432.8; 635,766.8] | |
| **LPC O-19:0__[M+H]__2.73** | | | 0.006 |
| Mean ± Standard deviation | 3,777,720.2 ± 2,855,808.6 | 5,236,553.8 ± 2,336,542.9 | |
| Median and [25%; 75%] | 3,510,795.0 [2,043,724.9; 4,198,416.3] | 4,313,642.3 [3,804,415.3; 6,847,295.5] | |
| **LPC O-24:0__[M+H]__4.57** | | | 0.008 |
| Mean ± Standard deviation | 158,487.8 ± 101,393.2 | 265,555.6 ± 163,244.2 | |
| Median and [25%; 75%] | 149,942.3 [85,511.8; 208,859.3] | 261,283.3 [166,453.4; 299,384.0] | |
| **LPC O-24:1__[M+H]__4.13** | | | 0.033 |

| | | | |
|---|---|---|---|
| Mean ± Standard deviation | 190,117.6 ± 93,815.8 | 263,814.3 ± 151,074.4 | |
| Median and [25%; 75%] | 176,706.0 [105,141.1; 223,412.8] | 248,411.0 [191,824.0; 276,433.6] | |
| **LPE 22:6__[M+H]__1.73** | | | 0.002 |
| Mean ± Standard deviation | 103,277.3 ± 118,735.7 | 39,495.3 ± 52,163.2 | |
| Median and [25%; 75%] | 66,172.0 [36,002.3; 120,297.9] | 19,305.6 [12,114.9; 54,821.3] | |
| **NAE 17:1__[M+H]__3.86** | | | 0.031 |
| Mean ± Standard deviation | 35,559.5 ± 86,958.2 | 63,346.1 ± 93,307.0 | |
| Median and [25%; 75%] | 1,355.0 [235.3; 41,025.8] | 21,828.5 [2,711.1; 96,023.9] | |
| **NAE 19:3__[M+H]__3.79** | | | 0.128 |
| Mean ± Standard deviation | 252,334.1 ± 379,018.2 | 262,424.6 ± 303,691.1 | |
| Median and [25%; 75%] | 47,024.3 [10,675.0; 356,418.0] | 111,322.0 [55,343.0; 353,132.3] | |
| **NAE 22:5__[M+H]__3.37** | | | 0.306 |
| Mean ± Standard deviation | 3,426.7 ± 6,094.8 | 2,325.0 ± 4,162.5 | |
| Median and [25%; 75%] | 1,042.5 [324.8; 3,694.3] | 904.5 [124.9; 1,672.6] | |
| **NAE 22:6__[M+H]__2.88** | | | 0.733 |
| Mean ± Standard deviation | 4,804.1 ± 11,659.6 | 6,830.4 ± 15,151.0 | |
| Median and [25%; 75%] | 906.5 [218.0; 2,437.5] | 896.0 [64.6; 2,859.4] | |
| **NAE 26:7__[M+H]__2.19** | | | 0.132 |
| Mean ± Standard deviation | 26,772.2 ± 46,042.6 | 14,028.8 ± 21,274.0 | |
| Median and [25%; 75%] | 14,802.0 [3,737.0; 30,014.4] | 5,228.1 [2,439.0; 15,338.9] | |
| **PC 17:0__[M+H]__2.73** | | | <0.001 |
| Mean ± Standard deviation | 371,652.9 ± 264,478.7 | 658,187.1 ± 299,947.0 | |
| Median and [25%; 75%] | 293,373.8 [192,639.5; 488,026.3] | 664,645.3 [408,823.0; 777,271.9] | |
| **PC 30:0__[M+H]__5.04** | | | <0.001 |
| Mean ± Standard deviation | 558,157.8 ± 755,123.3 | 2,432,620.9 ± 2,014,875.0 | |
| Median and [25%; 75%] | 320,049.5 [161,462.8; 565,687.3] | 2,001,865.4 [1,012,226.9; 3,379,397.3] | |
| **PC 32:0__[M+H]__5.16** | | | <0.001 |
| Mean ± Standard deviation | 34,195,179.6 ± 8,493,272.5 | 49,696,164.6 ± 11,918,230.6 | |
| Median and [25%; 75%] | 34,343,193.0 [28,815,429.5; 37,890,781 | 48,256,849.0 [41,317,769.5; 59,874,047.0] | |
| **PC 32:0\|PC 16:0_16:0__[M+H]__5.55** | | | <0.001 |
| Mean ± Standard deviation | 34,178,550.7 ± 8,488,830.6 | 49,672,054.1 ± 11,919,366.5 | |
| Median and [25%; 75%] | 34,319,069.5 [28,796,828.0; 37,869,343 | 48,236,569.0 [41,275,820.0; 59,841,582.0] | |
| **PC 32:1\|PC 16:0_16:1__[M+H]__5.16** | | | <0.001 |
| Mean ± Standard deviation | 15,211,223.0 ± 9,716,172.5 | 37,766,491.6 ± 18,490,161.0 | |
| Median and [25%; 75%] | 13,219,797.0 [8,165,024.6; 21,321,431.5 | 33,202,595.5 [25,834,819.5; 43,865,374.5] | |
| **PC 32:2__[M+Na]__4.78** | | | <0.001 |

| | | | |
|---|---|---|---|
| Mean ± Standard deviation | 169,153.4 ± 128,905.9 | 383,472.7 ± 173,602.0 | |
| Median and [25%; 75%] | 161,707.5 [75,623.5; 209,175.0] | 368,344.0 [286,882.3; 439,456.6] | |
| **PC 32:2\|PC 14:0_18:2__[M+H]__4.79** | | | <0.001 |
| Mean ± Standard deviation | 5,683,578.3 ± 3,159,215.7 | 14,974,294.0 ± 6,577,842.0 | |
| Median and [25%; 75%] | 5,272,930.0 [3,551,878.5; 6,835,926.1] | 13,248,996.0 [10,655,184.6; 17,776,517.9] | |
| **PC 33:1__[M+H]__5.6** | | | 0.225 |
| Mean ± Standard deviation | 508,911.7 ± 925,261.6 | 699,709.1 ± 1,971,163.6 | |
| Median and [25%; 75%] | 79,682.5 [50,528.3; 509,742.4] | 143,076.8 [88,317.8; 242,037.6] | |
| **PC 33:1\|PC 15:0_18:1__[M+H]__5.41** | | | <0.001 |
| Mean ± Standard deviation | 1,786,386.1 ± 987,707.8 | 5,045,753.1 ± 2,664,961.6 | |
| Median and [25%; 75%] | 1,535,989.5 [1,079,248.0; 2,210,975.0] | 4,775,592.8 [3,221,437.8; 7,222,608.5] | |
| **PC 33:2\|PC 15:0_18:2__[M+H]__5.01** | | | <0.001 |
| Mean ± Standard deviation | 6,209,869.1 ± 2,704,023.0 | 12,935,364.7 ± 7,038,217.9 | |
| Median and [25%; 75%] | 5,975,477.8 [4,210,589.3; 8,002,101.4] | 10,620,746.6 [9,081,011.9; 17,154,372.9] | |
| **PC 34:0__[M+H]__6.13** | | | <0.001 |
| Mean ± Standard deviation | 8,483,195.4 ± 2,189,600.3 | 14,128,123.5 ± 3,941,865.6 | |
| Median and [25%; 75%] | 8,305,602.5 [6,740,679.3; 9,808,272.5] | 13,650,697.0 [11,121,136.4; 17,620,170.9] | |
| **PC 34:1__[M+Na]__5.66** | | | 0.384 |
| Mean ± Standard deviation | 269,872,707.4 ± 75,250,404.5 | 294,201,949.7 ± 99,420,585.4 | |
| Median and [25%; 75%] | 269,464,720.0 [221,488,339.0; 313,099, | 308,962,056.0 [261,397,502.0; 365,497,188.0] | |
| **PC 34:2__[M+H]__5.0** | | | <0.001 |
| Mean ± Standard deviation | 517,512,900.8 ± 97,358,400.0 | 634,440,293.0 ± 82,018,820.3 | |
| Median and [25%; 75%] | 525,300,352.0 [458,094,916.0; 581,062, | 621,880,368.0 [579,644,080.0; 675,385,236.0] | |
| **PC 34:3\|PC 16:0_18:3__[M+H]__5.01** | | | <0.001 |
| Mean ± Standard deviation | 21,598,254.9 ± 9,132,360.0 | 44,922,051.2 ± 18,939,176.4 | |
| Median and [25%; 75%] | 20,995,317.5 [14,798,722.0; 26,875,647 | 39,928,502.0 [31,501,261.3; 55,074,175.5] | |
| **PC 34:3\|PC 16:1_18:2__[M+H]__4.89** | | | <0.001 |
| Mean ± Standard deviation | 21,627,933.7 ± 9,311,503.3 | 45,221,578.1 ± 18,492,463.9 | |
| Median and [25%; 75%] | 20,996,381.5 [14,698,513.0; 26,676,451 | 39,220,072.5 [33,113,318.0; 56,400,535.5] | |
| **PC 35:1__[M+H]__5.5** | | | <0.001 |
| Mean ± Standard deviation | 692,747.1 ± 292,661.8 | 1,431,158.5 ± 795,410.3 | |
| Median and [25%; 75%] | 662,616.3 [473,822.8; 864,146.5] | 1,284,743.0 [849,881.8; 1,621,228.8] | |
| **PC 35:2__[M+H]__6.17** | | | <0.001 |
| Mean ± Standard deviation | 24,789,214.9 ± 7,786,732.1 | 50,127,096.8 ± 26,000,893.0 | |
| Median and [25%; 75%] | 25,849,869.0 [19,335,744.3; 29,944,355 | 44,458,775.0 [34,374,537.3; 52,769,036.0] | |
| **PC 35:2\|PC 17:0_18:2__[M+H]__5.5** | | | <0.001 |

| | | | |
|---|---|---|---|
| Mean ± Standard deviation | 24,766,740.8 ± 7,784,987.0 | 50,094,617.3 ± 25,992,615.0 | |
| Median and [25%; 75%] | 25,832,555.0 [19,322,067.5; 29,927,018 | 44,428,064.5 [34,349,228.0; 52,739,312.0] | |
| **PC 35:4\|PC 15:0_20:4__[M+H]__4.98** | | | 0.151 |
| Mean ± Standard deviation | 2,806,305.1 ± 1,827,791.5 | 3,854,279.9 ± 2,339,099.0 | |
| Median and [25%; 75%] | 2,219,336.5 [1,620,589.5; 3,603,753.5] | 2,961,084.5 [1,851,013.4; 5,900,533.4] | |
| **PC 36:0\|PC 18:0_18:0__[M+H]__6.25** | | | <0.001 |
| Mean ± Standard deviation | 7,016,832.3 ± 2,335,836.6 | 15,561,623.4 ± 13,390,225.0 | |
| Median and [25%; 75%] | 6,704,849.0 [5,402,026.6; 7,644,986.1] | 12,807,140.5 [8,554,858.4; 16,902,791.6] | |
| **PC 36:1\|PC 18:0_18:1__[M+H]__6.26** | | | <0.001 |
| Mean ± Standard deviation | 120,895,050.2 ± 31,268,724.0 | 215,674,189.3 ± 82,741,079.5 | |
| Median and [25%; 75%] | 115,622,696.0 [101,141,358.0; 137,452, | 193,221,812.0 [156,319,796.0; 256,544,189.0] | |
| **PC 36:2\|PC 18:0_18:2__[M+H]__5.81** | | | <0.001 |
| Mean ± Standard deviation | 424,350,852.5 ± 87,574,813.4 | 575,198,097.0 ± 78,918,679.8 | |
| Median and [25%; 75%] | 434,057,040.0 [371,895,880.0; 478,787, | 559,648,792.0 [535,531,920.0; 591,070,156.0] | |
| **PC 36:3__[M+Na]__5.41** | | | 0.001 |
| Mean ± Standard deviation | 237,617,486.5 ± 45,349,745.9 | 291,321,316.3 ± 51,630,047.0 | |
| Median and [25%; 75%] | 236,759,376.0 [206,543,828.0; 272,326, | 291,142,260.0 [259,006,722.0; 314,354,726.0] | |
| **PC 36:4\|PC 18:2_18:2__[M+H]__4.96** | | | <0.001 |
| Mean ± Standard deviation | 52,259,697.6 ± 29,907,989.8 | 128,969,847.6 ± 70,127,415.2 | |
| Median and [25%; 75%] | 45,210,970.0 [37,062,379.0; 57,763,775 | 111,288,417.0 [73,325,812.0; 151,172,444.0] | |
| **PC 36:5__[M+Na]__4.87** | | | 0.504 |
| Mean ± Standard deviation | 846,660.6 ± 466,515.1 | 777,914.6 ± 514,146.7 | |
| Median and [25%; 75%] | 760,463.0 [505,338.8; 1,116,447.5] | 648,870.0 [430,402.0; 903,967.1] | |
| **PC 36:5\|PC 16:0_20:5__[M+H]__4.88** | | | 0.002 |
| Mean ± Standard deviation | 54,066,524.2 ± 41,991,193.7 | 99,663,389.0 ± 59,785,346.9 | |
| Median and [25%; 75%] | 50,392,778.0 [26,626,548.5; 61,289,982 | 81,099,052.0 [53,286,576.0; 138,388,511.0] | |
| **PC 36:5\|PC 18:2_18:3__[M+H]__4.64** | | | 0.003 |
| Mean ± Standard deviation | 54,377,750.7 ± 41,900,132.6 | 99,533,696.6 ± 59,712,132.0 | |
| Median and [25%; 75%] | 50,917,118.0 [26,762,684.0; 61,634,409 | 80,980,660.0 [53,063,189.8; 138,132,066.0] | |
| **PC 36:6__[M+H]__4.53** | | | <0.001 |
| Mean ± Standard deviation | 725,867.5 ± 986,101.2 | 1,510,011.3 ± 1,278,714.9 | |
| Median and [25%; 75%] | 391,265.5 [132,453.3; 832,641.5] | 1,018,092.0 [765,028.9; 1,753,603.9] | |
| **PC 36:6\|PC 14:0_22:6__[M+H]__4.62** | | | <0.001 |
| Mean ± Standard deviation | 716,424.5 ± 954,245.4 | 1,502,583.5 ± 1,272,932.6 | |
| Median and [25%; 75%] | 390,729.5 [131,961.3; 828,429.9] | 1,013,771.8 [762,254.8; 1,748,621.8] | |
| **PC 37:1__[M+H]__6.56** | | | <0.001 |

| | | | |
|---|---|---|---|
| Mean ± Standard deviation | 181,169.3 ± 80,486.6 | 538,643.2 ± 551,926.1 | |
| Median and [25%; 75%] | 181,561.5 [122,514.3; 226,411.5] | 335,951.0 [293,598.7; 426,764.6] | |
| **PC 37:3__[M+H]__5.63** | | | <0.001 |
| Mean ± Standard deviation | 472,297.4 ± 444,729.5 | 965,951.5 ± 509,338.9 | |
| Median and [25%; 75%] | 310,072.5 [191,673.3; 540,313.8] | 972,506.8 [621,326.5; 1,163,659.9] | |
| **PC 37:4__[M+H]__6.15** | | | 0.080 |
| Mean ± Standard deviation | 12,690,430.6 ± 5,152,342.2 | 16,252,489.6 ± 7,180,875.3 | |
| Median and [25%; 75%] | 11,418,138.0 [9,329,314.5; 15,652,353.0 | 16,404,445.3 [11,058,314.3; 23,575,285.1] | |
| **PC 37:4\|PC 17:0_20:4__[M+H]__5.47** | | | 0.080 |
| Mean ± Standard deviation | 12,600,647.7 ± 5,160,793.7 | 16,179,202.6 ± 7,159,072.7 | |
| Median and [25%; 75%] | 11,359,828.0 [9,091,092.5; 15,601,030.3 | 16,325,009.5 [11,006,787.4; 23,510,101.4] | |
| **PC 37:5__[M+H]__5.63** | | | 0.239 |
| Mean ± Standard deviation | 1,502,230.5 ± 1,201,209.3 | 1,706,022.2 ± 822,454.3 | |
| Median and [25%; 75%] | 1,246,261.0 [681,282.5; 1,938,897.3] | 1,634,159.8 [1,292,233.3; 2,212,782.6] | |
| **PC 37:6__[M+H]__5.35** | | | 0.029 |
| Mean ± Standard deviation | 1,234,035.6 ± 667,014.4 | 1,740,067.0 ± 838,136.5 | |
| Median and [25%; 75%] | 1,185,923.0 [743,754.5; 1,514,269.4] | 1,817,757.8 [1,156,416.0; 2,239,883.2] | |
| **PC 37:6\|PC 15:0_22:6__[M+H]__4.82** | | | 0.027 |
| Mean ± Standard deviation | 1,226,081.7 ± 661,465.6 | 1,736,502.7 ± 837,119.0 | |
| Median and [25%; 75%] | 1,183,102.0 [741,944.3; 1,511,735.4] | 1,813,868.5 [1,154,316.1; 2,235,182.0] | |
| **PC 38:1__[M+H]__6.84** | | | 0.003 |
| Mean ± Standard deviation | 570,389.2 ± 369,206.0 | 1,014,139.8 ± 700,518.4 | |
| Median and [25%; 75%] | 507,114.0 [386,641.6; 678,951.3] | 733,859.8 [595,696.0; 1,102,016.6] | |
| **PC 38:2 OA1__[M+H]__5.96** | | | 0.003 |
| Mean ± Standard deviation | 9,414,164.6 ± 4,718,697.7 | 14,511,014.3 ± 6,135,101.3 | |
| Median and [25%; 75%] | 8,876,921.0 [5,646,281.4; 11,411,705.9] | 12,762,794.8 [10,093,085.6; 17,331,065.9] | |
| **PC 38:2__[M+H]__6.31** | | | 0.001 |
| Mean ± Standard deviation | 9,344,557.0 ± 4,655,625.9 | 14,567,698.7 ± 6,023,157.7 | |
| Median and [25%; 75%] | 8,852,544.0 [5,657,980.6; 11,166,803.8] | 13,036,900.8 [10,134,836.4; 17,391,062.0] | |
| **PC 38:3 OA1__[M+H]__5.62** | | | <0.001 |
| Mean ± Standard deviation | 132,201,199.4 ± 52,650,699.2 | 204,825,541.8 ± 64,388,591.3 | |
| Median and [25%; 75%] | 126,086,756.0 [98,464,906.0; 163,302,7 | 194,634,992.0 [151,178,912.0; 251,247,322.0] | |
| **PC 38:3__[M+H]__6.19** | | | <0.001 |
| Mean ± Standard deviation | 128,777,964.8 ± 49,976,329.2 | 201,241,234.8 ± 61,922,461.5 | |
| Median and [25%; 75%] | 127,122,320.0 [96,399,764.0; 157,021,5 | 190,388,928.0 [153,923,376.0; 245,154,828.0] | |
| **PC 38:3\|PC 18:0_20:3__[M+H]__5.95** | | | <0.001 |

| | | | |
|---|---|---|---|
| Mean ± Standard deviation | 130,950,660.9 ± 51,144,125.5 | 207,586,227.5 ± 62,969,237.5 | |
| Median and [25%; 75%] | 131,022,884.0 [99,298,354.0; 156,908,1 | 199,399,946.0 [159,425,494.0; 250,064,162.0] | |
| **PC 38:4__[M+H]__5.36** | | | <0.001 |
| Mean ± Standard deviation | 30,581,383.0 ± 12,741,215.7 | 46,760,957.3 ± 18,204,219.0 | |
| Median and [25%; 75%] | 27,526,055.0 [19,197,921.5; 36,160,920 | 41,395,396.0 [35,053,029.0; 55,340,356.0] | |
| **PC 38:4\|PC 16:0_22:4__[M+H]__5.52** | | | <0.001 |
| Mean ± Standard deviation | 19,304,753.8 ± 15,774,180.2 | 39,451,377.2 ± 19,332,157.7 | |
| Median and [25%; 75%] | 17,974,938.5 [2,606,641.8; 30,673,889.0 | 33,768,166.8 [26,176,203.8; 50,478,402.0] | |
| **PC 38:4\|PC 18:0_20:4__[M+H]__5.76** | | | 0.248 |
| Mean ± Standard deviation | 288,475,405.3 ± 53,854,980.2 | 317,876,447.8 ± 93,042,940.9 | |
| Median and [25%; 75%] | 282,696,032.0 [255,534,958.0; 317,618, | 302,463,560.0 [248,493,929.0; 405,414,364.0] | |
| **PC 38:5\|PC 16:0_22:5__[M+H]__5.17** | | | 0.002 |
| Mean ± Standard deviation | 33,476,840.1 ± 12,555,590.4 | 48,045,358.5 ± 17,229,897.7 | |
| Median and [25%; 75%] | 31,935,471.0 [23,824,551.0; 39,999,146 | 41,367,990.0 [37,819,119.5; 54,448,078.0] | |
| **PC 38:5\|PC 18:1_20:4__[M+H]__5.34** | | | 0.005 |
| Mean ± Standard deviation | 75,847,845.6 ± 40,892,025.7 | 103,424,486.6 ± 36,134,205.4 | |
| Median and [25%; 75%] | 66,045,189.0 [51,613,341.5; 91,826,014 | 112,120,632.0 [70,255,925.5; 131,996,413.0] | |
| **PC 38:6\|PC 18:2_20:4__[M+H]__4.93** | | | 0.014 |
| Mean ± Standard deviation | 258,851,366.5 ± 54,946,396.8 | 312,986,325.8 ± 73,234,603.6 | |
| Median and [25%; 75%] | 250,315,720.0 [230,825,804.0; 301,524, | 301,147,042.0 [271,250,512.0; 358,289,532.0] | |
| **PC 39:4__[M+H]__6.06** | | | 0.013 |
| Mean ± Standard deviation | 489,090.2 ± 235,805.6 | 697,267.3 ± 326,512.4 | |
| Median and [25%; 75%] | 450,536.0 [354,933.0; 604,554.9] | 615,138.3 [511,901.3; 857,030.4] | |
| **PC 39:6__[M+H]__5.31** | | | 0.267 |
| Mean ± Standard deviation | 377,186.5 ± 370,122.9 | 472,176.6 ± 383,734.3 | |
| Median and [25%; 75%] | 256,251.5 [133,600.3; 489,559.9] | 366,450.5 [184,576.0; 744,123.7] | |
| **PC 40:4\|PC 18:0_22:4__[M+H]__6.13** | | | 0.002 |
| Mean ± Standard deviation | 8,419,047.2 ± 4,401,570.1 | 14,813,453.2 ± 8,107,489.1 | |
| Median and [25%; 75%] | 7,469,740.5 [4,943,366.6; 9,770,541.8] | 13,926,809.5 [8,287,211.5; 19,919,196.3] | |
| **PC 40:5__[M+H]__5.98** | | | 0.001 |
| Mean ± Standard deviation | 3,920,276.5 ± 3,053,772.9 | 8,717,713.4 ± 7,011,257.3 | |
| Median and [25%; 75%] | 3,449,844.0 [1,535,015.5; 4,931,707.5] | 6,249,138.0 [4,335,132.0; 9,305,417.7] | |
| **PC 40:5\|PC 18:0_22:5 OA1__[M+H]__5.98** | | | 0.001 |
| Mean ± Standard deviation | 3,995,835.3 ± 3,038,577.2 | 8,768,001.3 ± 7,001,574.4 | |
| Median and [25%; 75%] | 3,449,844.0 [1,586,874.9; 5,157,097.0] | 6,249,138.0 [4,335,132.0; 9,305,417.7] | |
| **PC 40:5\|PC 18:0_22:5__[M+H]__5.71** | | | 0.007 |

| | | | |
|---|---|---|---|
| Mean ± Standard deviation | 5,599,962.6 ± 2,644,547.8 | 8,132,631.2 ± 3,850,239.0 | |
| Median and [25%; 75%] | 5,202,176.5 [3,904,794.3; 6,475,210.6] | 6,889,683.5 [5,955,693.9; 9,457,590.9] | |
| **PC 40:6__[M+H]__5.35** | | | 0.016 |
| Mean ± Standard deviation | 120,026,639.9 ± 37,414,394.0 | 150,736,241.8 ± 45,815,772.2 | |
| Median and [25%; 75%] | 110,671,988.0 [90,134,746.0; 142,094,7[illegible] | 153,669,376.0 [137,594,307.0; 166,808,593.0] | |
| **PC 40:7\|PC 18:1_22:6__[M+H]__5.15** | | | 0.078 |
| Mean ± Standard deviation | 7,819,612.0 ± 3,366,645.0 | 10,308,011.6 ± 5,552,092.4 | |
| Median and [25%; 75%] | 7,142,071.5 [5,327,694.8; 9,822,894.4] | 9,689,348.3 [7,317,289.6; 11,734,062.9] | |
| **PC 40:8__[M+H]__4.81** | | | 0.001 |
| Mean ± Standard deviation | 1,327,850.9 ± 741,825.9 | 2,143,160.9 ± 990,322.4 | |
| Median and [25%; 75%] | 1,118,006.0 [893,739.5; 1,800,099.0] | 1,838,797.3 [1,419,057.1; 2,677,445.4] | |
| **PC 40:8\|PC 20:4_20:4__[M+H]__4.86** | | | <0.001 |
| Mean ± Standard deviation | 1,237,909.7 ± 572,084.5 | 2,168,218.6 ± 989,679.0 | |
| Median and [25%; 75%] | 1,154,851.5 [813,358.1; 1,531,277.0] | 1,988,822.3 [1,400,874.8; 2,705,398.5] | |
| **PC 43:5__[M+H]__7.51** | | | 0.146 |
| Mean ± Standard deviation | 621,337.9 ± 279,145.9 | 869,333.5 ± 517,286.9 | |
| Median and [25%; 75%] | 533,029.5 [428,442.5; 868,277.5] | 726,996.9 [570,443.5; 973,861.5] | |
| **PC O-31:0__[M+H]__5.08** | | | 0.009 |
| Mean ± Standard deviation | 678,572.3 ± 341,707.0 | 1,282,282.5 ± 829,640.6 | |
| Median and [25%; 75%] | 627,603.5 [431,584.5; 874,915.5] | 1,209,394.8 [615,653.4; 1,601,182.4] | |
| **PC O-32:0\|PC O-16:0_16:0__[M+H]__5.93** | | | 0.037 |
| Mean ± Standard deviation | 3,460,020.3 ± 1,975,331.0 | 4,429,735.8 ± 1,817,941.9 | |
| Median and [25%; 75%] | 3,117,513.5 [2,193,301.4; 4,519,050.0] | 4,137,403.8 [3,570,519.3; 5,561,001.8] | |
| **PC O-32:1__[M+H]__5.89** | | | 0.011 |
| Mean ± Standard deviation | 2,313,975.2 ± 1,229,029.0 | 3,230,681.0 ± 1,234,978.2 | |
| Median and [25%; 75%] | 2,129,465.5 [1,410,576.3; 2,736,948.3] | 2,920,611.1 [2,073,290.9; 4,157,257.4] | |
| **PC O-32:1\|PC O-16:1_16:0__[M+H]__5.91** | | | 0.011 |
| Mean ± Standard deviation | 2,302,325.0 ± 1,238,449.8 | 3,230,719.5 ± 1,234,974.7 | |
| Median and [25%; 75%] | 2,129,465.5 [1,362,591.8; 2,736,948.3] | 2,920,919.1 [2,073,290.9; 4,157,257.4] | |
| **PC O-33:1__[M+H]__5.61** | | | 0.161 |
| Mean ± Standard deviation | 5,917,070.3 ± 2,023,859.7 | 7,041,481.6 ± 2,563,852.8 | |
| Median and [25%; 75%] | 5,389,843.5 [4,403,451.0; 7,173,576.5] | 6,617,247.0 [5,156,622.9; 9,152,650.8] | |
| **PC O-33:2__[M+H]__4.79** | | | <0.001 |
| Mean ± Standard deviation | 1,914,084.2 ± 1,993,333.0 | 4,908,627.7 ± 3,043,643.9 | |
| Median and [25%; 75%] | 1,232,601.3 [785,458.9; 1,896,157.3] | 4,003,082.6 [2,991,094.2; 5,714,821.1] | |
| **PC O-34:2\|PC O-16:0_18:2__[M+H]__5.0** | | | 0.011 |

| | | | |
|---|---|---|---|
| Mean ± Standard deviation | 1,145,092.8 ± 678,361.1 | 1,921,058.1 ± 1,167,463.0 | |
| Median and [25%; 75%] | 985,902.5 [678,553.5; 1,556,431.8] | 1,759,600.1 [1,052,798.0; 2,772,459.5] | |
| **PC O-34:2\|PC O-16:1_18:1__[M+H]__6.03** | | | 0.006 |
| Mean ± Standard deviation | 5,659,561.8 ± 2,454,512.1 | 9,413,917.9 ± 5,281,627.7 | |
| Median and [25%; 75%] | 4,855,674.3 [4,098,794.0; 6,561,596.0] | 8,596,877.0 [4,745,787.8; 11,287,265.0] | |
| **PC O-34:3\|PC O-16:1_18:2__[M+H]__5.55** | | | <0.001 |
| Mean ± Standard deviation | 12,273,881.8 ± 5,780,534.7 | 24,926,270.3 ± 19,114,610.3 | |
| Median and [25%; 75%] | 10,872,357.0 [8,142,126.8; 14,638,976.[illegible] | 15,804,905.4 [13,542,661.1; 31,042,744.5] | |
| **PC O-35:0__[M+H]__5.67** | | | 0.001 |
| Mean ± Standard deviation | 3,742,179.1 ± 2,542,167.0 | 5,168,853.8 ± 2,243,739.6 | |
| Median and [25%; 75%] | 3,191,427.0 [2,215,281.3; 4,098,943.3] | 4,797,468.8 [3,607,627.0; 5,682,585.8] | |
| **PC O-35:0\|PC O-17:0_18:0__[M+H]__6.12** | | | <0.001 |
| Mean ± Standard deviation | 1,833,761.3 ± 935,212.9 | 3,138,921.1 ± 1,173,979.1 | |
| Median and [25%; 75%] | 1,443,756.0 [1,233,669.3; 2,307,449.8] | 3,031,582.3 [2,216,726.3; 3,856,602.6] | |
| **PC O-35:0\|PC O-19:0_16:0__[M+H]__6.12** | | | <0.001 |
| Mean ± Standard deviation | 1,833,761.3 ± 935,212.9 | 3,138,921.1 ± 1,173,979.1 | |
| Median and [25%; 75%] | 1,443,756.0 [1,233,669.3; 2,307,449.8] | 3,031,582.3 [2,216,726.3; 3,856,602.6] | |
| **PC O-35:1__[M+H]__5.67** | | | <0.001 |
| Mean ± Standard deviation | 398,114,155.4 ± 100,400,563.2 | 537,432,887.5 ± 176,578,399.4 | |
| Median and [25%; 75%] | 397,847,792.0 [326,342,602.0; 439,680,[illegible] | 491,725,928.0 [435,508,096.0; 588,123,544.0] | |
| **PC O-35:2__[M+H]__5.74** | | | 0.002 |
| Mean ± Standard deviation | 595,740,089.1 ± 133,704,423.3 | 767,507,147.5 ± 171,925,539.4 | |
| Median and [25%; 75%] | 611,402,912.0 [516,783,856.0; 679,934,[illegible] | 794,001,712.0 [593,857,368.0; 891,947,720.0] | |
| **PC O-35:4\|PC O-15:0_20:4__[M+H]__4.76** | | | <0.001 |
| Mean ± Standard deviation | 431,583.2 ± 983,726.1 | 641,216.4 ± 333,784.7 | |
| Median and [25%; 75%] | 152,149.5 [70,428.8; 262,496.3] | 501,346.3 [432,866.6; 836,325.0] | |
| **PC O-36:1 OA1__[M+H]__5.92** | | | <0.001 |
| Mean ± Standard deviation | 848,131.1 ± 479,016.7 | 1,616,985.0 ± 941,820.3 | |
| Median and [25%; 75%] | 757,673.5 [539,757.0; 1,054,300.3] | 1,498,328.0 [1,119,661.4; 1,723,549.4] | |
| **PC O-36:1__[M+H]__6.69** | | | <0.001 |
| Mean ± Standard deviation | 3,786,106.4 ± 1,647,606.9 | 6,605,863.6 ± 2,950,352.5 | |
| Median and [25%; 75%] | 3,485,434.0 [2,521,574.3; 4,834,408.5] | 5,797,526.3 [4,594,981.1; 8,487,831.0] | |
| **PC O-36:1\|PC O-18:0_18:1__[M+H]__5.93** | | | <0.001 |
| Mean ± Standard deviation | 848,955.9 ± 478,559.6 | 1,617,327.7 ± 941,701.9 | |
| Median and [25%; 75%] | 757,673.5 [541,626.1; 1,055,598.3] | 1,498,328.0 [1,119,661.4; 1,723,549.4] | |
| **PC O-36:2__[M+H]__5.5** | | | <0.001 |

| | | | |
|---|---|---|---|
| Mean ± Standard deviation | 5,117,535.0 ± 2,542,358.2 | 11,126,268.8 ± 6,343,137.0 | |
| Median and [25%; 75%] | 4,732,760.5 [3,504,939.6; 5,844,434.0] | 9,367,344.5 [6,070,747.6; 14,903,774.4] | |
| **PC O-36:2\|PC O-18:1_18:1__[M+H]__6.65** | | | 0.052 |
| Mean ± Standard deviation | 306,422.5 ± 164,222.7 | 560,918.1 ± 429,826.4 | |
| Median and [25%; 75%] | 280,635.5 [193,098.1; 373,634.8] | 403,088.6 [249,883.6; 757,196.6] | |
| **PC O-36:3\|PC O-18:1_18:2__[M+H]__5.7** | | | 0.306 |
| Mean ± Standard deviation | 813,279.8 ± 1,023,652.3 | 1,302,760.1 ± 1,332,774.8 | |
| Median and [25%; 75%] | 493,064.0 [273,543.9; 927,460.0] | 783,364.3 [282,448.1; 2,029,892.9] | |
| **PC O-36:4\|PC O-16:1_20:3__[M+H]__5.7** | | | 0.033 |
| Mean ± Standard deviation | 35,520,010.5 ± 15,899,209.3 | 43,611,872.6 ± 15,268,233.2 | |
| Median and [25%; 75%] | 32,126,746.0 [25,833,157.5; 39,871,449 | 41,528,005.0 [33,835,698.3; 53,872,419.5] | |
| **PC O-36:5__[M+H]__5.4** | | | <0.001 |
| Mean ± Standard deviation | 32,396,147.5 ± 10,073,466.7 | 46,994,633.1 ± 15,006,004.9 | |
| Median and [25%; 75%] | 29,982,879.0 [25,797,302.3; 37,713,654 | 45,216,760.0 [40,055,929.3; 56,642,019.5] | |
| **PC O-36:5\|PC O-16:0_20:5__[M+H]__5.16** | | | 0.080 |
| Mean ± Standard deviation | 205,726.5 ± 523,202.9 | 419,252.1 ± 680,407.7 | |
| Median and [25%; 75%] | 97,497.5 [44,820.8; 190,052.9] | 150,134.1 [107,465.6; 331,600.9] | |
| **PC O-36:5\|PC O-16:1_20:4__[M+H]__5.5** | | | <0.001 |
| Mean ± Standard deviation | 32,423,393.5 ± 10,097,908.1 | 47,033,299.8 ± 15,023,469.8 | |
| Median and [25%; 75%] | 29,957,984.0 [25,798,597.8; 37,718,497 | 45,254,052.0 [40,058,986.3; 56,640,551.5] | |
| **PC O-36:7 OA1__[M+H]__6.13** | | | 0.027 |
| Mean ± Standard deviation | 622,433.9 ± 346,677.6 | 861,810.8 ± 350,467.7 | |
| Median and [25%; 75%] | 547,885.8 [372,263.0; 877,617.8] | 735,819.9 [643,153.1; 999,955.1] | |
| **PC O-36:7__[M+H]__5.67** | | | 0.031 |
| Mean ± Standard deviation | 630,074.5 ± 342,904.8 | 862,027.0 ± 350,472.1 | |
| Median and [25%; 75%] | 547,885.8 [388,757.9; 877,638.8] | 735,819.9 [643,153.1; 1,000,148.4] | |
| **PC O-37:0__[M+H]__6.26** | | | <0.001 |
| Mean ± Standard deviation | 432,940.4 ± 372,263.8 | 1,192,205.4 ± 1,563,996.1 | |
| Median and [25%; 75%] | 364,847.0 [237,067.8; 472,005.1] | 831,277.0 [387,443.6; 1,237,594.8] | |
| **PC O-37:1 OA1__[M+H]__6.96** | | | <0.001 |
| Mean ± Standard deviation | 16,111,333.0 ± 5,816,974.3 | 25,387,641.3 ± 8,318,291.9 | |
| Median and [25%; 75%] | 15,469,463.5 [12,129,478.6; 18,582,029 | 22,688,954.0 [20,477,848.0; 31,568,561.1] | |
| **PC O-37:1 OA2__[M+H]__6.27** | | | <0.001 |
| Mean ± Standard deviation | 54,783,037.6 ± 28,387,562.2 | 127,684,269.3 ± 127,967,711.7 | |
| Median and [25%; 75%] | 50,210,815.0 [38,173,788.5; 60,917,530 | 101,766,002.0 [64,393,505.0; 138,414,268.0] | |
| **PC O-37:1__[M+H]__5.81** | | | <0.001 |

| | | | |
|---|---|---|---|
| Mean ± Standard deviation | 4,014,426.4 ± 1,945,606.5 | 6,313,166.5 ± 2,461,119.1 | |
| Median and [25%; 75%] | 3,487,752.5 [2,785,270.5; 4,749,538.8] | 5,910,743.8 [4,871,722.6; 7,352,212.3] | |
| **PC O-37:2 OA1__[M+H]__6.41** | | | <0.001 |
| Mean ± Standard deviation | 326,708,967.8 ± 168,891,320.8 | 585,899,843.9 ± 187,317,087.3 | |
| Median and [25%; 75%] | 360,870,416.0 [246,640,304.0; 443,517, | 568,372,192.0 [400,149,268.0; 693,196,856.0] | |
| **PC O-37:2__[M+H]__5.38** | | | <0.001 |
| Mean ± Standard deviation | 2,081,963.3 ± 1,665,459.7 | 3,316,875.4 ± 1,268,843.7 | |
| Median and [25%; 75%] | 1,610,832.0 [1,256,044.0; 2,184,277.5] | 3,189,905.0 [2,053,880.0; 4,009,051.8] | |
| **PC O-37:3__[M+H]__5.38** | | | <0.001 |
| Mean ± Standard deviation | 221,125,231.6 ± 93,001,259.6 | 348,191,377.5 ± 83,424,245.3 | |
| Median and [25%; 75%] | 213,307,196.0 [153,837,977.0; 262,149, | 355,240,528.0 [282,287,386.0; 404,754,424.0] | |
| **PC O-37:3\|PC O-17:0_20:3__[M+H]__5.38** | | | <0.001 |
| Mean ± Standard deviation | 220,896,139.8 ± 92,930,536.7 | 347,837,630.3 ± 83,676,002.7 | |
| Median and [25%; 75%] | 212,651,488.0 [153,632,362.0; 262,327, | 354,454,816.0 [281,129,812.0; 404,779,276.0] | |
| **PC O-37:8 OA1__[M+H]__5.94** | | | 0.093 |
| Mean ± Standard deviation | 198,336.0 ± 130,196.4 | 229,514.1 ± 90,913.0 | |
| Median and [25%; 75%] | 157,320.0 [114,135.6; 253,789.8] | 236,537.0 [182,574.7; 279,916.6] | |
| **PC O-37:8\|PC O-18:4_19:4__[M+H]__4.75** | | | 0.036 |
| Mean ± Standard deviation | 136,826.2 ± 100,942.2 | 208,806.9 ± 121,665.1 | |
| Median and [25%; 75%] | 127,265.0 [59,445.5; 178,123.3] | 158,432.3 [126,251.9; 252,059.4] | |
| **PC O-38:1__[M+H]__7.34** | | | <0.001 |
| Mean ± Standard deviation | 7,528,308.3 ± 2,816,461.3 | 12,343,155.9 ± 4,673,580.0 | |
| Median and [25%; 75%] | 7,619,393.0 [5,347,834.5; 9,000,176.4] | 10,429,422.8 [9,101,962.5; 15,478,609.9] | |
| **PC O-38:2 OA1__[M+H]__6.62** | | | 0.104 |
| Mean ± Standard deviation | 3,089,607.6 ± 1,657,965.1 | 3,688,412.7 ± 1,403,436.3 | |
| Median and [25%; 75%] | 2,923,690.5 [2,008,082.8; 3,750,036.5] | 3,224,359.5 [2,831,842.3; 4,145,465.8] | |
| **PC O-38:2__[M+H]__6.76** | | | <0.001 |
| Mean ± Standard deviation | 4,240,982.1 ± 1,601,855.3 | 7,172,746.2 ± 2,943,162.2 | |
| Median and [25%; 75%] | 4,414,305.0 [3,212,538.3; 5,056,062.8] | 6,295,250.8 [5,467,354.3; 7,642,679.6] | |
| **PC O-38:2\|PC O-20:0_18:2__[M+H]__6.09** | | | <0.001 |
| Mean ± Standard deviation | 237,920.7 ± 127,130.5 | 707,430.4 ± 525,986.4 | |
| Median and [25%; 75%] | 220,569.0 [155,050.6; 296,722.5] | 588,298.5 [287,910.3; 884,480.4] | |
| **PC O-38:3__[M+H]__6.16** | | | 0.012 |
| Mean ± Standard deviation | 1,863,478.2 ± 597,521.2 | 2,520,404.7 ± 874,302.6 | |
| Median and [25%; 75%] | 1,764,185.3 [1,438,671.5; 2,276,344.4] | 2,494,334.0 [1,962,669.1; 3,039,843.1] | |
| **PC O-38:3\|PC O-18:0_20:3 OA1__[M+H]__5.64** | | | 0.003 |

| | | | |
|---|---|---|---|
| Mean ± Standard deviation | 56,629.3 ± 45,436.2 | 86,555.6 ± 47,622.2 | |
| Median and [25%; 75%] | 40,530.0 [27,853.0; 73,050.3] | 78,789.8 [61,865.5; 98,615.2] | |
| **PC O-38:3\|PC O-18:0_20:3__[M+H]__6.35** | | | 0.132 |
| Mean ± Standard deviation | 207,702.6 ± 155,310.1 | 329,763.1 ± 314,005.3 | |
| Median and [25%; 75%] | 156,308.8 [112,090.3; 283,615.8] | 233,184.5 [152,369.6; 379,850.1] | |
| **PC O-38:4__[M+H]__5.48** | | | 0.807 |
| Mean ± Standard deviation | 3,201,997.6 ± 2,845,913.6 | 3,001,145.7 ± 1,753,821.5 | |
| Median and [25%; 75%] | 2,244,997.0 [1,532,783.3; 3,798,516.5] | 2,894,058.9 [1,466,026.4; 4,610,678.9] | |
| **PC O-38:4\|PC O-16:0_22:4__[M+H]__5.91** | | | 0.899 |
| Mean ± Standard deviation | 2,297,681.9 ± 1,235,135.4 | 2,223,870.8 ± 1,015,578.7 | |
| Median and [25%; 75%] | 2,000,808.0 [1,300,783.5; 3,218,246.3] | 2,308,522.5 [1,384,719.7; 2,981,740.5] | |
| **PC O-38:5 OA1__[M+H]__6.09** | | | <0.001 |
| Mean ± Standard deviation | 8,121,001.7 ± 2,940,433.9 | 13,205,359.7 ± 5,233,232.8 | |
| Median and [25%; 75%] | 7,045,902.8 [6,379,813.9; 9,091,485.0] | 12,976,402.4 [9,458,920.3; 17,202,732.9] | |
| **PC O-38:5__[M+H]__5.62** | | | 0.755 |
| Mean ± Standard deviation | 18,366,481.7 ± 9,890,684.4 | 17,749,514.1 ± 7,138,335.6 | |
| Median and [25%; 75%] | 15,830,477.5 [12,586,949.3; 20,848,235 | 17,907,232.0 [13,636,791.4; 21,118,025.3] | |
| **PC O-38:5\|PC O-16:1_22:4__[M+H]__5.9** | | | 0.679 |
| Mean ± Standard deviation | 18,373,715.4 ± 9,825,544.5 | 17,877,816.4 ± 7,096,277.7 | |
| Median and [25%; 75%] | 15,597,263.5 [12,547,506.0; 20,922,416 | 17,940,038.8 [13,747,836.5; 21,261,931.9] | |
| **PC O-38:5\|PC O-18:4_20:1__[M+H]__5.51** | | | 0.655 |
| Mean ± Standard deviation | 18,986,689.7 ± 9,843,875.2 | 18,728,240.1 ± 7,143,731.9 | |
| Median and [25%; 75%] | 16,545,390.5 [13,033,465.8; 21,538,000 | 18,226,667.3 [14,588,576.6; 21,940,853.9] | |
| **PC O-38:6\|PC O-16:0_22:6 OA1__[M+H]__4.82** | | | 0.306 |
| Mean ± Standard deviation | 212,974.0 ± 171,849.6 | 260,728.7 ± 188,029.4 | |
| Median and [25%; 75%] | 167,179.5 [71,802.3; 300,384.5] | 237,929.5 [118,087.5; 358,181.3] | |
| **PC O-38:6\|PC O-16:0_22:6 OA2__[M+H]__5.36** | | | 0.704 |
| Mean ± Standard deviation | 2,045,774.8 ± 1,241,345.7 | 2,383,199.3 ± 1,897,455.0 | |
| Median and [25%; 75%] | 1,599,872.8 [1,226,076.8; 2,803,147.3] | 2,005,523.3 [1,284,480.9; 2,424,279.3] | |
| **PC O-38:6\|PC O-16:0_22:6__[M+H]__5.35** | | | 0.692 |
| Mean ± Standard deviation | 2,031,361.2 ± 1,231,398.6 | 2,381,717.2 ± 1,897,901.0 | |
| Median and [25%; 75%] | 1,599,872.8 [1,218,381.5; 2,790,540.0] | 1,993,666.6 [1,284,480.9; 2,424,279.3] | |
| **PC O-38:7__[M+H]__6.26** | | | <0.001 |
| Mean ± Standard deviation | 108,773.1 ± 59,635.5 | 255,261.0 ± 228,844.9 | |
| Median and [25%; 75%] | 88,242.5 [64,190.5; 146,554.0] | 186,252.5 [115,327.6; 281,734.8] | |
| **PC O-38:7\|PC O-16:1_22:6__[M+H]__5.32** | | | 0.322 |

| | | | |
|---|---|---|---|
| Mean ± Standard deviation | 793,305.4 ± 582,825.3 | 1,048,183.7 ± 747,453.6 | |
| Median and [25%; 75%] | 712,973.5 [320,859.8; 1,081,337.3] | 710,856.6 [562,448.3; 1,455,974.4] | |
| **PC O-38:9__[M+H]__5.39** | | | <0.001 |
| Mean ± Standard deviation | 355,524.9 ± 218,403.2 | 628,675.3 ± 302,799.8 | |
| Median and [25%; 75%] | 333,074.5 [200,958.4; 486,441.5] | 530,724.5 [448,685.6; 734,610.5] | |
| **PC O-39:1__[M+H]__7.73** | | | <0.001 |
| Mean ± Standard deviation | 13,320,198.5 ± 5,269,707.2 | 20,204,882.3 ± 6,943,488.0 | |
| Median and [25%; 75%] | 13,149,171.0 [10,079,471.9; 16,819,077 | 19,645,099.5 [14,858,568.0; 22,700,099.8] | |
| **PC O-39:2__[M+H]__7.14** | | | <0.001 |
| Mean ± Standard deviation | 8,529,005.3 ± 3,649,036.7 | 12,193,227.9 ± 3,043,989.6 | |
| Median and [25%; 75%] | 8,829,649.0 [5,456,278.4; 10,931,365.5] | 12,353,113.6 [9,655,628.4; 12,860,552.8] | |
| **PC O-39:2\|PC O-19:0_20:2__[M+H]__6.31** | | | 0.001 |
| Mean ± Standard deviation | 1,299,329.2 ± 715,899.2 | 2,188,755.1 ± 862,518.7 | |
| Median and [25%; 75%] | 1,297,627.0 [871,656.0; 1,673,592.4] | 1,850,226.3 [1,584,214.5; 2,781,903.0] | |
| **PC O-39:3__[M+H]__6.37** | | | 0.011 |
| Mean ± Standard deviation | 23,028,165.4 ± 7,783,608.6 | 28,866,846.0 ± 7,187,358.8 | |
| Median and [25%; 75%] | 21,646,140.0 [17,640,031.8; 28,241,160 | 26,668,819.0 [24,195,987.3; 33,949,745.8] | |
| **PC O-39:4 OA1__[M+H]__5.51** | | | 0.794 |
| Mean ± Standard deviation | 234,788,294.1 ± 133,021,414.4 | 215,920,424.4 ± 113,474,093.0 | |
| Median and [25%; 75%] | 199,665,532.0 [133,874,351.0; 270,535, | 209,212,382.0 [135,827,874.0; 326,355,288.0] | |
| **PC O-39:4__[M+H]__5.74** | | | 0.768 |
| Mean ± Standard deviation | 237,270,543.7 ± 133,179,089.3 | 216,056,180.9 ± 113,427,290.2 | |
| Median and [25%; 75%] | 201,074,420.0 [134,205,567.0; 270,482, | 209,001,658.0 [138,771,160.0; 326,860,036.0] | |
| **PC O-39:5 OA1__[M+H]__5.34** | | | 0.005 |
| Mean ± Standard deviation | 8,678,977.6 ± 4,513,012.6 | 12,587,988.2 ± 4,865,265.5 | |
| Median and [25%; 75%] | 7,295,068.8 [5,807,144.8; 11,019,827.5] | 11,609,148.3 [10,306,345.6; 14,923,030.1] | |
| **PC O-39:5 OA2__[M+H]__5.18** | | | 0.072 |
| Mean ± Standard deviation | 23,570,434.1 ± 22,266,051.0 | 26,353,579.4 ± 13,093,260.7 | |
| Median and [25%; 75%] | 14,105,400.0 [9,560,230.4; 25,201,123.0 | 23,880,879.3 [14,808,519.0; 36,452,431.5] | |
| **PC O-39:5__[M+H]__5.05** | | | 0.374 |
| Mean ± Standard deviation | 3,796,621.6 ± 2,349,964.6 | 4,178,263.5 ± 1,807,192.9 | |
| Median and [25%; 75%] | 3,146,945.0 [2,066,697.4; 4,626,222.8] | 4,353,074.8 [2,733,529.8; 5,539,495.8] | |
| **PC O-39:6__[M+H]__4.93** | | | 0.228 |
| Mean ± Standard deviation | 244,373,673.6 ± 90,001,280.7 | 287,246,897.0 ± 126,573,785.5 | |
| Median and [25%; 75%] | 232,479,724.0 [172,626,884.0; 316,531, | 305,544,740.0 [200,781,136.0; 349,845,942.0] | |
| **PC O-40:10 OA1__[M+H]__5.75** | | | 0.322 |

| | | | |
|---|---|---|---|
| Mean ± Standard deviation | 385,605.4 ± 214,069.6 | 467,643.4 ± 270,470.0 | |
| Median and [25%; 75%] | 371,645.0 [236,737.3; 511,784.8] | 421,534.0 [270,144.1; 600,017.0] | |
| **PC O-40:2__[M+H]__7.21** | | | 0.030 |
| Mean ± Standard deviation | 728,968.0 ± 449,716.5 | 990,711.6 ± 452,237.1 | |
| Median and [25%; 75%] | 606,551.5 [391,604.0; 978,065.5] | 902,845.0 [729,735.1; 1,272,952.9] | |
| **PC O-40:4\|PC O-18:0_22:4__[M+H]__6.55** | | | 0.013 |
| Mean ± Standard deviation | 599,964.2 ± 266,914.0 | 932,419.9 ± 602,498.0 | |
| Median and [25%; 75%] | 563,923.5 [418,192.9; 667,251.0] | 773,828.0 [638,031.8; 1,227,134.6] | |
| **PC O-40:4\|PC O-20:0_20:4__[M+H]__6.83** | | | <0.001 |
| Mean ± Standard deviation | 1,807,013.9 ± 677,210.0 | 2,963,139.2 ± 1,389,175.8 | |
| Median and [25%; 75%] | 1,836,995.5 [1,407,346.3; 2,251,911.0] | 3,136,193.3 [2,412,772.5; 3,398,248.3] | |
| **PC O-40:6\|PC O-18:0_22:6 OA1__[M+H]__5.91** | | | 0.571 |
| Mean ± Standard deviation | 1,378,211.2 ± 782,532.0 | 1,612,227.5 ± 974,942.7 | |
| Median and [25%; 75%] | 1,250,906.0 [778,888.5; 1,701,349.3] | 1,239,529.3 [871,329.5; 2,361,952.9] | |
| **PC O-40:6\|PC O-18:0_22:6__[M+H]__5.31** | | | 0.537 |
| Mean ± Standard deviation | 47,133.5 ± 35,756.5 | 51,379.9 ± 33,177.1 | |
| Median and [25%; 75%] | 37,218.5 [23,805.8; 63,143.3] | 46,988.0 [28,760.9; 67,146.6] | |
| **PC O-40:7\|PC O-18:1_22:6__[M+H]__5.45** | | | 0.560 |
| Mean ± Standard deviation | 1,663,112.6 ± 1,049,135.7 | 1,498,422.3 ± 1,092,728.2 | |
| Median and [25%; 75%] | 1,473,208.0 [744,120.1; 2,458,410.9] | 1,140,758.5 [944,359.1; 1,639,721.4] | |
| **PC O-41:5__[M+H]__5.57** | | | 0.260 |
| Mean ± Standard deviation | 1,015,672.6 ± 808,737.1 | 1,311,889.3 ± 1,012,741.4 | |
| Median and [25%; 75%] | 748,769.5 [388,772.3; 1,270,767.6] | 1,016,579.3 [606,145.3; 1,690,205.5] | |
| **PC O-41:5\|PC O-19:0_22:5__[M+H]__5.99** | | | 0.022 |
| Mean ± Standard deviation | 829,804.8 ± 1,045,979.3 | 1,723,787.9 ± 2,148,773.1 | |
| Median and [25%; 75%] | 525,358.0 [202,647.6; 825,143.5] | 896,672.1 [458,140.6; 1,531,324.9] | |
| **PC O-41:6__[M+H]__5.57** | | | 0.253 |
| Mean ± Standard deviation | 59,575,103.9 ± 35,421,838.4 | 77,645,414.7 ± 49,704,010.7 | |
| Median and [25%; 75%] | 51,150,176.0 [30,712,044.5; 79,108,151 | 72,342,559.0 [45,690,011.0; 100,595,921.0] | |
| **PC O-41:6\|PC O-17:0_24:6__[M+H]__5.35** | | | 0.253 |
| Mean ± Standard deviation | 59,645,439.1 ± 35,483,005.0 | 77,688,324.1 ± 49,731,179.2 | |
| Median and [25%; 75%] | 51,183,486.0 [30,720,498.0; 79,170,965 | 72,383,259.5 [45,704,589.0; 100,627,716.0] | |
| **PC O-41:7__[M+H]__5.15** | | | 0.137 |
| Mean ± Standard deviation | 1,462,841.6 ± 1,442,744.6 | 1,857,625.7 ± 1,423,605.4 | |
| Median and [25%; 75%] | 984,762.5 [561,298.5; 1,709,766.0] | 1,690,537.3 [941,674.4; 2,068,279.3] | |
| **PC O-41:8\|PC O-19:2_22:6__[M+H]__4.78** | | | 0.036 |

| | | | |
|---|---|---|---|
| Mean ± Standard deviation | 271,713.9 ± 236,948.3 | 399,019.4 ± 259,074.1 | |
| Median and [25%; 75%] | 217,319.0 [106,024.8; 323,532.4] | 304,721.0 [183,939.9; 551,131.3] | |
| **PC O-42:4\|PC O-22:0_20:4__[M+H]__7.57** | | | <0.001 |
| Mean ± Standard deviation | 1,302,159.0 ± 426,634.4 | 2,054,957.5 ± 871,335.3 | |
| Median and [25%; 75%] | 1,312,822.5 [1,098,697.9; 1,508,629.5] | 2,086,828.8 [1,694,159.5; 2,448,877.5] | |
| **PC O-42:5\|PC O-22:1_20:4__[M+H]__6.81** | | | 0.018 |
| Mean ± Standard deviation | 2,828,463.3 ± 1,151,040.4 | 4,087,115.8 ± 2,237,961.1 | |
| Median and [25%; 75%] | 2,657,894.3 [2,125,169.1; 3,430,664.3] | 3,968,943.8 [2,789,710.0; 4,867,102.3] | |
| **PC O-44:6__[M+H]__6.89** | | | 0.583 |
| Mean ± Standard deviation | 1,507,140.0 ± 1,213,179.8 | 1,869,200.7 ± 1,875,413.1 | |
| Median and [25%; 75%] | 1,053,051.0 [676,947.3; 1,935,018.8] | 1,238,216.0 [900,001.1; 1,948,146.8] | |
| **PC O-44:6\|PC O-24:2_20:4__[M+H]__6.87** | | | 0.980 |
| Mean ± Standard deviation | 1,667,222.4 ± 1,181,705.7 | 1,871,006.5 ± 1,875,808.1 | |
| Median and [25%; 75%] | 1,268,671.5 [782,171.5; 2,438,630.5] | 1,166,371.0 [921,142.6; 1,962,220.6] | |
| **PC O-44:7\|PC O-22:1_22:6__[M+H]__6.55** | | | 0.156 |
| Mean ± Standard deviation | 289,139.4 ± 150,266.8 | 415,726.3 ± 315,643.6 | |
| Median and [25%; 75%] | 300,407.0 [167,489.5; 363,459.3] | 335,215.8 [232,040.3; 482,422.0] | |
| **PC O-46:7\|PC O-24:1_22:6__[M+H]__7.24** | | | 0.084 |
| Mean ± Standard deviation | 359,526.3 ± 231,401.3 | 559,715.4 ± 444,188.7 | |
| Median and [25%; 75%] | 303,145.5 [185,694.0; 455,675.5] | 413,444.3 [290,681.0; 560,556.1] | |
| **PE 34:1\|PE 16:0_18:1__[M+H]__5.83** | | | 0.057 |
| Mean ± Standard deviation | 142,931.6 ± 89,758.9 | 330,242.7 ± 576,733.2 | |
| Median and [25%; 75%] | 139,977.5 [70,458.8; 185,597.1] | 160,549.0 [110,507.1; 285,733.6] | |
| **PE 34:2\|PE 16:0_18:2__[M+H]__5.37** | | | 0.026 |
| Mean ± Standard deviation | 91,840.6 ± 100,807.5 | 476,222.1 ± 1,130,090.7 | |
| Median and [25%; 75%] | 61,254.0 [29,188.3; 116,142.8] | 123,141.0 [58,976.9; 304,820.8] | |
| **PE 36:1__[M+H]__6.44** | | | 0.026 |
| Mean ± Standard deviation | 904,733.2 ± 497,305.9 | 1,279,443.1 ± 597,610.9 | |
| Median and [25%; 75%] | 809,743.0 [531,861.3; 1,227,278.4] | 1,194,873.3 [826,726.9; 1,627,459.4] | |
| **PE 36:2__[M+H]__5.92** | | | <0.001 |
| Mean ± Standard deviation | 1,336,827.4 ± 913,710.5 | 4,838,632.9 ± 4,316,359.9 | |
| Median and [25%; 75%] | 1,136,781.5 [676,494.3; 1,849,399.3] | 3,145,602.5 [1,707,046.8; 6,750,458.2] | |
| **PE 36:2\|PE 18:1_18:1__[M+H]__5.93** | | | 0.043 |
| Mean ± Standard deviation | 438,476.0 ± 539,206.9 | 850,418.9 ± 870,985.5 | |
| Median and [25%; 75%] | 310,596.5 [143,897.1; 537,207.3] | 509,636.5 [220,557.8; 1,108,641.6] | |
| **PE 36:4__[M+H]__5.34** | | | 0.452 |

| | | | |
|---|---|---|---|
| Mean ± Standard deviation | 220,694.8 ± 239,651.6 | 354,254.5 ± 478,125.3 | |
| Median and [25%; 75%] | 145,059.5 [61,175.0; 257,450.1] | 190,248.0 [73,913.1; 305,980.3] | |
| **PE 38:4__[M+H]__5.9** | | | 0.274 |
| Mean ± Standard deviation | 3,903,178.0 ± 2,348,135.9 | 6,374,614.5 ± 7,053,536.1 | |
| Median and [25%; 75%] | 3,713,450.5 [1,907,073.8; 5,174,304.6] | 4,111,465.8 [2,390,956.7; 8,039,766.3] | |
| **PE 38:6\|PE 16:0_22:6__[M+H]__5.16** | | | 0.442 |
| Mean ± Standard deviation | 1,487,215.7 ± 1,305,406.6 | 1,858,739.5 ± 1,597,112.2 | |
| Median and [25%; 75%] | 1,025,848.5 [482,072.9; 1,946,021.3] | 1,539,301.8 [458,424.7; 3,355,963.6] | |
| **PE 40:6__[M+H]__5.7** | | | 0.314 |
| Mean ± Standard deviation | 211,499.5 ± 180,541.1 | 318,165.8 ± 315,797.2 | |
| Median and [25%; 75%] | 149,152.5 [78,199.5; 303,638.0] | 166,844.0 [103,078.7; 423,519.4] | |
| **PE O-40:6__[M+H]__6.06** | | | 0.025 |
| Mean ± Standard deviation | 92,284.3 ± 61,513.7 | 201,765.7 ± 205,259.1 | |
| Median and [25%; 75%] | 75,405.3 [48,596.5; 122,481.0] | 115,719.8 [69,694.1; 213,861.0] | |
| **PE P-36:2\|PE P-18:0_18:2__[M+H]__6.33** | | | 0.100 |
| Mean ± Standard deviation | 295,858.6 ± 309,518.8 | 790,677.5 ± 1,118,274.6 | |
| Median and [25%; 75%] | 207,759.0 [119,125.8; 356,505.9] | 269,502.0 [161,069.5; 836,745.9] | |
| **PE P-36:4\|PE P-16:0_20:4__[M+H]__5.65** | | | 0.093 |
| Mean ± Standard deviation | 465,359.0 ± 474,116.8 | 654,772.8 ± 526,636.3 | |
| Median and [25%; 75%] | 257,781.8 [148,305.8; 575,979.9] | 463,931.4 [319,678.9; 910,931.0] | |
| **PE P-38:6\|PE P-16:0_22:6__[M+H]__5.46** | | | 0.084 |
| Mean ± Standard deviation | 722,205.7 ± 454,998.4 | 959,596.5 ± 467,533.6 | |
| Median and [25%; 75%] | 641,360.0 [330,958.5; 995,339.0] | 918,516.8 [675,346.6; 1,217,450.1] | |
| **PE P-40:7\|PE P-18:1_22:6__[M+H]__5.52** | | | 0.097 |
| Mean ± Standard deviation | 197,739.4 ± 130,238.2 | 228,246.4 ± 90,388.9 | |
| Median and [25%; 75%] | 155,439.5 [113,854.0; 253,734.5] | 235,323.3 [181,369.4; 278,315.6] | |
| **PI 34:2__[M+NH4]__4.55** | | | 0.020 |
| Mean ± Standard deviation | 365,213.0 ± 254,676.2 | 540,191.9 ± 307,612.1 | |
| Median and [25%; 75%] | 318,749.8 [205,032.5; 459,391.3] | 477,518.5 [315,001.2; 640,610.0] | |
| **PI 34:2__[M+Na]__4.55** | | | <0.001 |
| Mean ± Standard deviation | 118,052.2 ± 107,531.4 | 300,827.9 ± 146,745.7 | |
| Median and [25%; 75%] | 85,174.5 [41,660.5; 196,806.3] | 281,918.5 [186,453.1; 386,882.8] | |
| **PI 36:2__[M+NH4]__4.98** | | | 0.022 |
| Mean ± Standard deviation | 244,570.9 ± 158,066.6 | 338,553.2 ± 146,251.6 | |
| Median and [25%; 75%] | 226,930.0 [133,280.5; 319,833.5] | 310,987.5 [271,024.9; 380,652.6] | |
| **PI 36:4__[M+NH4]__4.53** | | | 0.001 |

| | | | |
|---|---|---|---|
| Mean ± Standard deviation | 665,851.9 ± 392,749.0 | 1,029,868.0 ± 366,784.5 | |
| Median and [25%; 75%] | 587,042.5 [402,097.0; 852,656.8] | 965,500.0 [733,086.8; 1,199,188.6] | |
| **PI 36:4__[M+Na]__4.53** | | | <0.001 |
| Mean ± Standard deviation | 163,943.1 ± 98,580.4 | 408,542.3 ± 124,937.0 | |
| Median and [25%; 75%] | 149,064.5 [107,025.5; 199,975.1] | 372,613.5 [314,688.6; 490,804.5] | |
| **PI 38:4__[M+NH4]__4.96** | | | 0.135 |
| Mean ± Standard deviation | 5,704,427.9 ± 2,474,902.9 | 6,994,866.3 ± 2,972,312.1 | |
| Median and [25%; 75%] | 5,869,296.5 [3,946,990.5; 7,275,077.5] | 6,002,529.8 [4,864,677.3; 8,903,142.9] | |
| **SM 20:0;O3__[M+H]__2.56** | | | <0.001 |
| Mean ± Standard deviation | 1,213,476.3 ± 389,321.1 | 1,682,939.0 ± 528,551.5 | |
| Median and [25%; 75%] | 1,150,164.5 [946,696.8; 1,449,514.0] | 1,625,740.0 [1,306,923.9; 1,949,980.9] | |
| **SM 20:1;O3__[M+H]__2.2** | | | 0.036 |
| Mean ± Standard deviation | 6,588,416.1 ± 1,903,396.2 | 8,523,206.0 ± 3,683,019.3 | |
| Median and [25%; 75%] | 6,206,391.0 [5,129,339.3; 7,694,551.4] | 7,141,986.9 [6,006,808.0; 9,539,585.1] | |
| **SM 20:2;O3__[M+H]__1.78** | | | 0.022 |
| Mean ± Standard deviation | 8,394,454.7 ± 3,786,342.2 | 12,396,792.7 ± 6,880,223.5 | |
| Median and [25%; 75%] | 7,286,083.0 [6,274,615.8; 9,080,220.1] | 11,044,484.5 [7,445,130.9; 14,359,761.3] | |
| **SM 26:1;O3__[M+H]__6.68** | | | 0.041 |
| Mean ± Standard deviation | 25,650.5 ± 41,853.0 | 40,405.6 ± 53,535.6 | |
| Median and [25%; 75%] | 6,813.5 [3,610.4; 21,267.5] | 17,884.3 [9,639.1; 52,481.8] | |
| **SM 30:1;O2\|SM 18:1;O2/12:0__[M+H]__4.2** | | | <0.001 |
| Mean ± Standard deviation | 321,925.1 ± 281,148.1 | 756,592.6 ± 485,177.5 | |
| Median and [25%; 75%] | 254,295.0 [149,556.9; 376,978.8] | 573,987.3 [406,351.6; 1,088,790.9] | |
| **SM 30:3;O3__[M+H]__6.85** | | | 0.926 |
| Mean ± Standard deviation | 73,556.8 ± 84,654.2 | 59,939.9 ± 52,106.6 | |
| Median and [25%; 75%] | 36,139.5 [19,841.9; 105,255.8] | 49,761.3 [23,228.6; 76,047.8] | |
| **SM 31:1;O2\|SM 17:1;O2/14:0__[M+H]__4.39** | | | <0.001 |
| Mean ± Standard deviation | 195,792.0 ± 126,419.6 | 529,477.5 ± 344,740.8 | |
| Median and [25%; 75%] | 173,850.3 [103,271.3; 252,810.6] | 449,131.5 [260,573.9; 839,414.2] | |
| **SM 31:2;O3__[M+H]__4.6** | | | <0.001 |
| Mean ± Standard deviation | 1,165,707.7 ± 577,524.8 | 1,998,593.7 ± 610,730.0 | |
| Median and [25%; 75%] | 1,097,051.5 [684,237.5; 1,629,456.9] | 1,804,351.0 [1,581,402.0; 2,522,302.6] | |
| **SM 31:3;O3__[M+H]__4.25** | | | <0.001 |
| Mean ± Standard deviation | 57,247.1 ± 34,106.5 | 175,448.1 ± 254,305.6 | |
| Median and [25%; 75%] | 49,081.0 [35,137.8; 68,611.0] | 116,185.8 [85,761.0; 143,127.1] | |
| **SM 32:0;O2__[M+H]__4.77** | | | 0.132 |

| | | | |
|---|---|---|---|
| Mean ± Standard deviation | 210,700.3 ± 185,159.5 | 312,782.5 ± 308,512.6 | |
| Median and [25%; 75%] | 150,845.0 [80,119.3; 283,195.5] | 179,170.3 [140,048.9; 356,156.1] | |
| **SM 32:1;O2__[M+Na]__4.63** | | | 0.442 |
| Mean ± Standard deviation | 927,123.6 ± 620,180.4 | 852,240.3 ± 375,002.8 | |
| Median and [25%; 75%] | 687,497.8 [469,018.4; 1,254,789.4] | 739,650.0 [693,503.5; 1,004,731.5] | |
| **SM 32:1;O2\|SM 16:1;O2/16:0__[M+H]__4.6** | | | <0.001 |
| Mean ± Standard deviation | 29,527,031.4 ± 8,546,009.4 | 50,909,124.9 ± 13,868,581.6 | |
| Median and [25%; 75%] | 29,844,793.0 [23,768,764.0; 33,146,093 | 48,921,465.0 [43,190,066.0; 58,419,318.5] | |
| **SM 32:2;O2__[M+H]__4.27** | | | <0.001 |
| Mean ± Standard deviation | 1,179,716.5 ± 621,098.5 | 2,136,545.4 ± 764,568.7 | |
| Median and [25%; 75%] | 1,094,002.8 [722,862.3; 1,276,273.9] | 1,918,730.8 [1,544,053.4; 2,826,460.8] | |
| **SM 32:2;O2\|SM 18:2;O2/14:0__[M+H]__4.26** | | | <0.001 |
| Mean ± Standard deviation | 1,179,540.6 ± 620,816.0 | 2,136,473.1 ± 764,477.7 | |
| Median and [25%; 75%] | 1,094,002.8 [722,862.3; 1,276,273.9] | 1,918,730.8 [1,544,053.4; 2,826,460.8] | |
| **SM 33:1;O2__[M+Na]__4.82** | | | 0.387 |
| Mean ± Standard deviation | 746,119.0 ± 363,397.7 | 672,873.7 ± 250,964.9 | |
| Median and [25%; 75%] | 713,720.8 [516,107.8; 978,040.0] | 591,080.0 [489,280.5; 875,613.8] | |
| **SM 33:1;O2\|SM 17:1;O2/16:0__[M+H]__4.83** | | | <0.001 |
| Mean ± Standard deviation | 13,203,978.7 ± 4,711,851.6 | 21,257,462.0 ± 8,336,327.6 | |
| Median and [25%; 75%] | 13,128,103.0 [9,857,954.6; 15,409,181.3 | 20,103,642.5 [14,032,910.8; 25,696,184.3] | |
| **SM 33:1;O3__[M+H]__5.09** | | | 0.442 |
| Mean ± Standard deviation | 2,921,202.3 ± 1,327,234.5 | 3,427,557.4 ± 1,490,729.1 | |
| Median and [25%; 75%] | 2,935,515.0 [1,782,050.0; 3,404,163.5] | 2,882,999.5 [2,361,029.9; 4,344,786.4] | |
| **SM 33:2;O2\|SM 17:2;O2/16:0__[M+H]__4.5** | | | 0.023 |
| Mean ± Standard deviation | 275,138.5 ± 185,976.7 | 403,342.8 ± 242,569.3 | |
| Median and [25%; 75%] | 229,193.0 [157,070.9; 366,243.9] | 306,009.3 [241,661.9; 559,460.0] | |
| **SM 34:0;O2__[M+H]__5.25** | | | 0.294 |
| Mean ± Standard deviation | 13,395,490.9 ± 3,507,697.6 | 14,750,288.0 ± 4,561,101.7 | |
| Median and [25%; 75%] | 13,514,143.5 [10,425,597.8; 15,193,250 | 13,654,102.8 [12,281,574.5; 17,119,778.3] | |
| **SM 34:1;O2__[M+Na]__5.12** | | | <0.001 |
| Mean ± Standard deviation | 552,769.2 ± 296,598.0 | 314,863.3 ± 156,457.7 | |
| Median and [25%; 75%] | 505,967.0 [363,453.3; 632,657.3] | 273,706.8 [183,621.4; 401,094.0] | |
| **SM 34:1;O2\|SM 18:1;O2/16:0__[M+H]__5.08** | | | 0.007 |
| Mean ± Standard deviation | 254,899,440.2 ± 43,171,854.7 | 293,122,135.8 ± 45,182,069.4 | |
| Median and [25%; 75%] | 257,879,208.0 [230,664,632.0; 285,147, | 299,577,528.0 [262,759,978.0; 327,315,035.0] | |
| **SM 34:1;O3__[M+H]__4.92** | | | 0.993 |

| | | | |
|---|---|---|---|
| Mean ± Standard deviation | 193,276.2 ± 119,978.6 | 190,455.5 ± 111,362.6 | |
| Median and [25%; 75%] | 159,244.0 [95,034.8; 262,003.6] | 178,918.3 [98,737.8; 256,280.4] | |
| **SM 34:2;O2__[M+Na]__4.67** | | | 0.212 |
| Mean ± Standard deviation | 3,036,625.1 ± 609,645.6 | 3,230,540.8 ± 626,718.9 | |
| Median and [25%; 75%] | 2,871,116.0 [2,596,332.6; 3,412,655.8] | 3,247,696.0 [2,748,715.9; 3,683,136.4] | |
| **SM 34:2;O2\|SM 18:2;O2/16:0__[M+H]__4.68** | | | <0.001 |
| Mean ± Standard deviation | 42,813,870.4 ± 9,127,894.4 | 56,794,874.2 ± 14,174,832.6 | |
| Median and [25%; 75%] | 42,728,190.0 [38,160,530.0; 50,230,569 | 54,798,103.0 [46,198,820.0; 63,582,354.5] | |
| **SM 34:2;O3__[M+H]__4.52** | | | 0.115 |
| Mean ± Standard deviation | 513,963.0 ± 190,629.3 | 646,013.8 ± 263,308.9 | |
| Median and [25%; 75%] | 474,177.0 [384,901.6; 614,532.0] | 603,119.8 [396,156.4; 866,390.9] | |
| **SM 35:1;O2__[M+H]__5.34** | | | 0.061 |
| Mean ± Standard deviation | 1,805,922.7 ± 934,096.2 | 2,201,718.6 ± 862,501.6 | |
| Median and [25%; 75%] | 1,617,690.5 [1,168,902.0; 2,007,778.0] | 1,913,321.0 [1,657,056.6; 2,739,865.0] | |
| **SM 35:1;O2\|SM 18:1;O2/17:0__[M+H]__5.35** | | | 0.059 |
| Mean ± Standard deviation | 1,787,486.8 ± 948,675.3 | 2,201,420.6 ± 860,971.0 | |
| Median and [25%; 75%] | 1,607,250.5 [1,167,205.8; 2,002,431.8] | 1,916,328.8 [1,656,038.1; 2,739,697.0] | |
| **SM 35:1;O3__[M+H]__5.62** | | | <0.001 |
| Mean ± Standard deviation | 1,179,925.8 ± 1,355,618.7 | 3,306,948.3 ± 1,986,883.6 | |
| Median and [25%; 75%] | 724,274.5 [281,885.0; 1,476,455.3] | 2,575,595.0 [2,072,448.1; 4,149,105.0] | |
| **SM 35:2;O3__[M+H]__5.62** | | | 0.169 |
| Mean ± Standard deviation | 2,590,027.6 ± 1,270,632.8 | 3,107,556.3 ± 1,242,608.6 | |
| Median and [25%; 75%] | 2,166,385.5 [1,734,092.8; 3,638,762.0] | 2,894,598.4 [2,301,032.3; 4,289,368.6] | |
| **SM 35:3;O3__[M+H]__5.17** | | | 0.022 |
| Mean ± Standard deviation | 930,072.6 ± 462,572.2 | 1,316,856.5 ± 604,074.1 | |
| Median and [25%; 75%] | 923,600.0 [568,192.3; 1,178,538.8] | 1,143,325.8 [888,965.1; 1,564,954.4] | |
| **SM 35:4;O3__[M+H]__4.82** | | | 0.348 |
| Mean ± Standard deviation | 109,089.9 ± 49,612.4 | 99,821.2 ± 39,833.3 | |
| Median and [25%; 75%] | 107,480.0 [73,317.8; 140,167.8] | 90,407.0 [68,925.8; 113,179.8] | |
| **SM 36:0;O2__[M+H]__5.84** | | | 0.442 |
| Mean ± Standard deviation | 317,912.7 ± 451,906.3 | 190,393.7 ± 206,737.8 | |
| Median and [25%; 75%] | 135,890.5 [65,623.0; 339,164.0] | 118,763.0 [67,962.4; 203,499.9] | |
| **SM 36:1;O2\|SM 18:1;O2/18:0__[M+H]__5.62** | | | 0.084 |
| Mean ± Standard deviation | 42,399,680.8 ± 11,012,612.2 | 49,004,828.4 ± 13,027,019.8 | |
| Median and [25%; 75%] | 42,382,890.0 [35,171,925.3; 50,010,417 | 49,014,129.5 [40,963,688.0; 58,344,570.5] | |
| **SM 36:1;O3__[M+H]__6.04** | | | 0.078 |

| | | | |
|---|---|---|---|
| Mean ± Standard deviation | 3,480,453.5 ± 1,541,789.4 | 4,528,731.1 ± 2,001,870.7 | |
| Median and [25%; 75%] | 3,124,527.0 [2,517,314.3; 4,428,614.4] | 4,476,704.5 [3,353,371.9; 4,867,891.4] | |
| **SM 36:2;O2\|SM 18:2;O2/18:0__[M+H]__5.17** | | | 0.060 |
| Mean ± Standard deviation | 15,750,092.5 ± 5,542,220.8 | 19,809,866.7 ± 7,434,680.2 | |
| Median and [25%; 75%] | 15,234,496.8 [12,267,756.3; 19,933,576 | 18,634,690.8 [13,954,133.1; 23,638,223.5] | |
| **SM 36:2;O3__[M+H]__5.93** | | | 0.002 |
| Mean ± Standard deviation | 1,713,542.5 ± 1,366,771.8 | 3,248,560.2 ± 2,038,438.6 | |
| Median and [25%; 75%] | 1,439,275.0 [990,383.6; 1,827,714.0] | 2,555,484.0 [1,509,988.1; 4,128,210.8] | |
| **SM 36:3;O3__[M+H]__5.55** | | | 0.002 |
| Mean ± Standard deviation | 3,167,428.4 ± 1,681,074.5 | 6,851,200.7 ± 5,859,524.4 | |
| Median and [25%; 75%] | 2,799,612.0 [2,169,123.8; 3,782,544.5] | 4,245,316.6 [3,235,168.4; 8,853,465.3] | |
| **SM 37:0;O3__[M+H]__5.66** | | | <0.001 |
| Mean ± Standard deviation | 228,068.7 ± 213,472.6 | 320,875.8 ± 143,730.1 | |
| Median and [25%; 75%] | 164,511.5 [126,125.1; 233,143.3] | 274,251.8 [215,016.5; 374,514.5] | |
| **SM 37:1;O2\|SM 21:1;O2/16:0__[M+H]__5.92** | | | 0.097 |
| Mean ± Standard deviation | 2,348,366.5 ± 1,229,103.3 | 3,145,874.3 ± 1,844,993.7 | |
| Median and [25%; 75%] | 2,063,504.3 [1,509,895.9; 2,935,839.0] | 2,790,620.0 [2,098,211.9; 3,725,434.3] | |
| **SM 37:1;O3__[M+H]__5.67** | | | <0.001 |
| Mean ± Standard deviation | 185,943,716.4 ± 58,241,191.9 | 264,873,838.8 ± 99,954,592.7 | |
| Median and [25%; 75%] | 181,823,848.0 [151,007,448.0; 214,676, | 245,256,144.0 [210,704,228.0; 300,642,120.0] | |
| **SM 37:3;O3__[M+H]__5.75** | | | 0.537 |
| Mean ± Standard deviation | 232,589.5 ± 150,832.3 | 219,348.7 ± 173,845.4 | |
| Median and [25%; 75%] | 197,657.5 [133,891.3; 267,197.9] | 183,855.5 [94,804.5; 256,031.6] | |
| **SM 37:5;O3__[M+H]__5.41** | | | 0.055 |
| Mean ± Standard deviation | 996,422.8 ± 879,614.9 | 1,372,865.5 ± 958,561.0 | |
| Median and [25%; 75%] | 688,669.0 [419,525.8; 1,161,989.5] | 1,062,157.3 [784,399.8; 1,689,779.9] | |
| **SM 38:0;O2__[M+H]__6.33** | | | 0.059 |
| Mean ± Standard deviation | 406,830.5 ± 170,299.1 | 643,976.9 ± 425,460.7 | |
| Median and [25%; 75%] | 405,848.0 [292,313.5; 506,069.6] | 496,195.0 [348,577.1; 793,763.4] | |
| **SM 38:1;O3__[M+H]__6.0** | | | 0.013 |
| Mean ± Standard deviation | 457,596.2 ± 272,868.4 | 696,970.5 ± 446,158.6 | |
| Median and [25%; 75%] | 409,849.5 [295,549.6; 561,487.8] | 633,947.0 [453,847.6; 832,508.3] | |
| **SM 38:2;O2\|SM 18:2;O2/20:0__[M+H]__5.74** | | | 0.087 |
| Mean ± Standard deviation | 4,057,195.9 ± 1,458,971.2 | 3,392,512.9 ± 1,388,475.8 | |
| Median and [25%; 75%] | 3,759,030.8 [2,966,097.8; 4,841,620.6] | 3,216,870.5 [2,616,284.3; 4,055,330.9] | |
| **SM 38:2;O3__[M+H]__5.5** | | | <0.001 |

| | | | |
|---|---|---|---|
| Mean ± Standard deviation | 2,612,399.6 ± 1,192,420.5 | 5,373,971.5 ± 2,802,381.3 | |
| Median and [25%; 75%] | 2,411,006.5 [1,985,963.0; 3,055,418.5] | 4,584,584.8 [3,044,967.0; 6,940,683.6] | |
| **SM 38:3;O3__[M+H]__6.12** | | | 0.002 |
| Mean ± Standard deviation | 1,336,367.9 ± 638,608.2 | 2,636,731.6 ± 1,759,504.6 | |
| Median and [25%; 75%] | 1,196,930.0 [802,219.5; 1,892,752.3] | 1,861,613.8 [1,525,036.5; 3,367,404.3] | |
| **SM 38:4;O2__[M+H]__5.4** | | | 0.041 |
| Mean ± Standard deviation | 2,068,743.5 ± 825,913.9 | 1,708,613.1 ± 465,644.1 | |
| Median and [25%; 75%] | 2,179,987.8 [1,466,355.3; 2,596,172.6] | 1,693,685.0 [1,417,140.7; 1,998,214.5] | |
| **SM 38:4;O3__[M+H]__5.55** | | | 0.033 |
| Mean ± Standard deviation | 9,810,209.0 ± 4,787,322.9 | 11,919,226.0 ± 4,328,202.9 | |
| Median and [25%; 75%] | 8,532,309.0 [6,813,504.3; 11,720,988.0] | 11,545,382.8 [9,233,654.9; 14,441,460.1] | |
| **SM 38:5;O3__[M+H]__5.16** | | | 0.742 |
| Mean ± Standard deviation | 3,290,916.2 ± 5,608,441.5 | 843,215.8 ± 3,012,238.9 | |
| Median and [25%; 75%] | 38,617.0 [19,137.1; 7,416,856.0] | 40,924.8 [29,402.8; 91,322.8] | |
| **SM 38:6;O3__[M+H]__5.16** | | | 0.041 |
| Mean ± Standard deviation | 109,604.2 ± 104,255.8 | 190,910.4 ± 170,059.3 | |
| Median and [25%; 75%] | 80,391.0 [40,193.4; 150,242.8] | 133,077.3 [77,467.8; 230,467.1] | |
| **SM 38:7;O3__[M+H]__6.12** | | | <0.001 |
| Mean ± Standard deviation | 1,637.0 ± 1,147.2 | 3,070.0 ± 1,371.9 | |
| Median and [25%; 75%] | 1,467.5 [826.5; 1,906.8] | 2,693.0 [1,993.6; 3,986.3] | |
| **SM 39:0;O2__[M+H]__6.69** | | | <0.001 |
| Mean ± Standard deviation | 634,512.2 ± 262,584.2 | 1,266,582.3 ± 596,824.9 | |
| Median and [25%; 75%] | 588,854.0 [440,486.9; 756,226.8] | 1,136,786.3 [798,205.1; 1,638,541.1] | |
| **SM 39:1;O2__[M+H]__6.17** | | | 0.080 |
| Mean ± Standard deviation | 314,501.6 ± 228,839.0 | 566,141.8 ± 509,587.2 | |
| Median and [25%; 75%] | 249,531.0 [170,115.3; 384,187.9] | 470,766.3 [213,305.0; 653,939.1] | |
| **SM 39:1;O2\|SM 16:1;O2/23:0__[M+H]__6.67** | | | <0.001 |
| Mean ± Standard deviation | 12,967,136.9 ± 4,616,928.7 | 22,412,221.3 ± 9,690,495.2 | |
| Median and [25%; 75%] | 12,804,240.0 [9,003,652.5; 15,303,149.9 | 20,021,757.0 [15,520,733.4; 28,645,105.9] | |
| **SM 39:1;O3 OA1__[M+H]__5.81** | | | <0.001 |
| Mean ± Standard deviation | 238,403.0 ± 113,805.3 | 426,866.1 ± 193,946.0 | |
| Median and [25%; 75%] | 221,370.0 [161,257.6; 299,231.1] | 426,793.5 [284,501.2; 530,466.0] | |
| **SM 39:1;O3 OA2__[M+H]__6.96** | | | <0.001 |
| Mean ± Standard deviation | 24,964,175.1 ± 11,371,725.8 | 63,659,198.4 ± 68,301,256.2 | |
| Median and [25%; 75%] | 23,293,443.0 [17,047,558.8; 27,906,448 | 52,371,159.0 [28,963,229.8; 64,018,176.3] | |
| **SM 39:1;O3__[M+H]__6.26** | | | <0.001 |

| | | | |
|---|---|---|---|
| Mean ± Standard deviation | 26,203,221.4 ± 11,034,976.3 | 63,627,143.9 ± 68,268,449.2 | |
| Median and [25%; 75%] | 24,407,255.0 [17,812,025.0; 29,335,594 | 52,336,158.0 [28,946,188.6; 63,989,758.0] | |
| **SM 39:2;O2__[M+H]__6.1** | | | 0.009 |
| Mean ± Standard deviation | 1,611,926.4 ± 625,346.2 | 2,658,567.9 ± 1,559,162.9 | |
| Median and [25%; 75%] | 1,619,947.0 [1,196,789.8; 2,018,823.4] | 2,202,130.8 [1,611,420.6; 3,683,188.4] | |
| **SM 39:3;O3__[M+H]__5.38** | | | <0.001 |
| Mean ± Standard deviation | 99,805,332.5 ± 39,353,041.5 | 166,734,285.1 ± 46,025,263.7 | |
| Median and [25%; 75%] | 95,015,412.0 [71,572,352.0; 125,841,72 | 165,268,656.0 [131,531,882.0; 192,977,300.0] | |
| **SM 39:6;O2__[M+H]__5.67** | | | <0.001 |
| Mean ± Standard deviation | 1,444,027.5 ± 564,979.5 | 2,466,766.5 ± 914,584.3 | |
| Median and [25%; 75%] | 1,491,969.5 [986,138.9; 1,838,014.8] | 2,196,490.3 [1,736,842.1; 3,035,784.0] | |
| **SM 39:8;O3__[M+H]__5.91** | | | 0.504 |
| Mean ± Standard deviation | 30,004.5 ± 18,269.9 | 31,545.4 ± 15,622.1 | |
| Median and [25%; 75%] | 26,833.5 [17,206.0; 38,670.0] | 28,710.8 [23,146.8; 39,956.9] | |
| **SM 39:9;O2__[M+H]__4.89** | | | <0.001 |
| Mean ± Standard deviation | 990,728.2 ± 712,664.2 | 2,601,205.3 ± 1,427,069.9 | |
| Median and [25%; 75%] | 813,566.0 [477,366.5; 1,400,817.0] | 2,305,304.8 [1,619,174.3; 3,091,284.3] | |
| **SM 40:0;O2__[M+H]__6.96** | | | <0.001 |
| Mean ± Standard deviation | 3,253,629.2 ± 1,287,333.0 | 5,569,803.3 ± 2,456,901.9 | |
| Median and [25%; 75%] | 2,905,528.5 [2,320,932.0; 3,884,317.0] | 4,906,514.0 [4,057,608.0; 6,728,057.3] | |
| **SM 40:1;O2__[M+H]__6.34** | | | <0.001 |
| Mean ± Standard deviation | 2,819,796.7 ± 1,035,941.1 | 4,283,689.5 ± 1,438,942.0 | |
| Median and [25%; 75%] | 2,997,664.3 [1,984,348.0; 3,289,041.0] | 4,251,268.5 [3,432,844.7; 5,047,426.8] | |
| **SM 40:2;O2\|SM 18:2;O2/22:0__[M+H]__6.4** | | | <0.001 |
| Mean ± Standard deviation | 66,747,270.9 ± 14,997,889.8 | 93,249,803.1 ± 21,071,097.1 | |
| Median and [25%; 75%] | 66,993,604.0 [57,498,148.0; 76,036,844 | 94,327,244.0 [79,595,968.0; 102,953,107.0] | |
| **SM 40:2;O3 OA1__[M+H]__6.76** | | | <0.001 |
| Mean ± Standard deviation | 439,603.7 ± 204,967.5 | 758,028.8 ± 258,063.4 | |
| Median and [25%; 75%] | 433,069.5 [297,657.5; 560,964.0] | 715,777.3 [590,066.2; 846,084.9] | |
| **SM 40:2;O3__[M+H]__6.09** | | | <0.001 |
| Mean ± Standard deviation | 110,716.7 ± 69,740.4 | 345,062.9 ± 266,953.3 | |
| Median and [25%; 75%] | 98,700.5 [69,025.3; 128,627.1] | 260,532.5 [140,351.1; 450,523.0] | |
| **SM 40:4;O3 OA1__[M+H]__5.48** | | | 0.886 |
| Mean ± Standard deviation | 1,437,008.2 ± 1,395,142.5 | 1,313,937.9 ± 832,692.1 | |
| Median and [25%; 75%] | 956,143.0 [607,045.5; 1,592,722.6] | 1,211,963.1 [580,145.4; 2,068,931.1] | |
| **SM 40:4;O3__[M+H]__6.15** | | | 0.003 |

| | | | |
|---|---|---|---|
| Mean ± Standard deviation | 7,435,303.8 ± 2,666,046.7 | 10,521,647.8 ± 4,166,671.1 | |
| Median and [25%; 75%] | 7,217,689.0 [5,463,035.0; 8,835,245.4] | 10,959,332.5 [8,858,403.4; 11,745,094.0] | |
| **SM 40:5;O3__[M+H]__5.63** | | | 0.630 |
| Mean ± Standard deviation | 5,412,951.9 ± 3,175,064.6 | 5,129,037.6 ± 1,981,388.9 | |
| Median and [25%; 75%] | 4,488,478.5 [3,346,343.1; 5,992,375.3] | 4,991,955.9 [3,996,555.4; 6,472,451.5] | |
| **SM 40:6;O3__[M+H]__5.36** | | | 0.606 |
| Mean ± Standard deviation | 392,923.3 ± 277,971.1 | 470,414.0 ± 459,152.6 | |
| Median and [25%; 75%] | 326,768.0 [167,722.3; 575,434.8] | 364,439.0 [221,687.4; 442,406.7] | |
| **SM 40:8;O2__[M+H]__5.5** | | | <0.001 |
| Mean ± Standard deviation | 1,571,789.7 ± 735,496.5 | 3,183,508.9 ± 1,902,087.2 | |
| Median and [25%; 75%] | 1,532,538.3 [1,058,440.3; 2,101,902.1] | 2,644,514.5 [2,106,184.3; 3,624,388.4] | |
| **SM 40:8;O3__[M+H]__6.28** | | | 0.001 |
| Mean ± Standard deviation | 19,078.5 ± 13,009.0 | 33,744.9 ± 19,642.9 | |
| Median and [25%; 75%] | 14,478.5 [8,974.3; 26,967.8] | 28,790.0 [20,257.6; 44,684.4] | |
| **SM 41:0;O2__[M+H]__7.34** | | | <0.001 |
| Mean ± Standard deviation | 1,524,932.8 ± 444,466.9 | 2,584,468.8 ± 887,497.9 | |
| Median and [25%; 75%] | 1,543,226.5 [1,169,030.5; 1,893,248.0] | 2,542,146.0 [1,921,745.1; 3,092,534.8] | |
| **SM 41:1;O2\|SM 18:1;O2/23:0__[M+H]__7.34** | | | <0.001 |
| Mean ± Standard deviation | 36,088,916.8 ± 10,579,995.8 | 58,788,414.2 ± 20,672,869.5 | |
| Median and [25%; 75%] | 36,981,248.0 [26,899,687.3; 42,790,673 | 51,388,561.0 [42,130,418.8; 78,611,765.0] | |
| **SM 41:1;O3__[M+H]__7.72** | | | <0.001 |
| Mean ± Standard deviation | 812,284.1 ± 424,317.5 | 1,400,696.9 ± 728,263.5 | |
| Median and [25%; 75%] | 752,421.0 [562,668.0; 1,038,718.1] | 1,287,506.3 [831,918.3; 1,527,538.1] | |
| **SM 41:2;O2\|SM 17:1;O2/24:1__[M+H]__6.61** | | | 0.002 |
| Mean ± Standard deviation | 12,496,994.0 ± 4,222,508.8 | 16,797,244.6 ± 4,440,465.2 | |
| Median and [25%; 75%] | 11,676,689.0 [9,225,322.0; 15,283,602.0 | 16,643,813.5 [13,067,464.6; 18,519,346.3] | |
| **SM 41:2;O2\|SM 18:2;O2/23:0__[M+H]__6.77** | | | <0.001 |
| Mean ± Standard deviation | 19,331,495.4 ± 5,986,233.8 | 31,895,597.2 ± 11,320,133.9 | |
| Median and [25%; 75%] | 19,420,010.0 [15,269,673.0; 22,451,566 | 28,812,158.0 [25,754,829.8; 36,169,190.8] | |
| **SM 41:2;O3 OA1__[M+H]__6.96** | | | 0.021 |
| Mean ± Standard deviation | 3,110,836.9 ± 1,402,066.6 | 3,968,818.6 ± 1,130,925.9 | |
| Median and [25%; 75%] | 2,643,095.5 [2,069,404.8; 4,239,589.3] | 3,682,500.0 [3,305,969.8; 4,315,310.1] | |
| **SM 41:2;O3__[M+H]__7.73** | | | 0.001 |
| Mean ± Standard deviation | 6,156,327.4 ± 3,229,917.4 | 9,316,157.9 ± 3,272,875.9 | |
| Median and [25%; 75%] | 5,641,391.0 [4,588,905.8; 7,871,738.9] | 8,907,130.0 [6,947,565.9; 11,442,655.6] | |
| **SM 41:3;O2__[M+H]__6.06** | | | 0.001 |

| | | | |
|---|---|---|---|
| Mean ± Standard deviation | 5,588,870.1 ± 2,621,872.0 | 8,671,137.7 ± 3,027,772.1 | |
| Median and [25%; 75%] | 5,221,976.0 [3,680,448.0; 6,876,836.5] | 8,459,090.3 [6,817,264.9; 10,635,317.9] | |
| **SM 41:3;O3__[M+H]__5.94** | | | 0.003 |
| Mean ± Standard deviation | 34,509,757.6 ± 20,536,195.3 | 53,043,014.4 ± 26,613,245.0 | |
| Median and [25%; 75%] | 30,248,035.0 [18,994,939.0; 41,870,688 | 46,006,211.0 [36,189,727.3; 61,923,946.5] | |
| **SM 41:4;O3__[M+H]__5.75** | | | 0.846 |
| Mean ± Standard deviation | 112,326,016.1 ± 64,161,217.9 | 105,434,250.4 ± 55,852,641.7 | |
| Median and [25%; 75%] | 92,515,120.0 [65,698,995.0; 130,992,42 | 100,958,244.0 [65,459,433.0; 157,746,856.0] | |
| **SM 41:5;O3 OA1__[M+H]__5.34** | | | 0.080 |
| Mean ± Standard deviation | 11,679,298.3 ± 10,535,929.4 | 14,176,579.6 ± 7,268,311.8 | |
| Median and [25%; 75%] | 7,645,484.5 [4,794,393.3; 13,257,742.8] | 14,101,207.5 [6,850,930.3; 21,381,241.3] | |
| **SM 41:5;O3__[M+H]__5.18** | | | 0.002 |
| Mean ± Standard deviation | 4,537,074.4 ± 2,209,963.6 | 6,935,188.7 ± 2,979,076.6 | |
| Median and [25%; 75%] | 3,887,931.0 [2,890,689.8; 5,534,522.0] | 6,189,574.0 [4,820,524.8; 8,469,103.5] | |
| **SM 41:7;O2__[M+H]__5.81** | | | <0.001 |
| Mean ± Standard deviation | 1,507,471.8 ± 712,543.0 | 2,721,702.9 ± 1,450,998.1 | |
| Median and [25%; 75%] | 1,451,955.0 [1,037,510.3; 1,838,995.8] | 2,227,919.3 [1,675,118.1; 3,401,881.5] | |
| **SM 41:8;O2__[M+H]__5.38** | | | <0.001 |
| Mean ± Standard deviation | 675,392.7 ± 350,616.3 | 1,465,905.7 ± 513,831.1 | |
| Median and [25%; 75%] | 635,558.0 [359,894.4; 877,588.8] | 1,269,715.6 [1,093,716.6; 1,899,640.4] | |
| **SM 42:0;O2\|SM 18:0;O2/24:0__[M+H]__7.72** | | | <0.001 |
| Mean ± Standard deviation | 2,999,515.7 ± 972,608.5 | 4,566,128.2 ± 1,819,957.2 | |
| Median and [25%; 75%] | 2,897,405.5 [2,309,715.5; 3,561,107.3] | 3,813,389.8 [3,278,080.3; 5,393,125.1] | |
| **SM 42:1;O2__[M+H]__7.72** | | | <0.001 |
| Mean ± Standard deviation | 63,825,763.3 ± 19,506,178.0 | 95,242,261.7 ± 32,895,236.0 | |
| Median and [25%; 75%] | 64,807,442.0 [48,170,230.0; 75,409,876 | 93,296,114.0 [69,519,622.0; 110,559,075.0] | |
| **SM 42:2;O2\|SM 18:2;O2/24:0__[M+H]__7.15** | | | <0.001 |
| Mean ± Standard deviation | 41,685,869.7 ± 13,744,955.4 | 56,321,874.1 ± 13,067,482.4 | |
| Median and [25%; 75%] | 42,333,530.0 [33,653,995.0; 50,345,720 | 61,479,424.0 [47,073,022.5; 62,883,622.5] | |
| **SM 42:3;O2\|SM 18:2;O2/24:1__[M+H]__6.38** | | | 0.027 |
| Mean ± Standard deviation | 108,226,340.5 ± 24,617,319.5 | 127,872,871.9 ± 29,646,068.6 | |
| Median and [25%; 75%] | 108,011,176.0 [87,499,782.0; 123,680,0 | 119,899,500.0 [105,077,593.5; 145,767,586.0] | |
| **SM 42:4;O2__[M+H]__5.86** | | | 0.043 |
| Mean ± Standard deviation | 4,952,965.2 ± 2,271,716.4 | 3,951,488.1 ± 2,504,004.9 | |
| Median and [25%; 75%] | 4,591,045.0 [3,342,167.0; 6,422,705.3] | 3,246,642.4 [2,778,269.8; 4,147,813.3] | |
| **SM 42:4;O2\|SM 18:2;O2/24:2__[M+H]__5.86** | | | 0.044 |

| | | | |
|---|---|---|---|
| Mean ± Standard deviation | 4,957,913.0 ± 2,284,479.9 | 3,953,501.1 ± 2,510,482.3 | |
| Median and [25%; 75%] | 4,591,045.0 [3,342,167.0; 6,422,705.3] | 3,246,642.4 [2,778,269.8; 4,147,813.3] | |
| **SM 42:4;O3__[M+H]__6.83** | | | <0.001 |
| Mean ± Standard deviation | 612,247.5 ± 256,567.7 | 1,016,161.3 ± 526,344.5 | |
| Median and [25%; 75%] | 582,628.5 [449,926.0; 790,128.8] | 973,645.8 [808,417.1; 1,175,092.6] | |
| **SM 42:6;O3__[M+H]__5.91** | | | 0.899 |
| Mean ± Standard deviation | 377,739.0 ± 253,614.9 | 374,331.1 ± 229,408.9 | |
| Median and [25%; 75%] | 346,387.3 [165,511.0; 478,876.8] | 284,513.6 [233,937.4; 474,944.6] | |
| **SM 42:7;O3__[M+H]__5.45** | | | 0.899 |
| Mean ± Standard deviation | 354,137.0 ± 275,376.7 | 314,342.5 ± 237,963.0 | |
| Median and [25%; 75%] | 260,091.5 [137,302.3; 538,765.1] | 229,091.8 [189,542.4; 325,443.1] | |
| **SM 43:2;O2__[M+H]__7.2** | | | 0.015 |
| Mean ± Standard deviation | 3,469,205.5 ± 2,244,829.4 | 4,931,015.8 ± 1,934,465.2 | |
| Median and [25%; 75%] | 2,935,702.0 [1,738,134.8; 4,831,590.5] | 4,965,501.5 [4,106,246.4; 6,314,240.1] | |
| **SM 43:2;O2\|SM 19:1;O2/24:1__[M+H]__7.2** | | | 0.015 |
| Mean ± Standard deviation | 3,496,423.1 ± 2,241,529.5 | 4,938,354.3 ± 1,941,839.1 | |
| Median and [25%; 75%] | 2,935,702.0 [1,738,134.8; 4,831,590.5] | 4,965,501.5 [4,106,246.4; 6,314,240.1] | |
| **SM 43:4;O3__[M+H]__6.12** | | | 0.034 |
| Mean ± Standard deviation | 1,476,185.4 ± 1,228,139.2 | 2,385,137.7 ± 1,757,967.6 | |
| Median and [25%; 75%] | 996,806.5 [655,686.5; 1,779,172.0] | 2,305,679.5 [1,081,744.4; 2,829,143.3] | |
| **SM 43:5;O3__[M+H]__5.71** | | | 0.006 |
| Mean ± Standard deviation | 403,036.0 ± 319,963.8 | 652,248.4 ± 395,451.1 | |
| Median and [25%; 75%] | 303,094.0 [207,804.0; 437,299.0] | 485,957.3 [400,772.8; 994,116.5] | |
| **SM 43:6;O3__[M+H]__5.57** | | | 0.188 |
| Mean ± Standard deviation | 31,675,051.7 ± 17,752,761.4 | 40,048,801.8 ± 22,315,708.5 | |
| Median and [25%; 75%] | 25,601,867.0 [18,106,586.5; 40,329,791 | 36,386,294.0 [29,208,173.8; 49,808,975.0] | |
| **SM 43:7;O3__[M+H]__5.15** | | | 0.219 |
| Mean ± Standard deviation | 735,381.3 ± 689,899.2 | 1,017,835.0 ± 1,017,205.3 | |
| Median and [25%; 75%] | 520,806.0 [284,414.8; 923,726.0] | 864,583.5 [370,279.4; 1,192,425.6] | |
| **SM 43:9;O2__[M+H]__5.95** | | | <0.001 |
| Mean ± Standard deviation | 11,346,577.6 ± 5,401,544.7 | 18,396,034.6 ± 6,306,888.9 | |
| Median and [25%; 75%] | 10,099,151.0 [7,772,762.1; 15,541,908.3 | 16,096,055.5 [13,817,598.9; 23,156,500.3] | |
| **SM 44:5;O3__[M+H]__6.85** | | | 0.024 |
| Mean ± Standard deviation | 1,134,218.2 ± 506,699.5 | 1,644,382.2 ± 878,306.5 | |
| Median and [25%; 75%] | 1,083,841.5 [768,411.4; 1,391,336.4] | 1,646,145.0 [1,024,636.0; 1,995,097.4] | |
| **SM 44:6;O3__[M+H]__6.23** | | | 0.717 |

| | | | |
|---|---|---|---|
| Mean ± Standard deviation | 152,352.9 ± 109,119.6 | 161,303.9 ± 161,075.5 | |
| Median and [25%; 75%] | 137,300.3 [66,732.5; 202,573.0] | 115,710.3 [61,208.6; 177,028.5] | |
| **SM 46:6;O3__[M+H]__6.87** | | | 0.781 |
| Mean ± Standard deviation | 534,706.7 ± 494,392.6 | 571,081.0 ± 645,714.0 | |
| Median and [25%; 75%] | 316,192.0 [191,502.3; 757,084.5] | 375,832.0 [250,894.3; 451,761.6] | |
| **SM 47:4;O2__[M+H]__7.53** | | | 0.008 |
| Mean ± Standard deviation | 1,247,163.4 ± 621,454.8 | 1,919,282.5 ± 1,020,436.3 | |
| Median and [25%; 75%] | 1,145,900.5 [855,714.0; 1,391,484.6] | 1,945,502.0 [1,217,252.9; 2,262,809.1] | |
| **ST 27:1;O OA1__[M+H-H2O]__4.56** | | | 0.643 |
| Mean ± Standard deviation | 40,338,699.8 ± 12,610,448.2 | 38,660,908.5 ± 12,101,228.2 | |
| Median and [25%; 75%] | 39,059,964.0 [32,439,022.5; 49,002,896 | 35,923,528.8 [32,273,526.8; 42,131,238.0] | |
| **ST 27:1;O OA2__[M+H-H2O]__12.61** | | | 0.210 |
| Mean ± Standard deviation | 99,333,354.5 ± 17,645,064.5 | 90,323,254.9 ± 25,652,265.6 | |
| Median and [25%; 75%] | 95,629,716.0 [85,648,106.0; 114,371,13 | 90,972,342.0 [76,327,897.5; 108,277,588.0] | |
| **ST 27:1;O OA3__[M+H-H2O]__10.65** | | | 0.166 |
| Mean ± Standard deviation | 8,318,982.0 ± 13,729,367.7 | 7,988,797.2 ± 9,183,698.9 | |
| Median and [25%; 75%] | 3,075,249.0 [1,198,700.3; 7,697,709.8] | 4,823,692.6 [3,174,148.4; 8,630,186.4] | |
| **TG 40:0\|TG 10:0_14:0_16:0__[M+NH4]__9.27** | | | <0.001 |
| Mean ± Standard deviation | 47,601.1 ± 214,018.8 | 2,110,813.5 ± 5,714,322.0 | |
| Median and [25%; 75%] | 886.5 [394.5; 1,473.5] | 55,376.3 [3,017.3; 1,023,338.1] | |
| **TG 40:0\|TG 12:0_12:0_16:0__[M+NH4]__9.25** | | | <0.001 |
| Mean ± Standard deviation | 47,319.3 ± 208,135.7 | 2,113,103.7 ± 5,718,055.1 | |
| Median and [25%; 75%] | 1,610.0 [1,281.9; 2,395.5] | 56,130.3 [3,743.3; 1,025,392.3] | |
| **TG 40:0\|TG 12:0_12:0_16:0__[M+Na]__9.25** | | | <0.001 |
| Mean ± Standard deviation | 14,207.3 ± 58,860.0 | 350,453.2 ± 812,264.4 | |
| Median and [25%; 75%] | 1,169.0 [931.8; 2,106.5] | 10,329.5 [2,472.1; 270,165.6] | |
| **TG 40:1\|TG 10:0_12:0_18:1__[M+NH4]__8.66** | | | <0.001 |
| Mean ± Standard deviation | 3,435.6 ± 11,643.6 | 443,359.7 ± 826,152.9 | |
| Median and [25%; 75%] | 166.0 [62.3; 468.5] | 18,033.1 [1,760.4; 377,607.1] | |
| **TG 42:0\|TG 12:0_14:0_16:0__[M+NH4]__9.99** | | | <0.001 |
| Mean ± Standard deviation | 156,751.5 ± 699,439.0 | 3,319,925.2 ± 7,031,062.7 | |
| Median and [25%; 75%] | 6,198.0 [4,359.0; 8,087.5] | 261,276.3 [26,511.9; 2,897,545.5] | |
| **TG 42:0\|TG 14:0_14:0_14:0__[M+Na]__10.0** | | | <0.001 |
| Mean ± Standard deviation | 22,677.3 ± 107,593.3 | 333,446.9 ± 612,098.1 | |
| Median and [25%; 75%] | 464.0 [304.0; 891.8] | 12,493.8 [1,480.9; 549,564.8] | |
| **TG 42:1\|TG 10:0_14:0_18:1__[M+NH4]__9.39** | | | <0.001 |

| | | | |
|---|---|---|---|
| Mean ± Standard deviation | 67,453.9 ± 296,708.8 | 1,942,710.1 ± 3,750,306.4 | |
| Median and [25%; 75%] | 2,781.0 [1,203.0; 3,977.3] | 255,936.3 [28,963.9; 1,608,085.9] | |
| **TG 42:1\|TG 12:0_12:0_18:1__[M+Na]__9.39** | | | <0.001 |
| Mean ± Standard deviation | 17,695.5 ± 76,997.2 | 359,386.6 ± 641,245.1 | |
| Median and [25%; 75%] | 1,065.0 [401.0; 1,431.5] | 42,912.3 [3,129.4; 423,319.6] | |
| **TG 42:2\|TG 10:0_14:0_18:2__[M+NH4]__8.74** | | | <0.001 |
| Mean ± Standard deviation | 14,969.1 ± 53,029.7 | 621,042.3 ± 1,152,393.5 | |
| Median and [25%; 75%] | 332.5 [108.1; 711.5] | 64,719.4 [9,308.4; 582,012.5] | |
| **TG 44:2\|TG 10:0_16:0_18:2__[M+NH4]__9.48** | | | <0.001 |
| Mean ± Standard deviation | 5,161.5 ± 10,694.9 | 2,349,599.7 ± 3,782,790.8 | |
| Median and [25%; 75%] | 1,221.5 [272.8; 4,749.0] | 441,881.4 [137,658.4; 2,908,338.8] | |
| **TG 44:2\|TG 10:0_16:0_18:2__[M+Na]__9.48** | | | <0.001 |
| Mean ± Standard deviation | 1,755.1 ± 6,880.4 | 470,253.8 ± 685,285.1 | |
| Median and [25%; 75%] | 352.0 [96.4; 1,181.6] | 116,456.0 [18,404.0; 817,690.4] | |
| **TG 46:2\|TG 12:0_16:0_18:2__[M+NH4]__10.21** | | | <0.001 |
| Mean ± Standard deviation | 636,464.0 ± 1,951,756.0 | 11,259,240.0 ± 13,009,611.5 | |
| Median and [25%; 75%] | 113,588.0 [24,151.3; 443,400.5] | 7,376,581.6 [3,681,563.3; 11,073,923.0] | |
| **TG 46:3\|TG 10:0_18:1_18:2__[M+NH4]__9.59** | | | <0.001 |
| Mean ± Standard deviation | 83,203.9 ± 334,290.6 | 1,911,847.6 ± 2,631,227.5 | |
| Median and [25%; 75%] | 5,653.5 [937.0; 15,341.8] | 750,397.5 [426,484.1; 2,301,025.0] | |
| **TG 48:0\|TG 16:0_16:0_16:0__[M+NH4]__11.56** | | | <0.001 |
| Mean ± Standard deviation | 602,384.6 ± 740,166.0 | 4,700,214.0 ± 2,816,941.1 | |
| Median and [25%; 75%] | 402,585.0 [162,245.8; 674,660.8] | 4,353,638.5 [2,880,972.6; 6,319,378.3] | |
| **TG 48:1\|TG 14:0_16:0_18:1__[M+NH4]__10.95** | | | <0.001 |
| Mean ± Standard deviation | 419,891.2 ± 544,540.7 | 4,225,207.1 ± 3,100,244.1 | |
| Median and [25%; 75%] | 256,664.0 [87,586.5; 489,064.3] | 3,591,902.5 [2,225,167.7; 4,966,081.6] | |
| **TG 48:2\|TG 12:0_18:1_18:1__[M+NH4]__10.31** | | | <0.001 |
| Mean ± Standard deviation | 100,357.5 ± 192,576.1 | 1,315,667.3 ± 1,244,797.2 | |
| Median and [25%; 75%] | 49,715.5 [12,450.5; 106,674.0] | 1,039,848.5 [625,142.3; 1,352,213.3] | |
| **TG 48:2\|TG 14:0_16:0_18:2__[M+NH4]__10.94** | | | <0.001 |
| Mean ± Standard deviation | 9,137,310.4 ± 9,354,442.3 | 63,451,657.0 ± 38,463,027.2 | |
| Median and [25%; 75%] | 6,659,605.0 [2,889,618.0; 10,943,868.5] | 60,207,725.0 [39,478,040.8; 73,837,799.0] | |
| **TG 48:2\|TG 16:0_16:1_16:1__[M+Na]__10.94** | | | <0.001 |
| Mean ± Standard deviation | 202,247.4 ± 217,779.2 | 991,045.1 ± 642,196.4 | |
| Median and [25%; 75%] | 109,252.5 [71,033.3; 235,833.0] | 725,240.8 [548,684.6; 1,423,504.1] | |
| **TG 48:3\|TG 12:0_18:1_18:2__[M+NH4]__10.3** | | | <0.001 |

| | | | |
|---|---|---|---|
| Mean ± Standard deviation | 2,192,765.6 ± 3,266,143.1 | 19,517,042.3 ± 15,753,089.0 | |
| Median and [25%; 75%] | 1,202,668.5 [502,684.4; 2,719,719.3] | 15,164,249.8 [11,054,203.7; 23,065,251.8] | |
| **TG 48:3\|TG 16:1_16:1_16:1__[M+Na]__10.29** | | | <0.001 |
| Mean ± Standard deviation | 194,148.3 ± 269,567.6 | 1,249,793.4 ± 755,884.6 | |
| Median and [25%; 75%] | 92,776.0 [30,167.0; 255,851.0] | 1,274,181.5 [759,977.7; 1,505,193.6] | |
| **TG 48:4\|TG 12:0_18:2_18:2__[M+NH4]__9.67** | | | <0.001 |
| Mean ± Standard deviation | 197,705.6 ± 628,320.1 | 2,507,098.6 ± 3,427,578.2 | |
| Median and [25%; 75%] | 52,363.0 [8,672.5; 119,310.8] | 1,229,213.0 [877,313.6; 1,885,097.1] | |
| **TG 48:4\|TG 12:0_18:2_18:2__[M+Na]__9.67** | | | <0.001 |
| Mean ± Standard deviation | 43,557.8 ± 153,133.9 | 488,307.4 ± 582,043.7 | |
| Median and [25%; 75%] | 6,612.5 [2,124.0; 20,964.6] | 295,266.0 [200,158.1; 419,646.6] | |
| **TG 49:2\|TG 15:0_16:0_18:2__[M+NH4]__11.27** | | | <0.001 |
| Mean ± Standard deviation | 285,841.1 ± 314,036.7 | 3,292,605.8 ± 2,656,947.1 | |
| Median and [25%; 75%] | 155,409.0 [42,035.4; 456,427.8] | 2,773,459.8 [1,498,373.5; 4,200,139.9] | |
| Mean ± Standard deviation | | | <0.001 |
| Mean ± Standard deviation | 80,012.9 ± 107,647.8 | 974,850.4 ± 838,843.2 | |
| Median and [25%; 75%] | 40,978.0 [11,442.4; 101,519.8] | 720,563.5 [424,439.5; 1,216,000.6] | |
| **TG 50:0\|TG 16:0_16:0_18:0__[M+NH4]__12.6** | | | 0.075 |
| Mean ± Standard deviation | 2,668,716.7 ± 2,652,973.9 | 6,966,045.5 ± 6,727,723.7 | |
| Median and [25%; 75%] | 1,747,219.0 [1,122,597.8; 2,812,301.3] | 6,406,088.0 [1,071,474.9; 9,764,162.6] | |
| **TG 50:1\|TG 16:0_16:0_18:1__[M+NH4]__11.66** | | | <0.001 |
| Mean ± Standard deviation | 8,063,979.0 ± 5,218,346.6 | 26,652,084.3 ± 12,479,401.3 | |
| Median and [25%; 75%] | 6,673,906.5 [3,880,691.5; 11,990,288.0] | 22,971,780.5 [18,688,192.2; 36,686,367.5] | |
| **TG 50:2;O3\|TG 16:0_18:2_16:0;O3__[M+NH4]__10.98** | | | 0.010 |
| Mean ± Standard deviation | 13,150,555.6 ± 6,818,423.5 | 51,977,750.0 ± 108,822,140.0 | |
| Median and [25%; 75%] | 12,536,113.0 [7,413,747.0; 17,228,955.5 | 23,434,093.8 [12,546,171.9; 42,872,115.5] | |
| **TG 50:2\|TG 16:0_16:0_18:2__[M+NH4]__11.63** | | | <0.001 |
| Mean ± Standard deviation | 104,622,081.1 ± 58,486,713.4 | 271,581,554.3 ± 79,977,139.6 | |
| Median and [25%; 75%] | 99,768,740.0 [53,270,043.0; 146,277,69 | 276,440,726.0 [239,516,394.0; 334,922,528.0] | |
| **TG 50:2\|TG 16:0_16:0_18:2__[M+Na]__11.63** | | | 0.002 |
| Mean ± Standard deviation | 1,400,615.6 ± 601,980.7 | 2,068,854.3 ± 763,327.7 | |
| Median and [25%; 75%] | 1,275,177.5 [886,497.3; 1,820,555.8] | 2,087,132.3 [1,336,829.6; 2,691,861.9] | |
| **TG 50:2\|TG 16:0_16:1_18:1__[M+NH4]__11.66** | | | <0.001 |
| Mean ± Standard deviation | 105,015,733.7 ± 58,138,675.3 | 271,581,554.3 ± 79,977,139.6 | |
| Median and [25%; 75%] | 99,768,740.0 [54,794,914.0; 146,277,69 | 276,440,726.0 [239,516,394.0; 334,922,528.0] | |
| **TG 50:3\|TG 16:0_16:1_18:2__[M+NH4]__11.04** | | | <0.001 |

| | | | |
|---|---|---|---|
| Mean ± Standard deviation | 50,151,913.1 ± 32,784,701.6 | 168,537,749.9 ± 84,487,261.7 | |
| Median and [25%; 75%] | 40,419,409.0 [23,255,581.0; 64,944,815 | 178,018,856.0 [100,680,353.0; 233,129,142.0] | |
| **TG 50:3\|TG 16:0_16:1_18:2__[M+Na]__11.04** | | | <0.001 |
| Mean ± Standard deviation | 1,101,668.4 ± 342,482.2 | 1,821,644.6 ± 607,465.5 | |
| Median and [25%; 75%] | 1,129,501.5 [799,853.5; 1,359,560.0] | 1,935,285.0 [1,353,396.4; 2,279,013.1] | |
| **TG 50:4\|TG 14:0_16:0_20:4__[M+NH4]__10.74** | | | <0.001 |
| Mean ± Standard deviation | 10,895,642.8 ± 9,002,470.3 | 43,120,084.4 ± 21,464,564.9 | |
| Median and [25%; 75%] | 10,283,163.0 [4,180,598.5; 13,001,313.5 | 43,504,275.0 [22,880,626.0; 61,362,113.5] | |
| **TG 50:4\|TG 14:0_18:2_18:2__[M+NH4]__10.4** | | | <0.001 |
| Mean ± Standard deviation | 10,836,094.2 ± 8,943,467.3 | 43,136,844.0 ± 21,520,309.2 | |
| Median and [25%; 75%] | 10,202,310.0 [4,109,710.4; 13,002,587.9 | 43,527,262.5 [22,885,139.8; 61,145,743.3] | |
| **TG 50:4\|TG 14:0_18:2_18:2__[M+Na]__10.4** | | | <0.001 |
| Mean ± Standard deviation | 537,633.0 ± 458,854.8 | 1,758,972.1 ± 606,343.3 | |
| Median and [25%; 75%] | 402,129.5 [223,895.3; 684,037.0] | 1,738,304.0 [1,507,971.0; 2,246,269.6] | |
| **TG 50:4\|TG 16:0_16:1_18:3__[M+NH4]__10.58** | | | <0.001 |
| Mean ± Standard deviation | 10,734,477.7 ± 8,858,051.4 | 42,997,806.0 ± 21,358,726.9 | |
| Median and [25%; 75%] | 9,760,825.5 [4,183,686.3; 12,975,473.1] | 43,560,652.5 [22,909,627.5; 58,709,091.5] | |
| **TG 50:4\|TG 16:1_16:1_18:2__[M+NH4]__10.25** | | | <0.001 |
| Mean ± Standard deviation | 10,904,136.7 ± 9,005,946.9 | 43,228,175.9 ± 21,551,047.7 | |
| Median and [25%; 75%] | 10,283,375.5 [4,182,773.8; 13,006,725.5 | 43,547,051.0 [22,903,207.3; 61,419,589.0] | |
| **TG 50:5\|TG 14:0_18:2_18:3__[M+NH4]__9.79** | | | <0.001 |
| Mean ± Standard deviation | 180,886.4 ± 255,846.1 | 1,388,581.0 ± 927,431.7 | |
| Median and [25%; 75%] | 126,088.5 [22,504.3; 204,729.0] | 1,018,093.8 [742,542.3; 1,766,277.0] | |
| **TG 50:5\|TG 14:1_18:2_18:2__[M+NH4]__9.95** | | | <0.001 |
| Mean ± Standard deviation | 187,766.5 ± 232,707.2 | 1,475,847.0 ± 1,167,951.2 | |
| Median and [25%; 75%] | 127,149.0 [46,217.9; 195,455.5] | 947,608.0 [566,517.4; 2,270,248.8] | |
| **TG 50:5\|TG 16:0_18:2_16:3__[M+NH4]__10.15** | | | <0.001 |
| Mean ± Standard deviation | 99,318.5 ± 185,265.8 | 797,516.9 ± 962,423.4 | |
| Median and [25%; 75%] | 43,886.5 [14,884.3; 80,865.3] | 487,875.8 [297,381.4; 900,540.1] | |
| **TG 51:0\|TG 17:0_17:0_17:0__[M+Na]__10.53** | | | <0.001 |
| Mean ± Standard deviation | 10,602,979.6 ± 7,556,989.5 | 35,932,240.9 ± 40,517,603.3 | |
| Median and [25%; 75%] | 9,604,514.5 [5,009,672.3; 14,159,581.8] | 24,641,843.3 [15,374,781.5; 37,380,385.8] | |
| **TG 51:1\|TG 16:0_17:0_18:1__[M+NH4]__12.49** | | | <0.001 |
| Mean ± Standard deviation | 2,010,060.4 ± 1,042,466.9 | 7,930,386.4 ± 4,143,102.3 | |
| Median and [25%; 75%] | 2,001,133.8 [1,126,569.9; 2,492,371.3] | 7,918,516.1 [4,659,777.3; 10,723,364.5] | |
| **TG 51:3;O\|TG 18:1_18:1_15:1;O__[M+NH4]__11.76** | | | <0.001 |

| | | | |
|---|---|---|---|
| Mean ± Standard deviation | 17,448,433.4 ± 9,445,512.2 | 29,821,247.5 ± 10,721,031.1 | |
| Median and [25%; 75%] | 14,851,895.0 [10,449,118.0; 23,313,742 | 32,054,568.3 [23,075,374.0; 38,199,739.3] | |
| **TG 51:3\|TG 15:0_18:1_18:2__[M+NH4]__11.4** | | | <0.001 |
| Mean ± Standard deviation | 3,654,737.9 ± 3,560,172.3 | 14,838,237.6 ± 8,913,024.4 | |
| Median and [25%; 75%] | 2,652,515.5 [556,766.0; 4,920,208.5] | 13,179,344.5 [7,819,827.8; 19,760,559.1] | |
| **TG 51:4\|TG 15:0_18:2_18:2__[M+NH4]__10.75** | | | <0.001 |
| Mean ± Standard deviation | 1,084,120.9 ± 1,197,779.1 | 3,246,155.9 ± 2,633,804.8 | |
| Median and [25%; 75%] | 758,488.5 [117,680.6; 1,707,077.5] | 2,194,654.4 [1,432,006.8; 3,656,787.5] | |
| **TG 51:5;O\|TG 16:1_18:2_17:2;O__[M+NH4]__10.52** | | | <0.001 |
| Mean ± Standard deviation | 793,138.2 ± 619,176.1 | 2,714,915.7 ± 2,283,116.1 | |
| Median and [25%; 75%] | 669,049.5 [312,726.5; 1,082,370.1] | 2,251,638.0 [1,928,289.9; 2,839,812.8] | |
| **TG 51:5\|TG 15:1_18:2_18:2__[M+NH4]__10.33** | | | 0.005 |
| Mean ± Standard deviation | 69,664.5 ± 151,139.7 | 281,500.7 ± 654,708.3 | |
| Median and [25%; 75%] | 29,192.0 [7,749.4; 77,747.3] | 82,140.1 [50,325.6; 172,906.5] | |
| **TG 52:1\|TG 16:0_18:0_18:1__[M+NH4]__12.63** | | | <0.001 |
| Mean ± Standard deviation | 20,535,367.6 ± 14,019,551.1 | 57,601,926.8 ± 16,480,149.1 | |
| Median and [25%; 75%] | 17,597,778.3 [8,475,424.3; 25,085,939.8 | 59,995,019.0 [44,686,789.5; 70,654,839.5] | |
| **TG 52:1\|TG 17:0_17:0_18:1__[M+Na]__12.62** | | | 0.002 |
| Mean ± Standard deviation | 677,288.4 ± 313,702.5 | 1,002,508.4 ± 324,233.0 | |
| Median and [25%; 75%] | 650,492.0 [485,191.0; 811,220.3] | 981,939.5 [800,537.4; 1,187,308.9] | |
| **TG 52:2\|TG 16:0_18:1_18:1__[M+NH4]__11.76** | | | <0.001 |
| Mean ± Standard deviation | 57,697,961.1 ± 34,680,863.3 | 107,437,307.7 ± 49,268,773.2 | |
| Median and [25%; 75%] | 51,685,812.0 [28,768,385.3; 78,684,420 | 107,951,953.0 [79,669,645.3; 134,807,551.0] | |
| **TG 52:3;O2\|TG 16:0_18:2_18:1;O2__[M+NH4]__7.97** | | | <0.001 |
| Mean ± Standard deviation | 3,597.4 ± 7,524.9 | 353,191.7 ± 1,386,335.4 | |
| Median and [25%; 75%] | 1,572.0 [244.3; 3,225.6] | 6,021.8 [3,723.9; 8,511.4] | |
| **TG 52:3;O\|TG 16:0_18:1_18:2;O OA1__[M+NH4]__9.61** | | | <0.001 |
| Mean ± Standard deviation | 4,269.2 ± 7,129.5 | 259,169.6 ± 712,012.2 | |
| Median and [25%; 75%] | 2,607.3 [907.3; 4,757.3] | 65,350.8 [22,369.6; 156,848.1] | |
| **TG 52:3;O\|TG 16:0_18:2_18:1;O__[M+NH4]__8.98** | | | <0.001 |
| Mean ± Standard deviation | 4,727.6 ± 13,229.4 | 543,838.2 ± 2,142,038.4 | |
| Median and [25%; 75%] | 332.5 [163.8; 733.0] | 4,019.0 [1,735.9; 14,024.8] | |
| **TG 52:3\|TG 16:0_18:1_18:2__[M+NH4]__11.81** | | | <0.001 |
| Mean ± Standard deviation | 379,748,975.4 ± 138,864,551.5 | 570,476,725.0 ± 141,319,285.4 | |
| Median and [25%; 75%] | 352,559,632.0 [283,082,184.0; 482,591, | 598,648,416.0 [488,812,536.0; 665,778,108.0] | |
| **TG 52:4;O\|TG 16:0_18:1_18:3;O__[M+NH4]__9.19** | | | <0.001 |

| | | | |
|---|---|---|---|
| Mean ± Standard deviation | 5,627.3 ± 8,406.7 | 862,422.9 ± 3,027,087.5 | |
| Median and [25%; 75%] | 2,901.5 [1,285.0; 6,151.3] | 117,928.8 [12,152.4; 178,736.6] | |
| **TG 52:4;O\|TG 16:0_18:2_18:2;O__[M+NH4]__9.0** | | | <0.001 |
| Mean ± Standard deviation | 6,007.2 ± 8,840.8 | 860,864.2 ± 3,019,154.9 | |
| Median and [25%; 75%] | 2,993.3 [1,297.8; 6,573.4] | 117,959.5 [12,176.1; 182,339.5] | |
| **TG 52:4;O\|TG 18:2_18:2_16:0;O__[M+NH4]__8.61** | | | <0.001 |
| Mean ± Standard deviation | 1,844.2 ± 7,317.0 | 1,030,114.1 ± 4,100,220.1 | |
| Median and [25%; 75%] | 122.3 [38.1; 336.5] | 2,016.0 [903.5; 6,555.0] | |
| **TG 52:4\|TG 16:0_16:0_20:4__[M+NH4]__11.49** | | | <0.001 |
| Mean ± Standard deviation | 292,713,270.2 ± 151,777,976.7 | 450,644,436.3 ± 169,971,782.6 | |
| Median and [25%; 75%] | 301,848,144.0 [171,073,560.0; 422,347, | 478,282,052.0 [397,461,544.0; 574,022,534.0] | |
| **TG 52:4\|TG 16:0_18:2_18:2__[M+NH4]__11.04** | | | 0.001 |
| Mean ± Standard deviation | 287,887,712.0 ± 154,294,226.5 | 439,176,095.8 ± 170,177,274.3 | |
| Median and [25%; 75%] | 297,898,464.0 [144,981,873.5; 419,208, | 469,748,544.0 [360,833,500.0; 573,245,838.0] | |
| **TG 52:4\|TG 16:0_18:2_18:2__[M+Na]__11.04** | | | 0.455 |
| Mean ± Standard deviation | 4,303,255.2 ± 1,171,396.5 | 4,589,640.2 ± 1,330,263.8 | |
| Median and [25%; 75%] | 3,957,645.0 [3,460,925.3; 5,015,977.0] | 4,709,954.5 [3,417,601.3; 5,688,397.4] | |
| **TG 52:4\|TG 16:1_18:1_18:2__[M+NH4]__10.52** | | | <0.001 |
| Mean ± Standard deviation | 2,169,387.4 ± 1,542,327.8 | 6,903,791.8 ± 7,588,125.9 | |
| Median and [25%; 75%] | 1,765,396.0 [962,797.3; 3,012,827.6] | 4,755,021.5 [2,803,593.6; 7,670,846.3] | |
| **TG 52:5\|TG 16:0_16:1_20:4__[M+NH4]__10.88** | | | <0.001 |
| Mean ± Standard deviation | 32,224,758.1 ± 20,251,943.0 | 88,271,504.2 ± 69,433,551.6 | |
| Median and [25%; 75%] | 29,787,649.0 [16,794,656.5; 41,744,048 | 72,780,000.0 [55,064,416.0; 109,746,787.0] | |
| **TG 52:5\|TG 16:0_18:1_18:4__[M+Na]__10.71** | | | <0.001 |
| Mean ± Standard deviation | 1,651,156.0 ± 690,116.4 | 2,188,159.3 ± 437,136.9 | |
| Median and [25%; 75%] | 1,547,675.5 [1,279,698.9; 2,027,577.3] | 2,194,851.3 [1,866,561.5; 2,504,496.8] | |
| **TG 52:5\|TG 16:0_18:2_18:3__[M+NH4]__10.7** | | | 0.014 |
| Mean ± Standard deviation | 20,092,993.6 ± 17,777,240.7 | 49,245,736.0 ± 70,973,884.4 | |
| Median and [25%; 75%] | 11,826,964.5 [6,606,855.6; 32,008,879.0 | 25,007,414.5 [15,071,221.7; 43,908,448.0] | |
| **TG 52:5\|TG 16:1_18:2_18:2__[M+NH4]__10.52** | | | <0.001 |
| Mean ± Standard deviation | 24,833,327.1 ± 16,758,700.1 | 70,550,136.5 ± 71,210,543.0 | |
| Median and [25%; 75%] | 23,352,363.0 [10,127,764.4; 37,620,905 | 48,370,638.0 [33,264,852.8; 70,678,864.5] | |
| **TG 52:5\|TG 16:1_18:2_18:2__[M+Na]__10.52** | | | 0.001 |
| Mean ± Standard deviation | 1,605,634.5 ± 655,140.5 | 2,146,885.5 ± 461,765.3 | |
| Median and [25%; 75%] | 1,569,508.5 [1,342,561.8; 2,074,370.3] | 2,239,427.3 [1,735,612.6; 2,501,633.9] | |
| **TG 52:6\|TG 16:1_16:1_20:4__[M+NH4]__10.25** | | | <0.001 |

| | | | |
|---|---|---|---|
| Mean ± Standard deviation | 952,703.7 ± 1,369,622.9 | 4,574,605.3 ± 6,112,530.7 | |
| Median and [25%; 75%] | 631,326.5 [298,729.9; 961,607.0] | 2,234,880.3 [1,515,006.6; 3,628,273.6] | |
| **TG 52:6\|TG 16:1_18:2_18:3 OA1__[M+NH4]__9.94** | | | 0.001 |
| Mean ± Standard deviation | 965,605.7 ± 1,082,679.2 | 4,623,287.6 ± 6,185,194.5 | |
| Median and [25%; 75%] | 637,585.5 [208,753.8; 1,199,695.3] | 2,376,195.3 [832,326.6; 4,737,778.6] | |
| **TG 52:6\|TG 16:1_18:2_18:3__[M+NH4]__10.11** | | | 0.002 |
| Mean ± Standard deviation | 1,090,327.3 ± 1,459,221.0 | 4,445,013.8 ± 6,199,989.6 | |
| Median and [25%; 75%] | 662,566.0 [267,069.8; 1,254,079.4] | 2,208,151.8 [904,147.1; 3,737,039.8] | |
| **TG 53:2;O2\|TG 16:0_18:1_19:1;O2__[M+NH4]__11.25** | | | 0.260 |
| Mean ± Standard deviation | 7,313,450.5 ± 8,954,234.5 | 8,884,204.4 ± 7,066,261.8 | |
| Median and [25%; 75%] | 4,280,727.5 [2,309,915.5; 8,602,669.5] | 8,519,609.5 [3,374,617.0; 13,016,363.6] | |
| **TG 53:2;O2\|TG 16:0_18:2_19:0;O2__[M+NH4]__10.92** | | | 0.606 |
| Mean ± Standard deviation | 4,915,774.9 ± 5,275,571.5 | 5,792,353.7 ± 5,151,661.4 | |
| Median and [25%; 75%] | 3,014,978.5 [1,832,532.5; 5,917,396.8] | 4,427,265.4 [888,506.0; 8,539,943.9] | |
| **TG 53:2\|TG 17:0_18:1_18:1__[M+NH4]__12.53** | | | <0.001 |
| Mean ± Standard deviation | 5,460,753.3 ± 2,599,758.7 | 13,757,084.5 ± 5,007,321.4 | |
| Median and [25%; 75%] | 5,552,870.0 [3,759,244.8; 7,043,978.0] | 13,378,921.4 [10,778,266.3; 17,999,478.0] | |
| **TG 53:2\|TG 17:0_18:1_18:1__[M+Na]__12.53** | | | <0.001 |
| Mean ± Standard deviation | 375,746.8 ± 240,918.2 | 624,228.6 ± 240,654.3 | |
| Median and [25%; 75%] | 374,768.3 [164,343.3; 523,992.1] | 689,346.8 [543,483.9; 781,090.6] | |
| **TG 53:3;O2\|TG 16:0_18:2_19:1;O2__[M+NH4]__10.65** | | | 0.365 |
| Mean ± Standard deviation | 14,924,399.5 ± 16,562,165.3 | 16,498,451.5 ± 12,567,641.8 | |
| Median and [25%; 75%] | 8,615,775.0 [5,040,806.8; 17,974,544.6] | 14,951,992.8 [8,003,321.4; 24,475,328.5] | |
| **TG 53:3\|TG 17:0_18:1_18:2__[M+NH4]__12.11** | | | <0.001 |
| Mean ± Standard deviation | 6,790,275.0 ± 4,932,455.5 | 18,711,989.9 ± 10,077,071.1 | |
| Median and [25%; 75%] | 6,469,220.8 [2,335,852.8; 9,773,615.3] | 17,783,424.5 [12,350,926.8; 22,784,404.3] | |
| **TG 53:4\|TG 17:1_18:1_18:2__[M+NH4]__11.5** | | | <0.001 |
| Mean ± Standard deviation | 3,167,920.2 ± 2,374,703.1 | 7,346,134.7 ± 4,171,942.0 | |
| Median and [25%; 75%] | 2,898,263.5 [1,401,293.4; 4,164,538.3] | 6,294,728.3 [4,405,961.4; 10,113,568.6] | |
| **TG 53:5\|TG 17:1_18:2_18:2__[M+NH4]__10.91** | | | <0.001 |
| Mean ± Standard deviation | 139,547.8 ± 163,008.0 | 886,636.8 ± 1,814,024.1 | |
| Median and [25%; 75%] | 78,788.0 [26,697.0; 203,654.3] | 337,345.9 [212,191.5; 647,558.6] | |
| **TG 53:6;O\|TG 18:2_18:2_17:2;O__[M+NH4]__10.62** | | | 0.066 |
| Mean ± Standard deviation | 1,365,351.2 ± 1,463,099.2 | 6,046,211.5 ± 13,105,320.9 | |
| Median and [25%; 75%] | 1,251,166.8 [361,837.4; 1,797,806.0] | 1,479,063.3 [903,949.6; 4,090,744.4] | |
| **TG 54:1\|TG 18:0_18:0_18:1 OA1__[M+NH4]__12.81** | | | <0.001 |

| | | | |
|---|---|---|---|
| Mean ± Standard deviation | 2,632,743.6 ± 3,509,241.8 | 7,738,614.0 ± 4,028,666.0 | |
| Median and [25%; 75%] | 2,092,431.8 [1,138,968.4; 2,781,201.5] | 6,856,151.3 [4,887,805.2; 8,975,457.9] | |
| **TG 54:1\|TG 18:0_18:0_18:1__[M+NH4]__12.81** | | | <0.001 |
| Mean ± Standard deviation | 2,725,289.2 ± 1,637,178.1 | 5,704,722.0 ± 2,088,827.7 | |
| Median and [25%; 75%] | 2,607,383.5 [1,818,350.3; 3,191,748.8] | 5,633,564.6 [4,059,037.8; 7,451,934.8] | |
| **TG 54:2\|TG 18:0_18:1_18:1__[M+NH4]__12.66** | | | <0.001 |
| Mean ± Standard deviation | 26,085,693.3 ± 16,186,328.5 | 60,422,733.0 ± 21,043,845.9 | |
| Median and [25%; 75%] | 25,156,769.0 [15,486,529.3; 31,493,137 | 58,463,538.5 [42,804,322.8; 79,756,708.0] | |
| **TG 54:2\|TG 18:0_18:1_18:1__[M+Na]__12.66** | | | 0.248 |
| Mean ± Standard deviation | 772,113.5 ± 323,748.4 | 869,027.3 ± 264,162.0 | |
| Median and [25%; 75%] | 799,856.0 [501,886.5; 945,405.9] | 871,463.8 [759,065.6; 1,038,218.6] | |
| **TG 54:3\|TG 18:0_18:1_18:2 OA1__[M+NH4]__11.88** | | | <0.001 |
| Mean ± Standard deviation | 13,546,590.1 ± 9,501,022.8 | 34,308,379.8 ± 32,427,568.9 | |
| Median and [25%; 75%] | 10,427,878.0 [6,988,038.1; 17,079,294.3 | 23,681,981.0 [17,520,735.1; 39,202,550.5] | |
| **TG 54:3\|TG 18:0_18:1_18:2__[M+NH4]__12.41** | | | <0.001 |
| Mean ± Standard deviation | 88,553,338.9 ± 40,014,098.2 | 172,556,439.0 ± 59,742,922.1 | |
| Median and [25%; 75%] | 79,885,284.0 [62,720,728.5; 109,577,54 | 163,067,340.0 [136,626,014.0; 203,247,150.0] | |
| **TG 54:3\|TG 18:1_18:1_18:1__[M+Na]__12.43** | | | 0.003 |
| Mean ± Standard deviation | 1,076,564.7 ± 597,062.1 | 1,646,399.5 ± 695,589.2 | |
| Median and [25%; 75%] | 1,065,683.5 [612,739.6; 1,366,035.5] | 1,579,812.8 [1,263,435.7; 1,863,628.4] | |
| **TG 54:4\|TG 16:0_18:1_20:3__[M+NH4]__12.17** | | | <0.001 |
| Mean ± Standard deviation | 134,466,530.1 ± 69,980,702.5 | 260,157,207.8 ± 128,336,551.8 | |
| Median and [25%; 75%] | 127,212,406.0 [86,084,002.0; 183,808,7 | 242,396,912.0 [192,909,196.0; 316,844,036.0] | |
| **TG 54:4\|TG 18:1_18:1_18:2__[M+NH4]__11.86** | | | <0.001 |
| Mean ± Standard deviation | 130,603,463.5 ± 71,782,131.3 | 261,279,858.5 ± 132,885,173.3 | |
| Median and [25%; 75%] | 120,999,348.0 [83,407,435.0; 180,667,2 | 242,401,058.0 [193,486,039.0; 319,209,304.0] | |
| **TG 54:4\|TG 18:1_18:1_18:2__[M+Na]__11.86** | | | 0.298 |
| Mean ± Standard deviation | 2,130,258.7 ± 939,705.7 | 2,548,665.8 ± 1,277,261.2 | |
| Median and [25%; 75%] | 2,291,313.0 [1,229,559.5; 2,714,202.0] | 2,470,889.9 [1,656,924.8; 2,947,887.9] | |
| **TG 54:5;O3\|TG 18:1_20:4_16:0;O3__[M+NH4]__10.92** | | | 0.873 |
| Mean ± Standard deviation | 3,177,434.8 ± 3,206,135.4 | 2,766,020.9 ± 2,362,079.9 | |
| Median and [25%; 75%] | 2,070,537.0 [875,811.0; 4,058,836.3] | 2,432,761.0 [1,034,910.1; 4,673,274.8] | |
| **TG 54:5\|TG 16:0_18:1_20:4__[M+NH4]__11.6** | | | 0.041 |
| Mean ± Standard deviation | 26,734,101.8 ± 14,841,848.4 | 89,491,281.3 ± 202,264,824.0 | |
| Median and [25%; 75%] | 23,486,579.0 [17,718,747.8; 30,737,531 | 40,270,238.5 [25,120,346.0; 56,597,392.0] | |
| **TG 54:5\|TG 18:1_18:2_18:2__[M+NH4]__11.25** | | | <0.001 |

| | | | |
|---|---|---|---|
| Mean ± Standard deviation | 64,963,595.6 ± 56,986,179.0 | 182,502,703.0 ± 202,590,590.0 | |
| Median and [25%; 75%] | 44,273,513.0 [22,483,293.5; 87,602,888 | 112,809,392.0 [73,535,326.5; 207,316,254.0] | |
| **TG 54:5\|TG 18:1_18:2_18:2__[M+Na]__11.25** | | | 0.232 |
| Mean ± Standard deviation | 634,815.7 ± 425,629.2 | 1,294,936.0 ± 1,920,558.0 | |
| Median and [25%; 75%] | 534,986.8 [384,774.8; 823,943.0] | 673,052.3 [462,610.9; 921,443.5] | |
| **TG 54:6\|TG 16:0_18:2_20:4__[M+NH4]__10.85** | | | 0.024 |
| Mean ± Standard deviation | 25,444,586.4 ± 16,332,245.1 | 124,280,731.3 ± 261,989,963.4 | |
| Median and [25%; 75%] | 21,891,373.0 [12,283,444.6; 37,662,292 | 39,632,000.3 [22,551,933.0; 77,370,983.0] | |
| **TG 54:6\|TG 18:2_18:2_18:2__[M+NH4]__10.62** | | | 0.005 |
| Mean ± Standard deviation | 27,142,872.0 ± 26,649,450.3 | 135,286,315.2 ± 266,121,297.9 | |
| Median and [25%; 75%] | 16,514,663.0 [8,766,022.0; 40,975,994.( | 42,177,726.0 [26,312,433.9; 79,211,799.0] | |
| **TG 54:7;O2\|TG 18:2_18:3_18:2;O2__[M+NH4]__6.64** | | | 0.014 |
| Mean ± Standard deviation | 2,506.5 ± 10,404.1 | 134,580.8 ± 513,773.9 | |
| Median and [25%; 75%] | 18.0 [0.0; 60.0] | 146.3 [22.9; 516.0] | |
| **TG 54:7;O\|TG 18:2_18:2_18:3;O__[M+NH4]__7.96** | | | 0.007 |
| Mean ± Standard deviation | 2,458.0 ± 9,788.5 | 1,758,157.9 ± 6,969,907.2 | |
| Median and [25%; 75%] | 77.8 [30.0; 198.0] | 222.0 [82.8; 16,046.5] | |
| **TG 54:7;O\|TG 18:3_18:3_18:1;O__[M+NH4]__7.62** | | | 0.096 |
| Mean ± Standard deviation | 1,330.5 ± 7,455.3 | 669,078.0 ± 2,576,368.6 | |
| Median and [25%; 75%] | 61.0 [31.0; 112.0] | 96.0 [57.4; 1,878.0] | |
| **TG 54:7\|TG 18:2_18:2_18:3__[M+NH4]__10.17** | | | 0.339 |
| Mean ± Standard deviation | 7,404,546.8 ± 12,087,890.1 | 50,296,565.3 ± 145,836,347.7 | |
| Median and [25%; 75%] | 1,457,783.0 [698,080.5; 9,114,838.1] | 2,817,490.3 [766,150.6; 17,089,182.8] | |
| **TG 54:8\|TG 14:0_18:2_22:6__[M+NH4]__9.9** | | | <0.001 |
| Mean ± Standard deviation | 217,507.0 ± 324,761.1 | 958,226.7 ± 604,981.9 | |
| Median and [25%; 75%] | 52,652.3 [2,476.8; 279,696.3] | 906,713.0 [653,333.5; 1,158,371.6] | |
| **TG 55:2\|TG 18:0_18:1_19:1__[M+NH4]__12.76** | | | <0.001 |
| Mean ± Standard deviation | 113,660.7 ± 60,571.5 | 378,342.4 ± 168,178.8 | |
| Median and [25%; 75%] | 111,244.5 [68,200.9; 153,785.4] | 353,866.5 [272,989.9; 511,074.9] | |
| **TG 55:3\|TG 18:1_18:1_19:1__[M+NH4]__12.57** | | | <0.001 |
| Mean ± Standard deviation | 206,100.9 ± 109,920.3 | 475,632.4 ± 140,025.6 | |
| Median and [25%; 75%] | 199,622.5 [121,722.4; 283,483.3] | 459,935.0 [382,511.3; 577,861.0] | |
| **TG 55:4;O2\|TG 18:1_18:2_19:1;O2__[M+NH4]__10.73** | | | 0.537 |
| Mean ± Standard deviation | 3,359,430.3 ± 4,136,436.0 | 2,434,303.6 ± 2,418,446.7 | |
| Median and [25%; 75%] | 1,802,112.5 [748,419.8; 4,945,564.4] | 1,949,191.9 [701,033.5; 3,582,210.8] | |
| **TG 55:5;O2\|TG 18:2_18:2_19:1;O2__[M+NH4]__10.27** | | | 0.087 |

| | | | |
|---|---|---|---|
| Mean ± Standard deviation | 739,978.0 ± 890,433.2 | 578,189.6 ± 906,972.4 | |
| Median and [25%; 75%] | 396,975.5 [211,095.0; 755,523.0] | 199,540.6 [49,745.4; 643,174.4] | |
| **TG 56:2\|TG 20:0_18:1_18:1__[M+NH4]__12.83** | | | <0.001 |
| Mean ± Standard deviation | 997,177.5 ± 1,244,907.5 | 2,763,471.4 ± 2,061,221.3 | |
| Median and [25%; 75%] | 731,327.0 [343,037.8; 1,123,669.8] | 2,535,350.0 [1,570,092.9; 3,463,429.0] | |
| **TG 56:3\|TG 18:1_18:1_20:1__[M+NH4]__12.69** | | | <0.001 |
| Mean ± Standard deviation | 1,272,720.6 ± 677,102.3 | 2,889,868.0 ± 1,988,221.9 | |
| Median and [25%; 75%] | 1,198,383.0 [771,373.0; 1,716,876.0] | 2,184,950.0 [1,652,340.3; 3,346,815.3] | |
| **TG 56:3\|TG 20:0_18:1_18:2__[M+NH4]__12.7** | | | <0.001 |
| Mean ± Standard deviation | 1,261,963.7 ± 667,654.4 | 2,889,727.0 ± 1,988,219.3 | |
| Median and [25%; 75%] | 1,204,047.5 [771,373.0; 1,625,785.8] | 2,184,950.0 [1,652,340.3; 3,346,815.3] | |
| **TG 56:4\|TG 18:1_20:1_18:2__[M+NH4]__12.46** | | | 0.011 |
| Mean ± Standard deviation | 2,027,963.3 ± 1,140,789.6 | 3,844,168.3 ± 2,964,131.9 | |
| Median and [25%; 75%] | 1,874,486.0 [1,077,443.5; 2,751,958.3] | 3,154,658.0 [1,979,677.3; 4,530,169.4] | |
| **TG 56:5\|TG 16:0_18:1_22:4__[M+NH4]__12.02** | | | 0.008 |
| Mean ± Standard deviation | 4,943,970.7 ± 4,386,993.7 | 9,469,883.1 ± 5,773,493.4 | |
| Median and [25%; 75%] | 3,544,709.5 [987,596.9; 8,272,559.3] | 10,238,061.6 [5,645,889.4; 12,265,191.4] | |
| **TG 56:5\|TG 18:0_18:1_20:4__[M+NH4]__12.29** | | | 0.075 |
| Mean ± Standard deviation | 5,537,766.8 ± 3,792,784.5 | 3,808,438.0 ± 3,658,793.6 | |
| Median and [25%; 75%] | 4,285,719.0 [2,940,232.1; 8,502,774.8] | 2,831,719.8 [1,082,574.3; 5,170,916.4] | |
| **TG 56:6\|TG 16:0_18:1_22:5__[M+NH4]__11.51** | | | 0.246 |
| Mean ± Standard deviation | 15,032,544.9 ± 10,809,977.6 | 20,080,231.0 ± 13,523,720.7 | |
| Median and [25%; 75%] | 12,814,504.5 [7,300,117.6; 19,559,451.5 | 18,510,922.0 [10,249,000.0; 33,471,281.9] | |
| **TG 56:6\|TG 18:1_18:1_20:4__[M+NH4]__11.7** | | | 0.194 |
| Mean ± Standard deviation | 9,164,760.5 ± 5,283,913.5 | 6,997,164.9 ± 4,675,244.0 | |
| Median and [25%; 75%] | 8,040,809.5 [5,664,382.4; 11,330,730.5] | 6,752,443.4 [4,238,154.1; 8,935,721.8] | |
| **TG 56:7\|TG 16:0_18:1_22:6__[M+NH4]__11.25** | | | 0.084 |
| Mean ± Standard deviation | 9,252,090.7 ± 12,187,929.1 | 13,947,827.9 ± 10,992,697.1 | |
| Median and [25%; 75%] | 5,798,930.0 [2,696,194.3; 9,891,152.6] | 12,546,824.3 [4,389,708.8; 20,485,361.5] | |
| **TG 56:7\|TG 16:0_18:2_22:5__[M+NH4]__10.94** | | | 0.679 |
| Mean ± Standard deviation | 7,172,164.5 ± 4,631,592.2 | 8,664,835.4 ± 7,359,797.8 | |
| Median and [25%; 75%] | 5,867,862.5 [4,138,766.3; 10,042,375.8] | 6,645,319.8 [2,017,420.5; 13,576,497.8] | |
| **TG 56:8\|TG 16:0_18:2_22:6__[M+NH4]__10.64** | | | 0.267 |
| Mean ± Standard deviation | 18,219,125.7 ± 17,783,240.3 | 22,301,239.8 ± 16,713,012.9 | |
| Median and [25%; 75%] | 13,055,680.5 [5,719,488.8; 21,379,836.0 | 20,212,052.5 [11,339,740.1; 28,948,365.9] | |
| **TG 56:8\|TG 16:0_18:2_22:6__[M+Na]__10.64** | | | 0.161 |

| | | | |
|---|---|---|---|
| Mean ± Standard deviation | 568,980.4 ± 466,616.4 | 405,582.1 ± 394,541.3 | |
| Median and [25%; 75%] | 453,222.0 [237,186.8; 721,458.4] | 323,422.0 [96,772.2; 574,803.9] | |
| **TG 56:8\|TG 18:2_18:2_20:4__[M+NH4]__10.27** | | | 0.432 |
| Mean ± Standard deviation | 5,917,459.3 ± 3,883,223.8 | 4,873,638.7 ± 3,522,256.8 | |
| Median and [25%; 75%] | 4,911,318.5 [3,148,626.3; 7,942,472.0] | 4,704,921.6 [2,712,887.8; 6,365,470.6] | |
| **TG 56:9\|TG 16:1_18:2_22:6__[M+NH4]__10.03** | | | 0.704 |
| Mean ± Standard deviation | 1,224,215.7 ± 1,288,567.4 | 1,289,998.5 ± 992,564.4 | |
| Median and [25%; 75%] | 1,032,223.5 [396,269.9; 1,498,489.3] | 1,152,958.5 [578,098.0; 2,056,771.1] | |
| **TG 56:9\|TG 18:2_18:2_20:5__[M+NH4]__9.85** | | | 0.717 |
| Mean ± Standard deviation | 489,032.0 ± 558,350.8 | 538,615.3 ± 495,606.1 | |
| Median and [25%; 75%] | 329,968.5 [130,924.5; 603,297.0] | 396,269.0 [170,750.6; 796,654.6] | |
| **TG 57:6;O2\|TG 18:1_20:4_19:1;O2__[M+NH4]__10.55** | | | 0.059 |
| Mean ± Standard deviation | 134,634.9 ± 233,361.8 | 55,423.9 ± 82,200.7 | |
| Median and [25%; 75%] | 43,916.0 [17,055.3; 138,103.8] | 17,942.0 [10,839.9; 64,093.0] | |
| **TG 58:1\|TG 16:0_24:0_18:1__[M+NH4]__13.03** | | | 0.001 |
| Mean ± Standard deviation | 307,059.6 ± 698,130.2 | 366,751.5 ± 218,248.9 | |
| Median and [25%; 75%] | 162,269.0 [125,996.9; 242,834.3] | 290,967.8 [228,925.5; 463,515.3] | |
| **TG 58:2\|TG 22:0_18:1_18:1__[M+NH4]__12.95** | | | <0.001 |
| Mean ± Standard deviation | 352,948.3 ± 692,256.2 | 887,212.9 ± 1,092,822.6 | |
| Median and [25%; 75%] | 152,332.5 [88,297.3; 375,586.1] | 648,201.3 [414,479.8; 874,109.9] | |
| **TG 58:3\|TG 22:0_18:1_18:2__[M+NH4]__12.86** | | | <0.001 |
| Mean ± Standard deviation | 220,963.9 ± 262,461.6 | 872,957.1 ± 1,507,867.7 | |
| Median and [25%; 75%] | 137,977.5 [55,030.4; 231,540.3] | 301,004.0 [203,540.3; 732,936.5] | |
| **TG 58:4\|TG 22:0_18:2_18:2__[M+NH4]__12.74** | | | 0.030 |
| Mean ± Standard deviation | 139,224.6 ± 188,740.6 | 870,681.3 ± 2,192,411.8 | |
| Median and [25%; 75%] | 71,164.0 [30,794.1; 152,169.9] | 133,802.0 [80,905.6; 426,835.1] | |
| **TG 58:5\|TG 18:0_18:1_22:4__[M+NH4]__12.53** | | | 0.050 |
| Mean ± Standard deviation | 128,961.6 ± 76,542.3 | 317,818.4 ± 417,270.9 | |
| Median and [25%; 75%] | 115,918.8 [80,005.8; 180,434.3] | 254,280.1 [79,618.6; 340,386.9] | |
| **TG 58:6\|TG 18:1_18:1_22:4__[M+NH4]__12.18** | | | 0.141 |
| Mean ± Standard deviation | 250,069.1 ± 190,578.5 | 380,321.2 ± 279,541.8 | |
| Median and [25%; 75%] | 205,572.5 [132,543.3; 299,996.0] | 374,541.8 [177,246.0; 591,977.9] | |
| **TG 58:7\|TG 18:1_18:1_22:5 OA1__[M+NH4]__11.59** | | | 0.595 |
| Mean ± Standard deviation | 457,618.0 ± 428,592.0 | 356,412.3 ± 283,778.8 | |
| Median and [25%; 75%] | 334,642.0 [176,260.8; 600,239.8] | 381,184.3 [87,468.1; 564,546.6] | |
| **TG 58:7\|TG 18:1_18:1_22:5__[M+NH4]__11.97** | | | 0.606 |

| | | | |
|---|---|---|---|
| Mean ± Standard deviation | 511,161.1 ± 434,735.3 | 599,746.2 ± 493,943.8 | |
| Median and [25%; 75%] | 385,339.0 [220,618.4; 654,978.8] | 443,778.3 [184,293.0; 988,481.8] | |
| **TG 58:8\|TG 18:1_18:1_22:6__[M+NH4]__11.38** | | | 0.232 |
| Mean ± Standard deviation | 993,567.7 ± 1,496,138.7 | 1,508,655.9 ± 1,671,915.8 | |
| Median and [25%; 75%] | 263,784.8 [151,979.3; 928,712.0] | 891,157.0 [272,169.5; 2,500,454.6] | |
| **TG 58:9\|TG 18:1_18:2_22:6__[M+NH4]__10.74** | | | 0.873 |
| Mean ± Standard deviation | 3,522,416.2 ± 4,138,343.1 | 3,742,127.3 ± 3,693,521.6 | |
| Median and [25%; 75%] | 2,192,622.5 [1,030,765.4; 4,145,719.4] | 2,498,354.6 [1,359,370.9; 5,961,210.4] | |
| **TG 58:9\|TG 18:1_20:4_20:4__[M+NH4]__10.55** | | | 0.667 |
| Mean ± Standard deviation | 4,574,859.5 ± 4,939,444.4 | 3,898,425.3 ± 3,643,116.4 | |
| Median and [25%; 75%] | 3,265,457.0 [1,216,134.3; 5,982,235.0] | 3,532,659.5 [1,441,657.8; 6,019,797.7] | |
| **TG 60:12\|TG 16:0_22:6_22:6__[M+NH4]__10.14** | | | 0.374 |
| Mean ± Standard deviation | 360,742.4 ± 1,009,779.0 | 132,867.5 ± 190,689.2 | |
| Median and [25%; 75%] | 63,122.5 [9,757.3; 269,853.4] | 27,117.8 [4,289.4; 172,722.6] | |
| **TG 60:3\|TG 24:0_18:1_18:2__[M+NH4]__12.96** | | | <0.001 |
| Mean ± Standard deviation | 58,230.5 ± 65,615.0 | 158,134.7 ± 180,070.7 | |
| Median and [25%; 75%] | 34,038.5 [12,863.5; 82,766.8] | 119,003.0 [74,107.1; 147,457.8] | |
| **TG O-34:1\|TG O-14:1_10:0_10:0__[M+Na]__5.89** | | | 0.913 |
| Mean ± Standard deviation | 498,192.3 ± 904,797.6 | 1,020,132.0 ± 2,886,736.7 | |
| Median and [25%; 75%] | 68,657.0 [10,285.9; 521,794.3] | 59,253.3 [12,416.8; 210,558.6] | |
| **TG O-43:1\|TG O-19:1_12:0_12:0__[M+NH4]__9.35** | | | <0.001 |
| Mean ± Standard deviation | 8,548.5 ± 37,580.8 | 406,065.9 ± 880,700.4 | |
| Median and [25%; 75%] | 289.8 [80.5; 552.1] | 22,439.3 [2,961.4; 252,493.3] | |
| **TG O-50:2\|TG O-19:2_15:0_16:0__[M+NH4]__11.28** | | | <0.001 |
| Mean ± Standard deviation | 33,584.1 ± 28,589.6 | 392,580.6 ± 427,370.4 | |
| Median and [25%; 75%] | 26,529.5 [13,019.0; 46,434.6] | 255,635.3 [134,698.9; 366,608.3] | |
| **TG O-51:0\|TG O-19:0_16:0_16:0__[M+NH4]__12.59** | | | 0.119 |
| Mean ± Standard deviation | 768,301.7 ± 741,567.4 | 1,963,408.2 ± 1,813,946.2 | |
| Median and [25%; 75%] | 590,488.5 [346,224.3; 832,511.4] | 1,674,022.0 [341,598.1; 3,443,710.8] | |
| **TG O-51:1\|TG O-19:1_16:0_16:0__[M+NH4]__12.26** | | | <0.001 |
| Mean ± Standard deviation | 26,461,247.9 ± 19,603,844.0 | 82,406,104.8 ± 38,998,797.8 | |
| Median and [25%; 75%] | 24,278,224.0 [10,228,277.0; 37,368,818 | 83,563,842.0 [51,699,272.3; 109,180,751.0] | |
| **TG O-51:2\|TG O-17:1_16:0_18:1__[M+NH4]__11.65** | | | <0.001 |
| Mean ± Standard deviation | 42,126,072.7 ± 33,506,005.8 | 149,225,598.1 ± 80,711,941.8 | |
| Median and [25%; 75%] | 36,292,528.0 [14,874,917.3; 59,210,706 | 134,798,852.0 [99,479,061.0; 215,144,272.0] | |
| **TG O-51:4\|TG O-15:0_18:2_18:2__[M+NH4]__10.21** | | | <0.001 |

| | | | |
|---|---|---|---|
| Mean ± Standard deviation | 3,159,354.9 ± 3,538,363.2 | 14,683,834.1 ± 9,470,464.0 | |
| Median and [25%; 75%] | 2,469,556.0 [826,736.1; 3,248,265.0] | 12,482,353.9 [7,733,416.0; 20,190,252.4] | |
| **TG O-53:1\|TG O-19:1_16:0_18:0__[M+NH4]__12.63** | | | <0.001 |
| Mean ± Standard deviation | 10,235,301.1 ± 7,380,474.2 | 25,107,438.6 ± 9,550,435.7 | |
| Median and [25%; 75%] | 8,701,994.0 [5,533,780.0; 12,400,527.3] | 24,946,453.5 [20,129,973.0; 27,015,453.0] | |
| **TG O-53:3\|TG O-17:1_18:1_18:1__[M+NH4]__11.75** | | | 0.003 |
| Mean ± Standard deviation | 327,836,939.9 ± 199,693,221.2 | 552,488,671.5 ± 263,620,830.1 | |
| Median and [25%; 75%] | 296,585,896.0 [172,105,166.0; 459,895, | 514,180,968.0 [460,861,108.0; 697,452,416.0] | |
| **TG O-53:3\|TG O-17:1_18:1_18:1__[M+Na]__11.75** | | | 0.080 |
| Mean ± Standard deviation | 877,720.0 ± 478,789.3 | 630,350.4 ± 282,229.4 | |
| Median and [25%; 75%] | 717,287.0 [543,740.8; 1,081,101.0] | 538,033.8 [433,114.1; 797,261.0] | |
| **TG O-53:4\|TG O-17:0_18:2_18:2__[M+NH4]__11.12** | | | 0.008 |
| Mean ± Standard deviation | 179,202,407.3 ± 135,134,006.9 | 347,683,262.6 ± 233,177,881.6 | |
| Median and [25%; 75%] | 162,887,792.0 [69,589,211.0; 259,877,1 | 258,760,412.0 [195,189,934.0; 534,315,838.0] | |
| **TG O-53:5\|TG O-17:0_18:2_18:3__[M+NH4]__10.53** | | | <0.001 |
| Mean ± Standard deviation | 8,328,007.8 ± 7,899,507.5 | 34,170,718.0 ± 41,004,866.5 | |
| Median and [25%; 75%] | 7,201,537.0 [2,933,767.8; 11,985,159.8] | 22,603,069.9 [12,334,353.3; 35,791,854.3] | |
| **TG O-53:5\|TG O-19:3_16:0_18:2__[M+NH4]__10.53** | | | <0.001 |
| Mean ± Standard deviation | 8,323,866.6 ± 7,892,980.8 | 33,475,570.3 ± 41,251,900.0 | |
| Median and [25%; 75%] | 7,202,087.5 [2,612,604.3; 11,985,517.8] | 21,684,194.3 [12,334,353.3; 35,791,854.3] | |
| **TG O-54:2\|TG O-18:0_18:1_18:1__[M+NH4]__12.52** | | | <0.001 |
| Mean ± Standard deviation | 2,290,043.1 ± 1,443,520.2 | 5,393,476.3 ± 2,892,638.2 | |
| Median and [25%; 75%] | 2,056,417.5 [1,301,912.1; 3,030,866.5] | 5,437,356.6 [3,211,784.0; 6,507,616.2] | |
| **TG O-55:1\|TG O-19:1_18:0_18:0__[M+NH4]__12.81** | | | <0.001 |
| Mean ± Standard deviation | 1,353,300.2 ± 2,868,162.5 | 3,088,067.3 ± 1,798,578.3 | |
| Median and [25%; 75%] | 739,863.5 [527,298.5; 1,126,945.0] | 2,710,457.5 [1,816,945.5; 3,854,510.6] | |
| **TG O-55:4\|TG O-19:2_18:1_18:1__[M+NH4]__11.87** | | | 0.001 |
| Mean ± Standard deviation | 65,799,975.9 ± 58,838,672.9 | 175,483,674.4 ± 193,272,540.2 | |
| Median and [25%; 75%] | 50,121,814.0 [25,982,969.5; 91,912,742 | 103,534,268.0 [65,668,657.3; 184,338,451.0] | |
| **TG O-55:5\|TG O-19:1_16:0_20:4__[M+NH4]__11.58** | | | 0.504 |
| Mean ± Standard deviation | 11,177,892.6 ± 10,537,283.4 | 10,128,076.2 ± 9,344,633.0 | |
| Median and [25%; 75%] | 7,777,768.5 [4,993,681.8; 14,253,314.0] | 6,873,192.4 [3,223,772.4; 16,316,717.6] | |
| **TG O-55:6\|TG O-19:2_18:2_18:2__[M+NH4]__10.62** | | | 0.061 |
| Mean ± Standard deviation | 18,582,545.8 ± 29,754,007.6 | 129,584,755.7 ± 349,937,106.6 | |
| Median and [25%; 75%] | 13,596,438.0 [4,345,170.3; 20,116,516.5 | 20,979,394.9 [10,376,160.1; 68,923,795.0] | |
| **TG O-57:2\|TG O-19:1_18:0_20:1__[M+NH4]__12.83** | | | <0.001 |

| | | | |
|---|---|---|---|
| Mean ± Standard deviation | 342,672.4 ± 400,637.8 | 953,850.5 ± 923,867.1 | |
| Median and [25%; 75%] | 235,587.0 [142,538.0; 364,559.5] | 719,157.0 [415,482.0; 1,097,306.1] | |
| **VAE 18:0__[M+Na]__4.55** | | | <0.001 |
| Mean ± Standard deviation | 221,140.3 ± 163,321.3 | 382,292.8 ± 161,225.9 | |
| Median and [25%; 75%] | 198,973.0 [96,396.5; 296,413.0] | 349,204.5 [245,348.5; 453,839.4] | |
| **VAE 20:2__[M+Na]__4.53** | | | <0.001 |
| Mean ± Standard deviation | 324,940.2 ± 168,661.6 | 552,242.1 ± 158,162.3 | |
| Median and [25%; 75%] | 317,522.5 [217,820.8; 404,969.0] | 586,323.0 [456,010.0; 638,502.9] | |
| **VAE 22:2__[M+Na]__4.96** | | | 0.002 |
| Mean ± Standard deviation | 1,160,756.4 ± 530,800.9 | 1,804,577.0 ± 787,013.6 | |
| Median and [25%; 75%] | 1,167,999.5 [888,934.3; 1,390,874.5] | 1,697,701.3 [1,251,236.0; 2,226,920.0] | |
